# The experimental multi-arm pendulum on a cart: A benchmark system for chaos, learning, and control

**DOI:** 10.1016/j.ohx.2023.e00465

**Published:** 2023-08-07

**Authors:** Kadierdan Kaheman, Urban Fasel, Jason J. Bramburger, Benjamin Strom, J. Nathan Kutz, Steven L. Brunton

**Affiliations:** a Department of Mechanical Engineering, University of Washington, Seattle, WA 98195, United States of America; b Department of Aeronautics, Imperial College London, London, SW7 2AZ, United Kingdom; c Department of Mathematics and Statistics, Concordia University, Montréal, QC H3G 1M8, Canada; d XFlow Energy Company, Seattle, WA, 98108, United States of America; e Department of Applied Mathematics, University of Washington, Seattle, WA 98195, United States of America

**Keywords:** Single pendulum, Double pendulum, Triple pendulum, Pendulum on the cart, Simulink real-time, Dynamical system, Chaos, Nonlinear dynamics, Open access hardware, Open access data

## Abstract

The single, double, and triple pendulum has served as an illustrative experimental benchmark system for scientists to study dynamical behavior for more than four centuries. The pendulum system exhibits a wide range of interesting behaviors, from simple harmonic motion in the single pendulum to chaotic dynamics in multi-arm pendulums. Under forcing, even the single pendulum may exhibit chaos, providing a simple example of a damped-driven system. All multi-armed pendulums are characterized by the existence of index-one saddle points, which mediate the transport of trajectories in the system, providing a simple mechanical analog of various complex transport phenomena, from biolocomotion to transport within the solar system. Further, pendulum systems have long been used to design and test both linear and nonlinear control strategies, with the addition of more arms making the problem more challenging. In this work, we provide extensive designs for the construction and operation of a high-performance, multi-link pendulum on a cart system. Although many experimental setups have been built to study the behavior of pendulum systems, such an extensive documentation on the design, construction, and operation is missing from the literature. The resulting experimental system is highly flexible, enabling a wide range of benchmark problems in dynamical systems modeling, system identification and learning, and control. To promote reproducible research, we have made our entire system open-source, including 3D CAD drawings, basic tutorial code, and data. Moreover, we discuss the possibility of extending our system capability to be operated remotely, enabling researchers all around the world to use it, thus increasing access.

## Highlights

• Presented detailed tutorial on how to build a multi-link pendulum on the cart system.
• Open-sourced design files, 3D CAD files, Simulink files, and control files.
• Open access data sets of the pendulum system from encoders and slow motion camera.
• All files can be downloaded at https://doi.org/10.5281/zenodo.6633719.

## Graphical abstract

**Figure:**
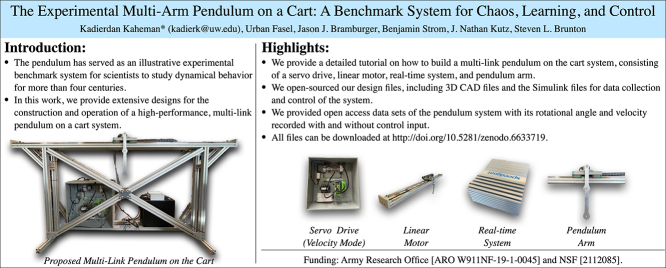

**Table:** 

Specifications table	

Hardware name	The Experimental Multi-Arm Pendulum on a Cart

Subject area	• Mechanical Engineering • Data Collection • Nonlinear Chaotic System • System Identification • Nonlinear System Control

Hardware type	• Single, Double, and Triple Pendulum • Benchmark System

Open source license	Creative Commons Attribution 4.0 International.

Cost of hardware	2729.76 USD without linear motor, real-time system, and slip-rings, etc. For the cost of linear motor, real-time system, and slip-rings, please send a quote to the manufacture.

Source file repository	http://doi.org/10.5281/zenodo.6633719

## 1 Hardware in context

In its simplest form, the single gravity pendulum constitutes a body suspended by a cord or rod that swings back and forth under the influence of gravity [1]. Investigations of this nonlinear system date at least to the seventeenth century and the work of Galileo Galelei [2], [3], [4], with derivations and explanations of the dynamics now commonplace in introductory mechanics courses. Perhaps the most well known application of the single pendulum is in the measurement of time, with Christiaan Huygens’ proposed pendulum clock concept of 1656 being the most accurate time keeping device until the 1930s. The predictable oscillatory motion has also been used to infer the constant of gravitational acceleration, g [5]. As a mechanical benchmark system, the single pendulum has many notable variants: the single pendulum on a cart [6], [7], Foucault’s pendulum [8], [9], Furuta’s pendulum [10], the vertical take-off and landing (VTOL) single pendulum [11], the inertial wheel pendulum [12], [13], the spherical pendulum on a puck [14], and the inverted wheel pendulum [15]. In the eighteenth century, Daniel Bernoulli advanced the study of the pendulum by introducing a second mass suspended by a cord or rod from the first mass, resulting in the *double pendulum*^1^ [16], [17], [18]. Although the gravity pendulum leads to predictable periodic motion, the double pendulum is a prototypical example of a chaotic system [19], [20], [21], [22], requiring specialized numerical integration techniques such as symplectic [23] and variational [24] integrators. The double pendulum has played a central role in the historical development of dynamical systems, attracting the attention of early pioneers, such as Johann Bernoulli and D’Alembert [16]. Notable variants of the system include: the double pendulum on the cart [25], the rotary double pendulum [26], [27], [28], the “acrobot” [29], which is a double pendulum with actuation torque on the second arm, and the “pendubot” [30], [31], which is a double pendulum with actuation torque on the first arm. The double pendulum remains relevant in the study of nonlinear dynamics and has emerged as an important benchmark problem in system identification [11], [32], [33], [34], [35] and machine learning [11], [15], [34], [36], [37], [38], [39], [40], [41].

**Fig. 1:**
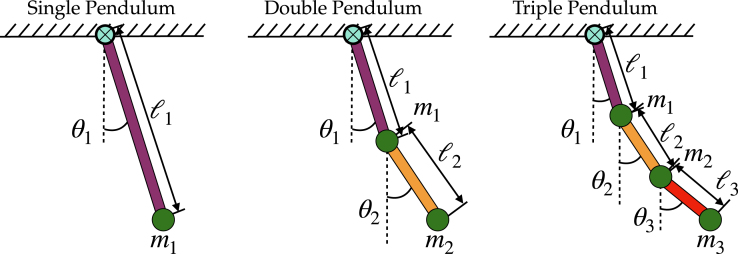
A schematic illustration of the single, double, and triple pendulum.

Beyond the double pendulum, one can continue adding “arms” (masses suspended by rods attached to the previous point masses) to form a chain resulting in the triple pendulum, and so on. The study of such pendulum systems have a long tradition in classical mechanics for a good reason: they are simple mechanical analogs that display much of the rich dynamical behavior observed in far more complex systems. Fig. 1 illustrates the single, double, and triple pendulum.

The single, double, and triple pendulum are also widely used in the control community to develop and test new algorithms. The wide adoption of the multi-arm pendulum as a benchmark problem stems from the simple derivation of the equations of motion, the tunable complexity of behavior, and to the wide applicability in the physical sciences, including to robotics [42], engineering [43], and biology [44], [45]. In the case of the single pendulum, controllers have been designed to swing the pendulum up and/or stabilize it in the inverted position [11], [46], [47], [47], [48], [49], [50], [51], [52], [53], [54], [55], [56], [57], [58], [59]. Similar control methods have been developed to swing up the arms of the double and triple pendulums [25], [28], [58], [60], [61], [62], [63], [64], [65], [66], [67], [68], [69], [70], [71], [72], stabilize their arms in various unstable vertical positions [58], [60], [73], [74], [75], and perform time-periodic motion [26], [27], [31], [76], [77], [78]. Due to the chaotic nature of multi-armed pendulums, the sensitivity increases as more arms are added to the pendulum, thus making it an increasingly difficult control benchmark. The multi-arm pendulum is characterized by the existence of index-one saddle points (i.e., saddle points with exactly one unstable direction), that mediate the chaotic transport of trajectories in phase space; this provides an analog for several more complex systems, such as transport in the solar system [79], [80], [81] and chemical reaction kinetics [82].

With such interest in understanding and controlling the dynamics of pendulums, a number of researchers have build physical models to visualize and test their theoretical calculations; these experimental demonstrations include the single [6], [7], [9], [10], [12], [13], [30], [32], [46], [48], [50], [51], [53], [57], [58], [83], [83], [84], [85], double [19], [21], [25], [29], [30], [31], [58], [67], [86], [87], [88], [89], [90], and triple pendulum [70], [71], [73], [74], [75], [91], [92]. Among these physically-realized pendulums, it is the pendulum on a moving cart that has received the most attention. The general idea is to use the motion of the cart to control and stabilize the motion of the pendulum arms, with benchmark control problems typically focusing on forcing the arms into the unstable upright vertical position. Such a control problem is a proxy for more complex upright stabilization, including human beings standing using their feet as the pivot and applying small muscular adjustments to remain in the upright position. This control procedure is becoming increasingly important for the development of robots that stand upright, and for personal transportation devices, such as self-balancing scooters and single-wheeled electric unicycles. Much like these physical balancing problems, the difficulty in controlling pendulums on a cart usually lies in the physical constraints of the system, such as the pendulum cart having a limited travel range and velocity. Other difficulties come from the specific hardware of the system, the control authority of the cart, and the placement of sensors for real-time measurements of the dynamics. The most common method to measure the rotational angle of each pendulum arm uses encoders and a motion capturing camera [89], [93], with variability in transmitting the collected data using hard wiring or wireless technology [71], [92]. In terms of the actuation of the cart, variants in the physical models use a rotational motor, a servo motor and belt drive, or a linear motor. All of these choices have a profound impact on the robustness of developed control methods.

In this paper we introduce the process and materials needed for constructing a high-performance, fully instrumented, multi-link pendulum on a cart. With regards to the variations on the physical model discussed above, we detail reasons for our choice of sensors, signal transmission, pendulum arm design, and actuation method. In particular, our pendulum on a cart uses an optical encoder and slip-ring to record and transmit the rotational angle of each arm, a linear motor for cart actuation, and Speedgoat and Simulink for the real-time control interface. The goal of this work is to provide a reference guide and tutorial for the construction of the pendulum on a cart system with a flexible design that can further be mounted for nonlinear dynamical demonstrations or easily altered to various other pendulum models to initiate new studies. Moreover, the designed system also servers as an experimental platform to validate the idea of saddle transport using the double pendulum [94]. Our contributions are as follows:

• A detailed tutorial on how to build a multi-link pendulum on a cart system.
• Open-source design files, including 3D CAD files and the Simulink files for data collection and control of the system.
• Open access data sets of the pendulum system with its rotational angle and velocity recorded with and without control input.

These data sets could be of great benefit to the system identification, machine learning, and artificial intelligence communities to test their techniques and algorithms. Finally, we also introduce the concept of cloud experiments so that researchers worldwide can access our model and run experiments without having to build their own system.

We organize our work as follows: In Section 2, we present the major components of the multi-arm cart pendulum system. In Section 3, we show the design process of the pendulum cart, the selection of the real-time control interface, and the electrical components of the system. We then summarize all the deisgn files needed to build the proposed system. In Section 4, the manufacturing and assembling of the proposed system is shown. In Section 5, the operation instruction and the software setup of the system is discussed. Section 5 also explores the idea of cloud experiments. In Section 6, we perform parameter estimation task for the pendulum arm to validate and characterize our proposed system. Finally, Section 7 summarizes the work.

## 2 Hardware description

**Fig. 2:**
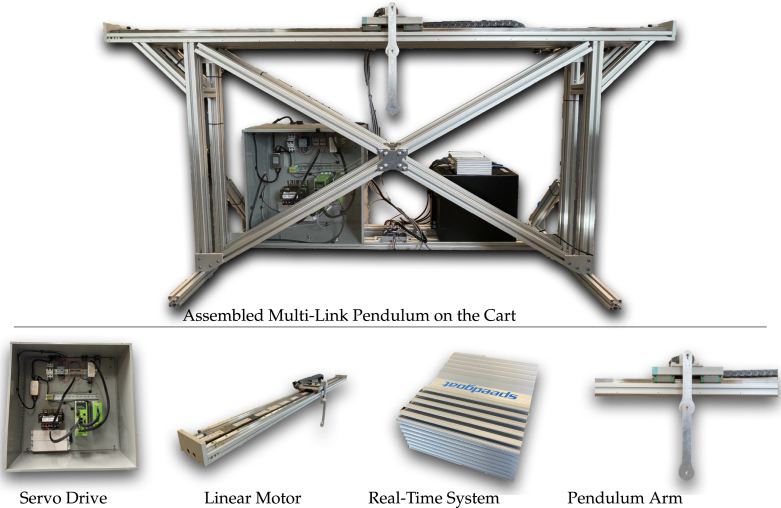
Assembled multi-link pendulum on the cart system. The major components of the system are the servo drive, linear motor, real-time control system, and pendulum arm on the cart.

In this section we introduce the main components, design, and closed-loop control of the multi-link pendulum on the cart system. The system can be used to gather experimental data of a single, double and triple pendulum’s oscillatory motion, and can be used to validate different control laws and system identification algorithms. An overview of the system can be seen in Fig. 2.

The proposed system consists of four major components: (1) the servo drive (motor drive), which provides proper current to the linear motor to move it according to the desired speed; (2) the linear motor with a 1μm resolution magnetic encoder, which provides actuation forces to the pendulum; (3) the real-time system, which controls the motion of the pendulum cart and pendulum arm; and (4) the pendulum arm, which can rotate freely around its joint. The pendulum arm has an optical encoder with 10000 counts per revolution (CPR) to measure its angular position. The four main components are mounted on a frame, as shown in Fig. 2.

**Fig. 3:**
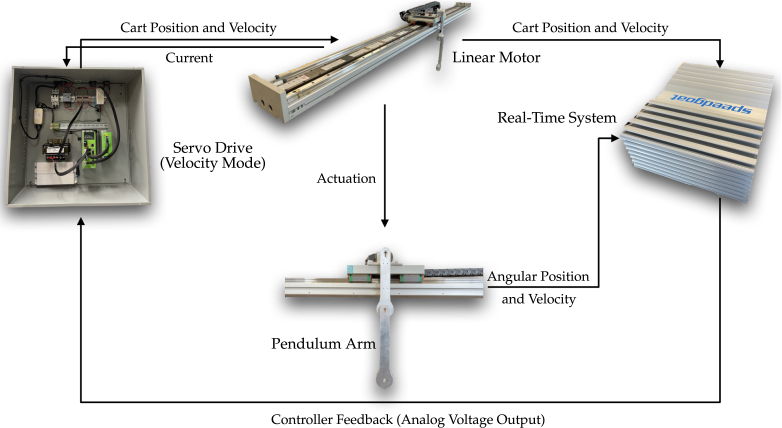
Closed loop control diagram of the proposed system. The linear motor and pendulum arm sends measurement data to the real-time system, which is used to calculate the corresponding control action needed to achieve the control object, such as stabilization, swing-up, stabilizing periodic orbit, etc. After the control command is received by the servo drive as an analog signal, the servo drive controls the linear motor to achieve the desired motion.

In order to form the closed-loop control, the measurements from the pendulum arm and linear motor are sent to the Speedgoat real-time system. The real-time system then runs the user defined controller and calculates the control action needed for the next time step given the control objective. The corresponding control value is then converted to an analog voltage output, and this voltage is sent to the servo drive. In velocity mode, the servo drive measures the voltage of the analog signal generated by the real-time system, and determines the desired velocity of the linear motor. To achieve the desired velocity required by the user, the servo drive uses encoder measurements from the linear motor to calculate its position and velocity. By comparing the actual and the user defined velocity of the linear motor, the servo drive internally^2^ calculates the currents needed to achieve the target velocity. The closed loop control diagram is illustrated in Fig. 3.

Our pendulum design enables simple manufacturing and reliable and accurate operation. It offers several advantages compared to alternative designs: (1) A linear motor overcomes the backlash that can occur in belt-driven cart systems, which poses a challenge for multi-link pendulum control. (2) Using a slip-ring to transfer the electrical signal in the rotating pendulum arm avoids latency that may occur in pendulum designs with wireless transmission. Moreover, using slip-rings avoids adding a battery to the pendulum arm and enables light weight designs [71], [92]. However, using slip-rings results in a more complicated pendulum arm design, which is more difficult to machine. Also, due to the limited number of channels the slip-ring provides (5 or 8 signal wires), it is difficult to add a gyro sensor to measure the acceleration of the pendulum arm. (3) The Speedgoat baseline machine used as our real-time control system fully supports the Simulink Real-Time software, which simplifies the controller validation and testing. In case the system is designed to only study a single pendulum, the Speedgoat machine can be replaced with a less expensive solution, discussed in Sec. A of supplementary material. (4) The same system can be used to study both controlled and uncontrolled behavior, and the user can add/detach pendulum arms to study single, double, or triple pendulums using one system. (5) The pendulum can be detached from the linear motor, and the system can be used to perform other experiments, such as oscillation studies. (6) Different pendulum arm designs can be installed and tested. Thus, the resulting system has great flexibility for future extensions.

## 3 Hardware components and design files

### 3.1 Pendulum arm

The main component of the cart-pendulum system is the pendulum arm. The most simple design consists of an off-the-shelf rod with a mass attached to its end. This design is widely used for single and double pendulums due to its simplicity, but it limits the integration of sensors to measure angular position. For the triple pendulum, it is more common to machine custom aluminum pendulum arms with integrated sensors that can record the rotational angle of the pendulum. In this section, we detail our multi-link pendulum arm design based on custom machined parts with integrated sensors. We introduce the pendulum arm design, and refer to Sec. B of supplementary material for a detailed description of the design and manufacturing of the arm.

**Fig. 4:**
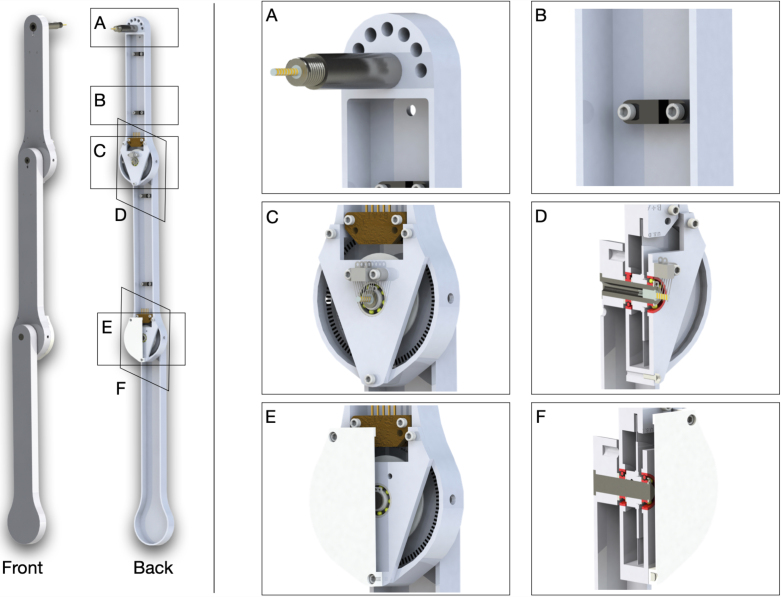
Design of the multi-link pendulum arm. The main components of the assembled pendulum arm are: (1) the pendulum arm body, which is used to install different parts of the pendulum arm, such as sensors, wires, slip-ring, etc.; (2) the pendulum shaft, which is mainly used to support the rotational movement of the pendulum arm; (3) the bearing plate, which is used to secure the pendulum shaft and connect two pendulum arms; and (4) the protection case, which is used to enclose the sensors and slip-ring and prevent them from being damaged.

#### 3.1.1 Pendulum arm design

An overview of our pendulum arm design is illustrated in Fig. 4. The main components of the pendulum arm are: (1) pendulum body; (2) shaft; (3) bearing plate; and (4) 3D printed protection case. The overall structure of the pendulum arm is determined by how it transmits the rotational information measured by the encoders. In our design, a slip-ring sends the encoders’ electrical signals to the real-time system. The advantage of the slip-ring design is the low latency in the signal transmission compared to vision-based and wireless communication systems. Also, no additional computational resources are needed to determine the rotational angle of the pendulum, compared to vision-based tracking systems. This characteristic is particularly beneficial for achieving high sampling rates. One drawback of the slip-ring design is the additional friction on the contact between the slip-ring shaft and brush block. This can be minimized by using a miniature slip-ring and slip-ring brush with gold contact surfaces, which also reduce the electrical noise during the rotational movement of the pendulum arm. An additional challenge of using slip-rings is the requirement for precision machining of the pendulum shaft and also the fixed number of channels to transmit signals. This may reduce the flexibility of the setup if new sensors (e.g. an inertial measurement unit (IMU) sensor) are required for future experiments.

In our design, the shafts of the first and second pendulum arms are hollow to accommodate the slip-ring and the connections to the sensors. The slip-ring wires are clipped to the pendulum arm to prevent twining of the cables. Moreover, two holes on the side of the pendulum arm facilitate the installation of the encoder disk on the pendulum shaft. A stair case shoulder properly secures the bearing that is installed on the first and second pendulum arm. Ceramic bearings are used to minimize the friction during the rotational movement. The advantage of ceramic bearings is that they operate without lubrication. Two bearings are used to fully support the rotational movement of the pendulum arm. To avoid the pendulum shaft sliding out of the pendulum arm during operation, external retaining rings are used to secure the pendulum shaft. The main components of the pendulum arm are manufactured using CNC milling and turning. Finally, the length of the first to third pendulum arms are correspondingly 0.1727, 0.2286, and 0.2413 m. Further details are provided in Sec. B of supplementary material.

**Fig. 5:**
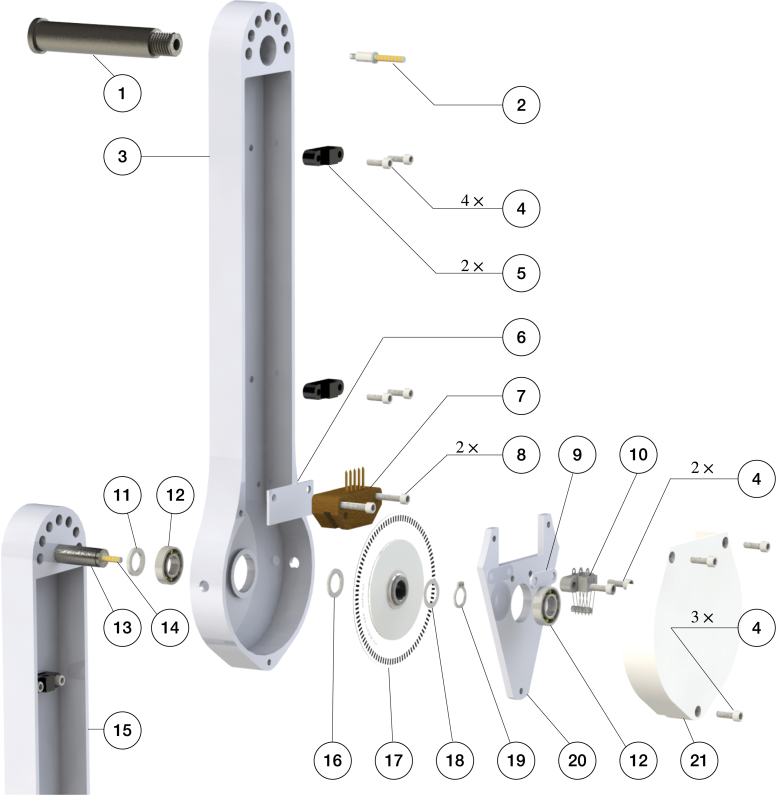
Components and assembly of the first and second pendulum arm.

**Fig. 6:**
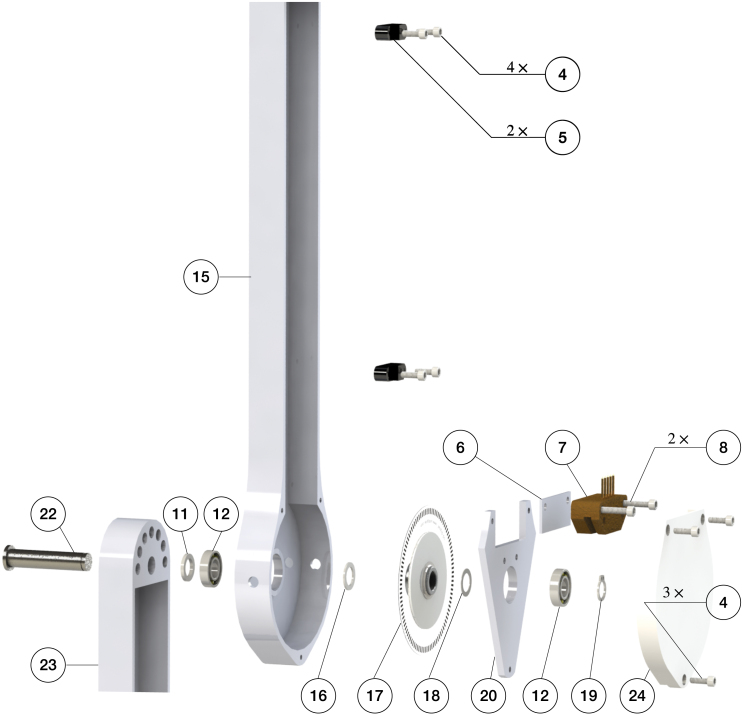
Components and assembly of the second and third pendulum arm.

### 3.2 Pendulum cart

In this section we introduce the design process of the pendulum cart. We first discuss the motor type selection and sizing, and then introduce the design process of the pendulum cart and the bearing house that is used to connect the pendulum arm to the motor, as shown in Fig. 4. The detailed design and manufacturing of the cart and linear motor support frame is introduced Sec. C and D of supplementary material.

#### 3.2.1 Pendulum cart design

**Fig. 7:**
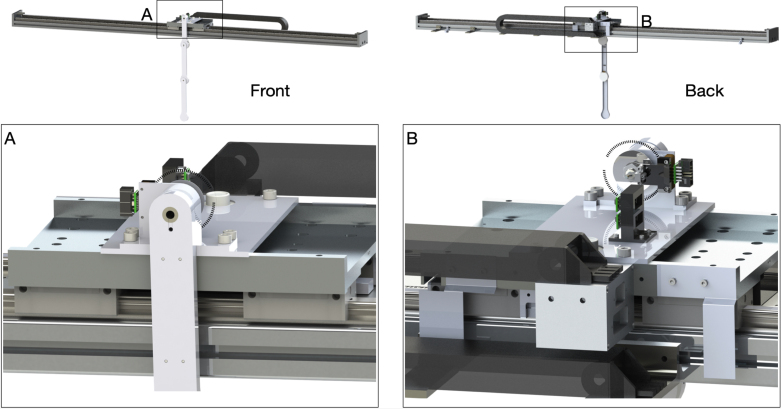
Overall assembly of the pendulum arm and pendulum cart. The main components of the pendulum cart are an aluminum plate and a bearing house.

The pendulum cart has two main functionalities: (1) mounting and supporting the pendulum arm; and (2) providing the actuation to the first pendulum arm to control the system dynamics. The first step in the design of the pendulum cart is the selection and sizing of the motor. Once the motor type and size are selected, a mechanism has to be designed to connect the pendulum arm and motor. Fig. 7 illustrates the designed pendulum cart with the assembled pendulum arm.

The actuation of the pendulum arm is provided by a linear motor. The advantage of using a linear motor is that it does not have backslash issues, which frequently happen in belt-drive type servo motors. This allows accurate control of sensitive maneuvers, such as the swing-up of the double and triple pendulum. Once the type of linear motor is determined, the next step is to size the linear motor. First, the desired maximum speed and acceleration of the cart is defined. To determine the maximum speed and acceleration, we look at the pendulum cart’s desired motion profile. We take the feed-forward trajectory [95] that is needed to swing up the double or triple pendulum. Using this desired motion profile, we determine the peak force and velocity required and use this information to size the linear motor. Here, we determine the pendulum cart’s top speed and acceleration to be 5m/s and $20\;{\mathrm{m/s}}^{2}$. The pendulum arm we designed in Fig. 4 weights less than 0.5kg and the mass of the linear motor stage is 5kg. Thus, the total continuous force provided by the linear motor should be around 110N. We choose a HIWIN linear motor system^3^ LMX1K-SA12-1-2000-PGS1-V103+HS. This linear motor can provide a peak force of 579N with a peak current of $12.7A_{rms}$ and a continuous force of 205N with a continuous current of $4.2A_{rms}$. This motor is powerful enough to provide the desired acceleration and speed. Moreover, the effective stroke of the linear motor is 2m with a magnetic incremental encoder of 1μm resolution.

Once the linear motor has been selected, the next step is to design the connection between the pendulum arm and the linear motor stage. As illustrated in Fig. 7 (A) and (B), a bearing housing is needed to provide support for the first pendulum arm shaft and to secure it so that the pendulum arm can perform free swing. Moreover, an aluminum plate is machined so that the bearing housing can be connected with the linear motor stage. The detailed design of the bearing house is introduced in Sec. C of supplementary material. The final components we design for the pendulum cart are the limit switch plates, as shown in Fig. 7 (B). Fig. 7 (B) shows two limit switch plates installed on the back side of the linear motor stage. They are responsible to block the laser limit switch when the pendulum cart moves to the edge of the linear rail. Same as the pendulum arm, the main components of the pendulum cart are manufactured using CNC milling. The details of the design and manufacturing are provided in Sec. C of supplementary material.

### 3.3 Real-time system

**Fig. 8:**
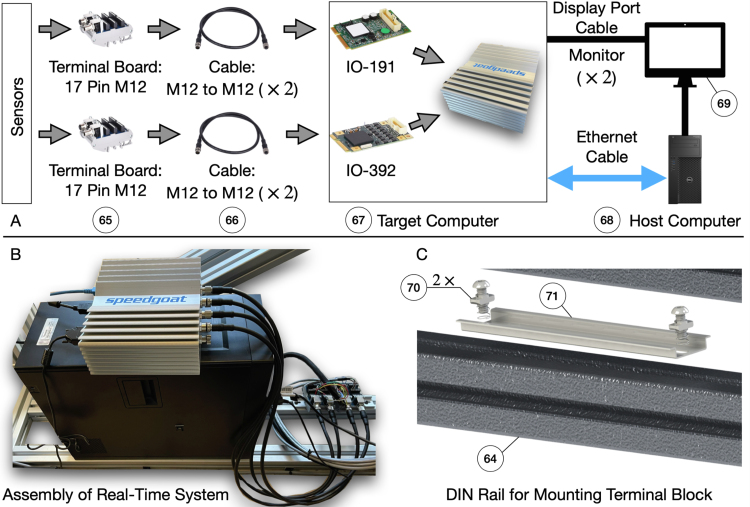
Assembly of the real-time system. (A) rough connection illustration of the target system. The terminal block is used to connect with the sensors. A cable is used to send the signals to the target system. The running status of the target machine is shown on the monitor and the target system and host computer is connected using an Ethernet cable which allows the update of the real-time program to be executed. (B) Assembly of the real-time system. The target machine is place on top of the host machine while the host computer is place on top the system frame. (C) Assembly of the DIN rail that is used to mount the terminal block of the real-time system.

Sections 3.1, 3.2 introduce the major hardware components of the pendulum on the cart system. In this section, we introduce the real-time system that is responsible for: (1) processing the signal sent by the pendulum arm, linear motor, and other sensors; and (2) using the received signal to determine the control action in real-time based on the user program. The major components of the real-time system include: (1) baseline real-time target machine by Speedgoat. (2) User-selected I/O modules, including modules installed inside the real-time system, terminal boards connected to the sensors, and cables connecting the terminal boards and the real-time system. (3) Computer used to program the controller using Simulink Real-Time. (4) Other components such as the power cord, power adaptor, Ethernet cable, software drivers, etc. Fig. 8 (A) illustrates the overall Real-Time system. We use the Speedgoat machine with Simulink Real-Time for several reasons: (1) Matlab and Simulink have a wide application in both industry and academia. (2) Speedgoat and Simulink Real-Time simplify rapid prototyping and hardware in the loop control. (3) The Speedgoat machine has a responsive customer service that helps users to solve their software and hardware problems while using the Real-Time machine. One drawback of the system is that it is comparably expensive. In Sec. A of supplementary material, we introduce an alternative custom-made Real-Time system using the National Instrument Data Acquisition (DAQ) board with Simulink Desktop Real-Time. Other alternatives of the Real-Time system include dSPACE, Typhoon HIL, National Instrument, and others [97].

#### 3.3.1 System choice and specifications

The number and types of I/O channels and the desired sampling frequency determines which specific Speedgoat Real-Time system can be used. In our design, the Real-Time system needs to read at least four quadrature differential encoder signals (three from the pendulum arm and one from the linear motor). We need to have at least two digital input channels to read the limit switch signal, one digital output channel to enable/disable the linear motor drive, and one analog output to control the linear motor in velocity mode. The IO-191-EDU-Baseline as our Digital and Analog I/O module meets these requirements. The IO-191-EDU-Baseline is a 16-bit analog I/O module with eight single-ended or four differential sequential sampling analog inputs. The supported voltage ranges of the analog inputs are ±0.64V, ±1.28V, ±2.56V, ±5.12V, ±10.24V, ±12.288V, ±20.48V, and ±24.576V. Moreover, it has four single-ended analog outputs with supported voltage ranges of ±10V, ±5V, ±2.5V, 0–10V, or 0–5V. Both the input and output range of the analog signal is software configurable. Finally, it also has 16 x general-purpose digital TTL I/O lines. Thus, the module meets our requirements for digital and analog I/O capability. More details can be found in the IO-191-EDU-Baseline manual. As for the encoder reading, we select the IO-392-Baseline configurable FPGA-based I/O module with 50k Artix 7 FPGA and 13x RS422 digital I/O lines. This module can read four quadrature differential encoder signals while providing a 5 V power supply to the encoder sensor. The Pin-Out map for this module while using the driver IO-392-QAD4RS422 can be seen in Table 1 of supplementary material. More details can be found in the user manual.^4^

After selecting the I/O modules, the next step is to determine which specific Speedgoat system is used. This is mainly determined by the sampling frequency, which depends on the user-specific program/algorithm. The desired sampling of the rotational information of the pendulum arm is 5kHz when no control is applied. When stabilizing the single, double and triple pendulum, the sampling rate should be at least 1kHz (using LQR with Kalman filter). According to the user manual of the Baseline Speedgoat system, the I/O latency for reading and sending signals is around 31.5μs when using IO-191-EDU-Baseline and IO-392-Baseline. For the baseline machine, the algorithmic calculation takes about 56μs when running a Simulink model with 1550 blocks and 250 continuous states (equals to 25 Simulink benchmark model F14). Thus, the total latency time is around 87.5μs, which theoretically enables a sampling rate of 11.4kHz, which meets our requirements. Therefore, we choose a baseline real-time target machine by Speedgoat as our real-time controller. After testing, we found a maximum sampling rate of around 12.5kHz for pure data collection, and 5kHz during double/triple pendulum stabilization (using a time-varying LQR controller and a Kalman filter). The sampling rate is problem-specific and using a highly optimized code can further increase the maximum sampling rate. Finally, the host machine used for developing the control law has an Intel(R) Core(TM) i7-3770 CPU with 3.40 GHz frequency and 32 GB of RAM. The host machine runs on Windows 10 Pro with Matlab 2021b.

### 3.4 Electrical system

In this section we introduce the major electrical components of the experimental setup. We introduce the functionality of the components and illustrate the wiring specification of the entire system. The electrical system of the whole setup can be divided into two parts: (1) Linear motor power supply. (2) Pendulum arm sensor system. In the following, we introduce each part separately and illustrate the entire wiring diagram of the electrical system. Warning: None of the authors of this paper are a certified electrician. The wiring diagram of the system is provided as a reference and it has not been examined by an electrician. To the best of the author’s knowledge, the provided wiring diagram is safe to be used but the author’s cannot guarantee its safety.

**Fig. 9:**
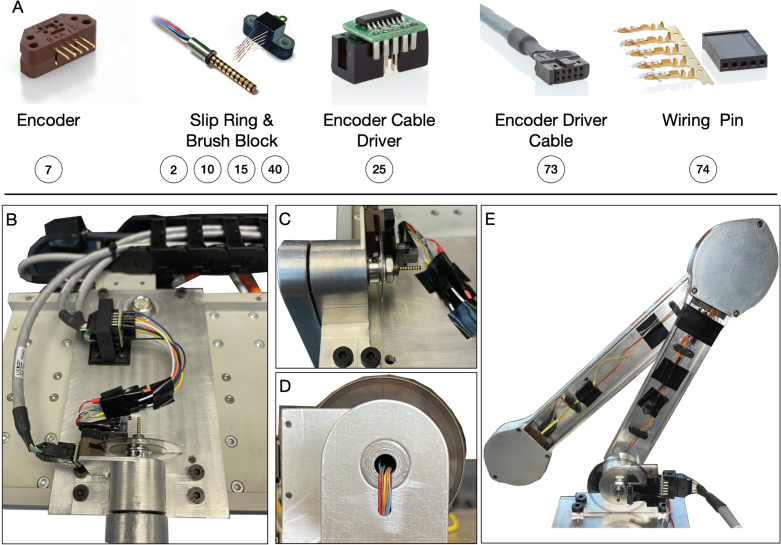
Electrical wiring and assembly of the pendulum arm. (A) Major electrical components of the pendulum arm. (B) connection method of differential driver and encoder reader. (C) to (E) wiring of the slip-ring.

#### 3.4.1 Electrical component of pendulum arm

The electrical component of the pendulum arm mainly consists of an encoder reader (7), slip-ring (2, 15), slip-ring brush block (10, 40), differential driver (25), and differential driver cable (73). Fig. 9 (A) illustrates all those components. The encoder reader’s single-ended A, B, and C/Index channel is transferred into the differential signal using differential drivers to improve the noise robustness of the encoder measurements. Using a differential signal helps to avoid the effect of electrical noise generated by the linear motor. It is generally recommended to use a differential signal whenever possible. Three US Digital CA-C10-SH-NC 10 ft cables are used (differential driver cable) to transfer the differential signals to the target computer. One end of the differential driver cable is connected to a 10-pin female standard (non-latching) connector. This connector is then inserted into the differential driver. The other end of the differential driver cable is unterminated. Thus, it is stripped and inserted into the corresponding channels on the real-time system terminal block (65). This process completes the connection of the differential driver and target system. Last, the differential driver and encoder reader are connected, as shown in Fig. 9 (B).

The first pendulum arm’s encoder reader (measuring the arm’s rotational angle) is directly connected to the differential driver, since the encoder reader is mounted on the bearing housing. This mounting position simplifies the connection of the differential driver to the encoder reader. However, the encoder reader and differential driver connection on the second and third arm are located inside the pendulum arm. This mounting position prohibits the direct connection of the driver and encoder reader. Therefore, two slip-rings are used that connect the encoder reader inside the first and second pendulum arm, as shown in Fig. 9 (E). This allows an indirect connection of the differential driver and encoder reader through the slip-ring brush block, which conducts the encoder reader signal out of the pendulum arm. Next, the breadboard jumper cable connects the slip-ring brush block and differential driver. One end of the breadboard jumper cable is stripped away using a wire stripper and soldered onto the slip-ring brush block. The other end is directly inserted into the differential driver, as shown in Fig. 9 (B). As for the slip-ring, it is first installed onto the pendulum shaft. The unterminated slip-ring wire is striped and then pushed into the pendulum arm using the groove and hole in the front of the pendulum arm, as shown in Fig. 9 (C, D). Inside the pendulum arm, the slip-ring wire is clipped using a 3D printed cable management tool (5). Next, the other end is soldered with the locking clip contact pin, and the locking clip contact pin is insulated with a heat shrink tube to avoid the shortage of the wire connection. Finally, it is connected with the encoder to allow the transmission of the encoder signal through the slip-ring. Fig. 9 (C, D, E) illustrates the slip-ring wiring. Details of the wiring diagram are in Sec. E.1 of supplementary material.

**Fig. 10:**
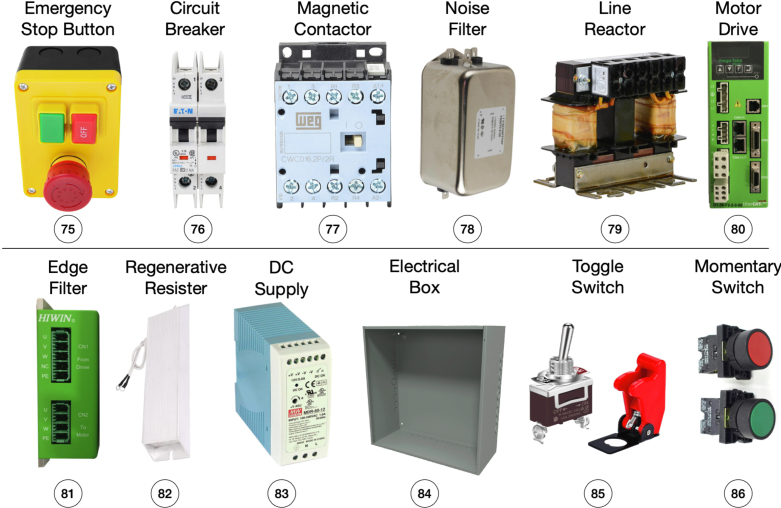
Electrical components needed to provide power supply to the linear motor. An electrical box is used to organize all the components.

#### 3.4.2 Electrical part of linear motor connection

The electrical part of the linear motor mainly concerns the following: (1) properly connecting the motor drive and motor; and (2) connecting the motor drive to the target computer. The connection guidance of the linear motor and linear motor drive is detailed in the user manual of Mega-Fabs D1 Drive.^5^ The user manual also shows the pin out of the control signal cable, which enables the communication between the motor drive and target computer. The main challenge of the electrical part of the linear motor is to reduce the effect of electromagnetic interference (EMI) generated by the linear motor and motor drive. Several techniques can be used for this: (1) use a ground filter to filter out the noise generated by the linear motor and the drive in the ground line; (2) use shielded cables to transmit the signal; (3) use twisted pairs of wires to transmit differential signals; and (4) proper grounding of the all electronic components. With all these techniques combined, EMI is reduced and a proper and safe connection of the linear motor electrical parts is achieved.

Several electrical components are required to connect the linear motor. We use the components that are suggested in the user manual of the D1 motor drive: (1) emergency stop switch; (2) circuit breaker; (3) noise filter; (4) magnetic contactor; (5) line reactor; (6) motor drive; (7) edge filter; (8) regenerative resistor; (9) DC voltage supply; (10) linear motor; (11) electrical box and mounting plate; and (12) other miscellaneous parts that help connecting the electrical components, such as wires, connectors, cables, etc. A summary of the major electrical components can be seen in Fig. 10. The details of these parts are introduced in Sec. E.2 of supplementary material, along with a detailed wiring diagram. The user can control the linear motor in both position and velocity mode. In position mode, a reference encoder signal is supplied to the servo drive and converted into the linear motor’s relative position. The servo drive will control the linear motor to reach the desired target position determined by the reference signal. In velocity mode, an analog signal is converted to the linear motor’s target velocity, and the servo drive will control the linear motor to reach the target speed. In practice, the pendulum cart is controlled under the velocity mode, and the user needs to provide the analog signal corresponding to the desired target speed to the servo drive. For more details, please see Sec. F of the supplementary material.

### 3.5 Design files summary

**Table:** 

Design filename	File type	Open source license	Location of the file

3D Printed Parts	STL files	Creative Commons Attribution 4.0 International	https://zenodo.org/

Drawings	PDF and SLDDRW	Creative Commons Attribution 4.0 International	https://zenodo.org/

Parts	SLDPRT and SLDASM	Creative Commons Attribution 4.0 International	https://zenodo.org/

Datas	MAT file	Creative Commons Attribution 4.0 International	https://zenodo.org/

Video Data	ZIP file	Creative Commons Attribution 4.0 International	https://zenodo.org/

Demo code	SLX file	Creative Commons Attribution 4.0 International	https://zenodo.org/

• **3D Printed Parts**: The 3D printing file for some of the parts needed in the whole system assembly.
• **Drawings**: PDF and SLDDRW files that details the dimensions of the machined parts.
• **Parts**: SLDPRT and SLDASM 3D CAD model files that shows the assembly of the entire system.
• **Datas**: Folder that contains the MAT file which records the rotational data of the pendulum arm using encoders.
• **Video Data**: Folder that contains the slow motion data of the pendulum.
• **Demo code**: Folder that contains the demo Simulink file which can be used to collect data from the pendulum system.

**Fig. 11:**
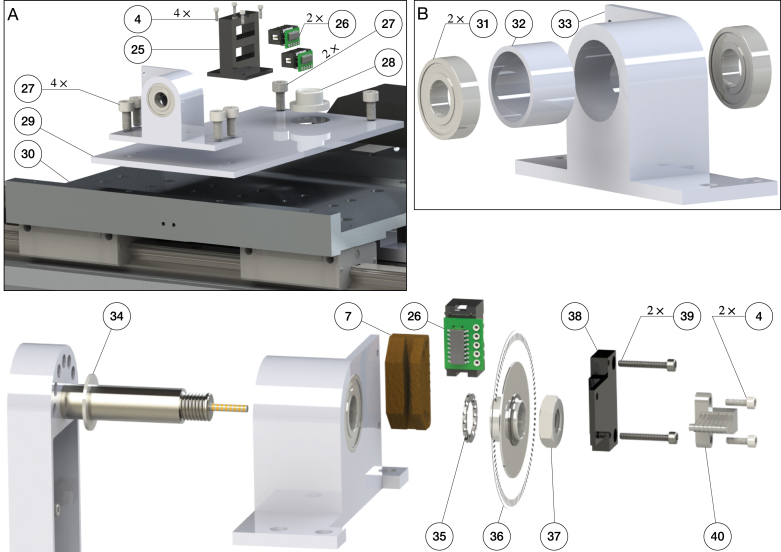
Assembly process of the pendulum cart, pendulum arm and bearing housing. (A) Installation of the bearing housing, cart plate and linear motor stage. (B) Assembly of the ceramic bearing and the bearing housing. (Bottom) Installation of the pendulum arm and bearing housing.

## 4 Build instructions

### 4.1 Pendulum arms assembly

The assembly of the pendulum arms is shown in Fig. 5, Fig. 6. The pendulum body and bearing plate are assembled first. In order to assemble the pendulum arm body, the following steps are performed: (1) the bearing (12) is installed to the pendulum arm body (3, 15) (the transitional fit between the bearing and bearing hole should facilitate the assembly). To make sure the bearing stays in place, a thin layer of Loctite is applied to the outer ream of the bearing before it is push into the bearing hole. Once the bearing is installed, a paint tape is used to cover the bearing to prevent dust entering the assembly. (2) The pendulum shaft (1, 13, 22) is press fitted into the pendulum arm body (3, 15, 23). To make sure the installation is smooth, it is recommended to lubricate the pendulum shaft and shaft hole before the press fit. (3) The slip-ring (2, 14) is then installed onto the pendulum shaft (1, 13). Those steps complete the assembly of the individual pendulum arm. The assembly of the bearing plate follows a similar process. The outer ream of the bearing (12) is applied with a thin layer of Loctite, then pressed into the bearing plate (20). This step should also be straightforward, since the fit tolerance between the bearing and bearing plate is a transitional fit.

To assemble the double pendulum (first and second arm), the following steps are performed: (1) the shim (11) is slid onto the shaft of the second arm (13). Next, the pendulum shaft is slid into the bearing of the first arm (12) until the shim (11) contacts the inner ring of the bearing. (2) The shim (16), encoder disk (17), and another shim (18) are slid onto the second arm’s shaft (13). Then, the assembled bearing plate is slid onto the shaft (13) as well. Finally, the external retaining ring is clipped onto the shaft (13). This should hold everything together while still allowing adjustments, since the bearing plate is not screwed yet. (3) The 3D printed shim (6) and the encoder reader (7) are pushed onto the desired place by aligning the holes. Then, the screws (8) are positioned. (4) The 3D printed shim (9) and slip-ring brush block (10) are installed by aligning the holes on the bearing plate, and the screws (8) are tightened. After this step, the contact between the slip-ring and slip-ring brush block should be carefully observed. The slip-ring brush should be centered to its corresponding channel. (5) Next, the protection case is installed by aligning the holes on the pendulum arm body, bearing plate, and protection case. The screws (4) are mounted to fasten the assembly. (6) Finally, the wire clipper (5) is installed onto the pendulum arm (3) by aligning the holes, and the screws (4) are tightened. The above steps finish the assembly of the first and second pendulum arm. Fig. 6 shows the assembly of the triple pendulum arm, following similar steps as for the assembly of the double pendulum arm. The difference though is that the third arm’s shaft does not have a slip-ring installed. In Sec. B of supplementary material the detailed steps to assemble the triple pendulum arm are introduced.

### 4.2 Pendulum cart assembly

The assembly of the cart plate, bearing housing, pendulum arm, and the linear motor stage is illustrated in Fig. 11. First, the bearing housing is assembled, which includes two steps: (1) a 3D printed spacer (32) is press-fitted into the bearing hole (33). (2) A thin layer of Loctite is applied to the outer ream of the bearing (31). Then, the bearing is installed on the backside of the bearing house. Next, the same process is repeated to install the bearing on the front side. It is important to avoid excess Loctite to enter the bearing, which may damage it. Once finished, the bearing housing is left for 24 hours to ensure the bearing is securely installed. This process can be seen in Fig. 11 (B). Next, the cart plate is assembled. First, the circular level indicator (28) is installed to the cart plate (29). To secure it, a thin layer of Loctite is applied to the outer ream of the circular level. Next, the 3D printed cable management tool (25) is screwed (4) to the cart plate (29), and the differential driver (26) is installed on the cable management tool (25). Then, the cart plate (29) is mounted to the linear motor stage (30) using 4 screws (27). This completes the installation of the cart plate to the linear motor, as shown in Fig. 11 (A).

The installation of the pendulum arm to the bearing housing is shown in Fig. 11. (1) The spring steel ring shim (34) is slid onto the first pendulum arm shaft (1). Then the shaft (1) is slid into the bearing (31) until the shim contacts the inner ring of the bearing. (2) A spacing shim (35) is placed onto the shaft until it contacts the inner ring of the bearing (31). The encoder disk (36) is installed onto the shaft, which allows the measurement of the first arm’s rotational angle. (3) A nut (37) is tightened using the thread on the first pendulum arm. This allows the application of axial force to the bearing housing, which helps to reduce the oscillation of the pendulum arm on the axial direction. The nut should not be tightened too much, to prevent damage of the bearing and to reduce the friction between the bearing ball and bearing case. (4) The encoder reader (7) is placed so that its holes are aligned with the holes on the bearing housing. Next, the holes are aligned on the 3D printed slip-ring brush base (38) with the holes on the bearing housing. Once the holes are aligned, the bearing housing, encoder reader, and 3D printed slip-ring brush block base are mounted with screw (39). (5) The differential driver (26) is installed onto the encoder reader (7). (6) The slip-ring brush block (40) is installed onto the 3D printed base (38) with screw (4). It is important to make sure that each brush is centered with the corresponding channel on the slip-ring (2). The above steps complete the assembly of the pendulum arm and the linear motor.

**Fig. 12:**
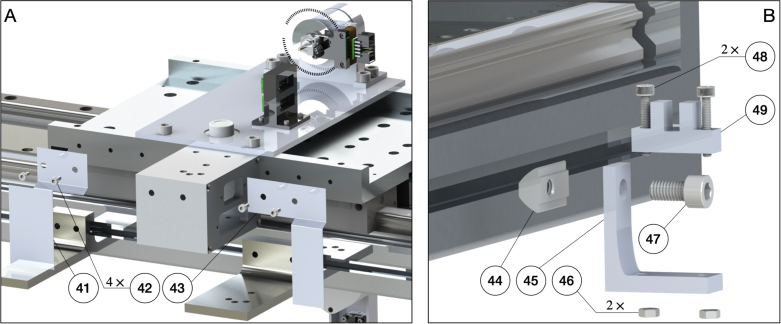
Assembly of the limit switch plate and limit switches.

The assembly of the limit switch plate (41,43) and the linear motor stage (30) is shown in Fig. 12 (A). The left and right limit switches (49) are connected to the linear motor frame with the 3D printed limit switch base (45). The 3D printed base (45) is first connected with the linear motor frame using drop in T-slotted framing fasteners (44) and screws (47). Next, the limit switch (49) is connected with the 3D printed base using screws (48) and nuts (46). The detailed assembly process is illustrated in Fig. 12 (B).

### 4.3 Real-time system assembly

The assembly of the real-time system is straightforward, as shown in Fig. 8 (B). The host computer (68) is directly placed on the aluminum extrusion (64), while the target computer (67) is placed on top of the host computer. The Speedgoat target machine can be mounted on the system frame following the steps specified in the user manual of the real-time system. By directly placing the host and target machine on the system frame, the center of mass of the whole system is lowered, making the system frame more stable. A DIN rail (71) is mounted on the system frame (64) using a drop-in fastener. This DIN-rail allows the mounting of the terminal block (65). The bill of materials of the real-time system can be found in Table. 8 in Sec. J of supplementary material.

## 5 Operation instructions

The operation procedures of the system include pre-experiment preparations, operations required during the experiments, and post-experiment operations. During the pre-experiment preparations, the operator should check all the wiring connections of the system and make sure there is no electrical shortage and hardware damage before starting. Then the controller file is prepared, and the system is turned on while ensuring safety. The main operation that must be performed during the experiment is to check whether the experiments are going as planned. If not, the operator should stop the experiments immediately and cut off all the power supply to the system. After the experiment, inspect any damage to the setup and then cut off all power to avoid any electrical hazards. For a detailed step-by-step operation guidance see Sec. G of supplementary material. While operating the system, safety should always be the number one priority. To ensure the safe use of the experimental setup, mechanical, electrical, software, and personal safety measures must be implemented. A critical mechanical safety measure is to install a shock absorber on the side of the linear motor rail. This will help absorb the extra kinetic energy of the linear motor in case of controller failure. We further recommend purchasing a protective panel to surround the experimental setup. This can reduce the risk of personal injury caused by pendulum parts detaching while the linear motor is in motion. Sec. H of supplementary material describes other safety measures that must be followed. Besides the safety notes in this paper, the reader must also follow all the safety instructions written in the individual components’ user manuals. We also emphasize that the proposed design of the multi-link pendulum on a cart has been tested and used safely in the lab environment by the authors. One should always exercise caution when building and operating the system and none of the authors can be held responsible for any damage or injury caused by reproducing the design shown in this paper. We close this section by mentioning the software setup of the system and the concept of accessing the experimental hardware through the cloud. The latter can help the interested user avoid the trouble of building and maintaining such a delicate system, improve the research efficiency, and provide a standard test bench for comparing different controllers.

### 5.1 Software setup

Two major software packages are used for successful and safe experiments: (1) The HIWIN Lightning software and (2) Simulink Real-Time model. The former is used to set up the parameters of the linear drive, while the latter is used to develop the real-time control algorithm for the system. The motor drive’s Lightning software allows the selection of motor type and motor parameters, for which the motor drive uses this information to determine the control parameters needed to move the linear motor in the desired motion profile. The Lighting software also configures the linear motor’s encoder reading, allows the setup of the linear motor’s Hall sensor, allows the user to select which in mode the linear motor should operate (position, velocity, force/torque, or stand-alone mode), configures the programmable I/Os of the D1 drive’s CN2 channel, and finally, can set up software safety mechanisms. Sec. F.1 of supplementary material enumerates the details to setup these functionalities. The Simulink Real-Time software is used to develop the controller for the real-time pendulum experiments. To read the sensor signal and output control signal, the Simulink Real-Time and Speedgoat needs to be configured properly. The main aspects to be considered when setting up the Simulink model are: (1) setting up the Simulink blocks to allow the desired digital I/O functionalities, (2) setting up the FPGA module to enable encoder reading, (3) utilizing the digital I/Os to start the linear motor and stop it when the limit switches are triggered, (4) switching of the operating mode of the linear motor drive and activating homing function, (5) sending the analog signal to control the velocity of the linear motor and (6) reading encoder sensors. These aspects are detailed in greater depth in Sec. F.2 of supplementary material.

### 5.2 Cloud access to experiments

**Fig. 13:**
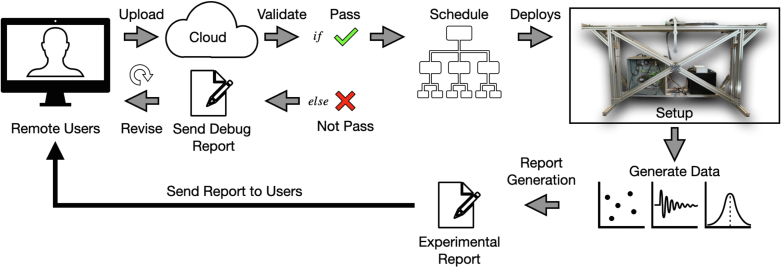
Overall schematic of a cloud experiment of the pendulum on the cart system.

Fig. 13 shows the remote cloud experiment concept. This is similar to cloud services where infrastructure, hardware, or software can be accessed by remote users through the internet. In our case, the user accesses the pendulum hardware. The user must first develop their own program to achieve some objective using the experimental system. The program could be a controller to move the pendulum cart for data collection or for a control objective. This program should be coded according to a given template. After the user uploads their program to the cloud, it is necessary to test for any compiling errors and controller errors. Testing whether the proposed controller is safe is a difficult task. For example, some controllers might require the pendulum cart to move at unreasonable speeds or accelerations. If the controller is unstable, then it might damage the experimental setup. To avoid this, it may be necessary to test the controller in a digital twin of the pendulum. If position, velocity, or acceleration limits are violated, then a debug report will be sent to the user to allow revision and resubmission. Once the user program passes all tests on the digital twin, it may be deployed on the real experimental system. In the physical experiment, failsafe hardware limits may also be imposed, operating outside the user specified code.

During the experiment, the system should record all available data, including pendulum arm rotational angle and angular speed, linear motor position and velocity. The system should also record user defined variables. The collected data will be sent to the user to allow further analysis. A video camera will also record the experiments. The user will then be responsible for determining whether the desired objective is achieved by reviewing the recorded data and videos. The user should also be allowed to send a bug report to allow the maintenance of the hardware system.

There are several challenges that must be addressed for the cloud experiments. First is to deploy the user program to the real-time system automatically. In the current design, we use the Speedgoat machine as our real-time controller. Every time a controller is deployed, the user manually starts Simulink. A pipeline to automatically read, load, compile, and start the code must be developed. The second challenge is to automatically self-check the system. The real-time system should be able to perform the self-check and determine if any components must be replaced.

## 6 Validation and characterization

Estimating the parameters of the pendulum system requires data collected from the designed system, and it is a perfect task for validating and characterizing the performance of the proposed system. An optimization problem is solved to find the pendulum system’s parameters that best predict the data. This also requires an analytical model of the pendulum, which is shown in Sec. I of supplementary material. Parameter estimation is a standard task in pendulum control experiments where the model of the system is needed along with its parameters, while the model derivation of the single, double, and triple pendulum all follows through a similar process. We omit the model derivation for single and triple pendulums since they are similar. The former is standard and the latter can be derived through similar methods to the double pendulum [63], [70], [89].

**Fig. 14:**
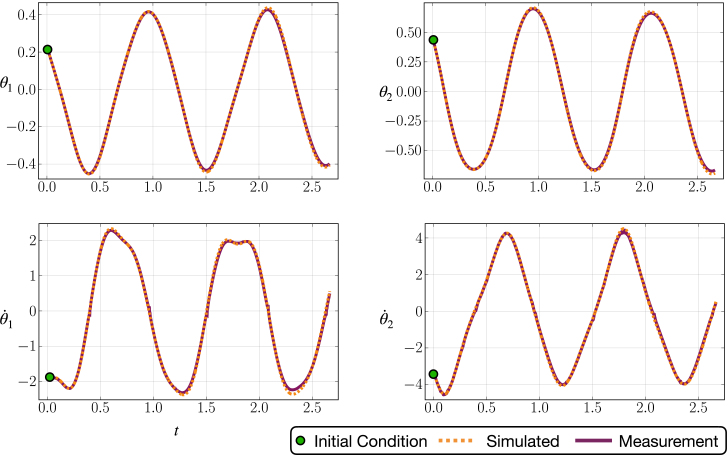
A comparison of the simulated double pendulum dynamics with the optimal parameter values shown in Eq. 10 and Eq. 14 of supplementary material to data gathered from the physical model.

In the case of the experimental pendulum, there are several parameters that must be identified, including the mass of the pendulum arm m and the position of center of mass, defined as a. We also require the length of the pendulum arm l and the inertial of the pendulum arm J, as depicted in Fig. 1. Although frequently ignored in parameter estimation, we also seek to determine the local constant of gravitational acceleration, g, which plays an important role on the chaotic dynamics of the double and triple pendulum. Some parameters do not show up in the derived equation of motion of the cart-pendulum, such as the mass of the cart M when the control is taken to be the acceleration of the cart. We summarize these definitions using the double pendulum as an example in Fig. 26 and Table. 4 of supplementary material provides details of the estimated pendulum arm parameters. Finally, Table. 4 also provides the values of the friction coefficients ${ɛ}_{i}$ which lead energy dissipation in the physical system. Fig. 14 compares the identified and measured system dynamics, showing a good match between the two. Moreover, a good match of the identified and actual dynamics can be important for stabilization and other types of control of the multi-link pendulum [63], [70], [71], [78], [92]. Due to the page limit, we omit the result of performing such tasks as it varies based on the tasks and controllers employed. Interested readers can find our demo experiment stabilizing the double pendulum on the cart system using the link below^6^. For more details on the method of parameter estimation, please see Sec. I of supplementary material for an exposition with the double pendulum.

## 7 Conclusions and discussion

In this paper, we have introduced an experimental multi-link pendulum on a cart system. This system can be used to collect experimental data from the single, double, and triple pendulum. Moreover, the user can control the pendulum motion by actuating the cart via a linear motor. This makes the experimental system a powerful tool for studying the control of chaotic systems. Our experimental setup is open source, with all the detailed design choices and CAD files freely available, which allows reproduction of the system. We have also collected experimental data sets of the single, double, and triple pendulum and made them freely available. We believe this data will be valuable for the machine learning and modeling communities to test various algorithms. All code and design files can be downloaded on our repository page.^7^ As a validation of our experimental setup, we showcased the parameter estimation task of the double pendulum with more details provided in Sec. I of supplementary material. Moreover, the source file repository provides data, code, and data collection files necessary for the parameter estimation task. Our future work includes providing detailed tutorials and files related to some of the standard tasks in pendulum research, such as stabilization and swing-up of the multi-link pendulum on the cart system.

To make it possible to reproduce this pendulum on a cart system, we have provided a detailed description of the assembly and manufacturing process. Furthermore, the wiring diagrams of the electrical components and the software set up are also documented. Although the detailed manufacturing process of our setup is documented, we realize that many labs may not have the time and funding to replicate this system. Beyond this, the time needed to manufacture the experiment and the effort needed to maintain it may not be worth the investment for groups that only need occasional use of the experimental setup. This high cost can be mitigated by using more standard parts and 3D printing technology, but it does not solve the maintenance effort and the time consuming manufacturing and assembly process. This drawback motivates a future modification to our experimental setup to allow cloud access for remote users, discussed in Section 5, so that they can remotely access the system for data collection and control experiments. To summarize, this manuscript introduced an experimental multi-arm pendulum on a cart system: A benchmark system for chaos, learning, and control. For a complete bill of materials, please reference Sec. J of supplementary material.

## CRediT authorship contribution statement

**Kadierdan Kaheman:** Conceptualization, Methodology, Software, Validation, Formal analysis, Investigation, Data curation, Writing – original draft, Writing – review & editing, Visualization. **Urban Fasel:** Validation, Investigation, Writing – review & editing. **Jason J. Bramburger:** Validation, Formal analysis, Investigation, Writing – review & editing. **Benjamin Strom:** Conceptualization, Methodology. **J. Nathan Kutz:** Conceptualization, Methodology, Resources, Writing – review & editing, Visualization, Supervision, Project administration, Funding acquisition. **Steven L. Brunton:** Conceptualization, Methodology, Resources, Writing – review & editing, Visualization, Supervision, Project administration, Funding acquisition.

## Declaration of competing interest

The authors declare that they have no known competing financial interests or personal relationships that could have appeared to influence the work reported in this paper.

## Appendix A Supplementary data

MMC S1 The supplementary material includes a detailed description of the manufacturing, assembling, and software setup of our experimental multi-arm pendulum on the cart system. It also includes a complete bill of materials, step-by-step operation details, and safety protocols that should be implemented when using the system.

## Footnotes

1 The double pendulum can also be referred to as a compound or linked pendulum.

2 To the best of our knowledge, the servo drive uses a PID controller to achieve the target speed set by the user.

3 See Appendix A of the HIWIN linear motor system manual document “Linear Motor System(EN).pdf” [96].

4 All the user manuals can be found on https://github.com/dynamicslab/MultiArm-Pendulum.

5 Available on https://github.com/dynamicslab/MultiArm-Pendulum.

6 Video showing the stabilization of the double pendulum on the cart system.

7 Code available at http://dx.doi.org/10.5281/zenodo.6633719.
