# Supplementary material for: The experimental multi-arm pendulum on a cart: A benchmark system for chaos, learning, and control
Source: HardwareX. 2023 Aug 7;15:e00465. doi: 10.1016/j.ohx.2023.e00465 (PMC10458325; doi:10.1016/j.ohx.2023.e00465)
Supplement: MMC S1 — The supplementary material includes a detailed description of the manufacturing, assembling, and software setup of our experimental multi-arm pendulum on the cart system. It also includes a complete bill of materials, step-by-step operation details, and safety protocols that should be implemented when using the system. [file mmc1.pdf]

Supplementary Material of "The Experimental Multi-Arm Pendulum on a Cart: A Benchmark System for Chaos, Learning, and Control"

Kadierdan Kaheman[1,*], Urban Fasel[2], Jason J. Bramburger[3], Benjamin Strom[4], J. Nathan Kutz[5], Steven L. Brunton[1]

[1] Department of Mechanical Engineering, University of Washington, Seattle, WA 98195, United States of America

[2] Department of Aeronautics, Imperial College London, London, SW7 2AZ, United Kingdom

[3] Department of Mathematics and Statistics, Concordia University, Montréal, QC H3G 1M8, Canada

[4] XFlow Energy Company, Seattle, WA, 98108, United States of America

[5] Department of Applied Mathematics, University of Washington, Seattle, WA 98195, United States of America

Contents

A. Real-Time System Using Simulink Desktop Real-Time **2**

B. Pendulum Arm Details **2**
- B..1 Pendulum Arm Design Details . . . 2
- B..2 Pendulum Arm Manufacturing Details . . . 4
- B..3 Assembly Details of Second and Third Arm . . . 6

C. Pendulum Cart Details **6**
- C..1 Pendulum Cart Manufacturing Details . . . 8

D. System Frame Details **9**
- D..1 System Frame Design Details . . . 10
- D..2 System Frame Manufacturing Details . . . 10
- D..3 System Frame Assembly Details . . . 10

E. Electrical System Details **13**
- E..1 Pendulum Arm Wiring Details . . . 13
- E..2 Components Choice of Linear Motor's Electrical System and Wiring Details . . . 13

F. Software Setup Details **19**
- F..1 Software Setup of Motor Drive Details . . . 19
- F..2 Software Set Up of the Simulink Model Details . . . 26

G. Step by Step Operation Details **33**
- G..1 Pre-Experiment Preparations . . . 33
- G..2 Peri-Experiment Operations and Precautions . . . 34
- G..3 Post-Experiment Operations . . . 35

H. Safety Mechanism and Notes Details **35**

I. Parameter Estimation Details **37**
- I..1 Equations of Motion . . . 37
- I..2 Parameter Estimation . . . 39

J. Bill of Materials **41**
- J..1 Bill of Materials for Building Pendulum Arm . . . 41
- J..2 Bill of Materials for Building Pendulum Cart . . . 42
- J..3 Bill of Materials for System Frame . . . 44
- J..4 Bill of Materials for Real-Time System . . . 44
- J..5 Bill of Materials for the Electronic Parts . . . 45

* Corresponding author (kadierk@uw.edu); Code availalbe at https://github.com/dynamicslab/MultiArm-Pendulum

A. Real-Time System Using Simulink Desktop Real-Time

Besides using the Speedgoat Real-Time machine and Simulink Real-Time, it is possible to use a low-cost alternative as the Real-Time system. This solution uses the National Instrument Data Aqcusition (DAQ) device PCIe-6341 as the data collection device (I/O module). PCIe-6341 can read up to four quadrature encoders at the same time. Moreover, it has multiple digital I/Os and analog I/Os. For more details on the device specification, please check the user manual. In this setup, the sensors are connected to the National Instrument terminal connector SCB-68A. Then, the terminal connector and DAQ are connected using cable SHC68-68-EPM. This connection completes the sensor data acquisition task. To read sensor signals in real-time and send control command (analog signal) to the motor drive (velocity mode), the Simulink Desktop Real-Time is used as the real-time operating system. In this case, only one computer is needed to develop the user program and perform real-time control. This real-time system setup has been tested in our previous paper [1]. When using Simulink Desktop Real-Time, the real-time program only runs on a single core instead of multi-cores as Simulink Real-Time does. This drawback decreases the maximum sampling frequency of the Simulink Desktop Real-Time setup. Our previous experiments show the maximum sampling rate in pure data-collection mode is around $5kHz$ when using Simulink Desktop Real-Time with the host machine we mentioned in Sec. 3.3. The maximum sampling rate of the double pendulum stabilization task (using time-varying LQR and Kalman filter) is around $2kHz$ in this case.

B. Pendulum Arm Details

B..1 Pendulum Arm Design Details

An overview of our pendulum arm's design is described in Sec. 3.1. Here, we describe the design of the pendulum arm in more detail. The main components of the pendulum arm are: 1) pendulum body, 2) shaft, 3) bearing plate, and 4) 3D printed protection case. The overall structure of the pendulum arm is determined by how it transmits the rotational information measured by the encoders. In our design, a slip-ring sends the encoders' electrical signals (mainly the A-, B-, and I-channel signals) to the real-time system, and conducts currents to the encoder sensors on the rotational component. The slip-ring design introduces friction on the contact between the slip-ring shaft and brush block. To minimize this additional effect of friction, a miniature slip-ring and slip-ring brush are used with gold contact surfaces that reduce the electrical noise during the rotational movement of the pendulum arm. Other designs based on vision tracking or wireless technology to measure the pendulum arm rotational angle could avoid these additional friction effects. However, the slip-ring design is simple and does not have latency in the signal transmission that occur in vision-based and wireless communication type pendulum arms. Also, no additional computational resources are needed to determine the rotational angle of the pendulum compared to vision-based tracking systems. This characteristic is particularly beneficial for achieving higher sampling rates. The challenge of using a slip-ring is that it requires precision machining of the pendulum shaft. Moreover, the slip-ring has a fixed number of channels to transmit signals. This may reduce the flexibility of the setup if new sensors (e.g. an inertial measurement unit (IMU) sensor) are required for future experiments. To accommodate the slip-ring and to connect it with the sensors, the first and second pendulum arm's shaft is designed to be hollow, as shown in Fig. 1. As the first pendulum arm shaft takes most of the static and dynamic load, it has a larger diameter than the second and third pendulum arm shafts. Moreover, to minimize the axial oscillation of the pendulum arm, a thread is machined on the first pendulum shaft such that the axial load of the

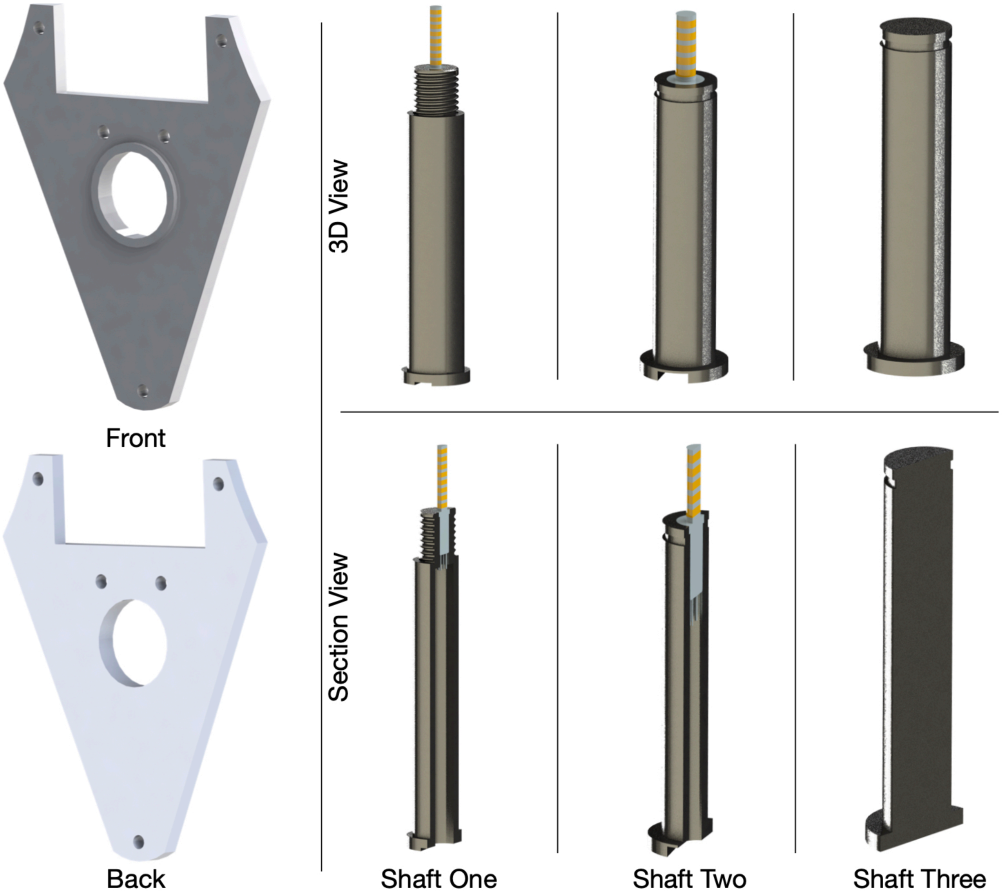

Figure 1: Design of the bearing plate and pendulum arm's shaft. The bearing can be installed on the bearing plate and together support the free rotational movement of the pendulum shaft and pendulum arm. The pendulum arm's shaft is designed hollow to accommodate the slip-ring wires. The groove on the bottom of the pendulum shaft is used to place guide the slip-ring wires into the pendulum arm.

pendulum arm is adjustable. When more axial force is applied, we can reduce the axial oscillation of the pendulum arm.

The slip-ring wires enter the pendulum arm through a small hole on the front side of the pendulum arm, near the position where the pendulum shaft is mounted, as shown in Fig. 2 (A). The slip-ring wire enters the pendulum arm through this hole and connects with the encoder located on the backside of the pendulum arm. The groove in Fig. 2 (A) is aligned with the groove on the pendulum arm's shaft, as shown in Fig. 1. Together, they guide the slip-ring wire and provide space to store it and avoid the cable to stick out of the front face of the pendulum arm. To properly manage the slip-ring wires inside the pendulum arm, a 3D printed cable clip is designed, shown in Fig. 4 (B) of the paper. This cable clip holds the slip-ring wire to its place and prevents twining of the slip-ring wire. To reduce the weight of the pendulum arm, several holes are drilled near the shaft hole, indicated in Fig. 2 (B). Moreover, two holes on the side of the pendulum arm facilitate the installing of the encoder disk on the pendulum shaft, shown in Fig. 2 (C). In Fig. 2 (C), the stair case shoulder is shown that properly secures the bearing that is installed on the pendulum arm. This stair case shoulder is not needed in the third pendulum arm since no other pendulum arm is attach to it, shown in Fig. 2 (D).

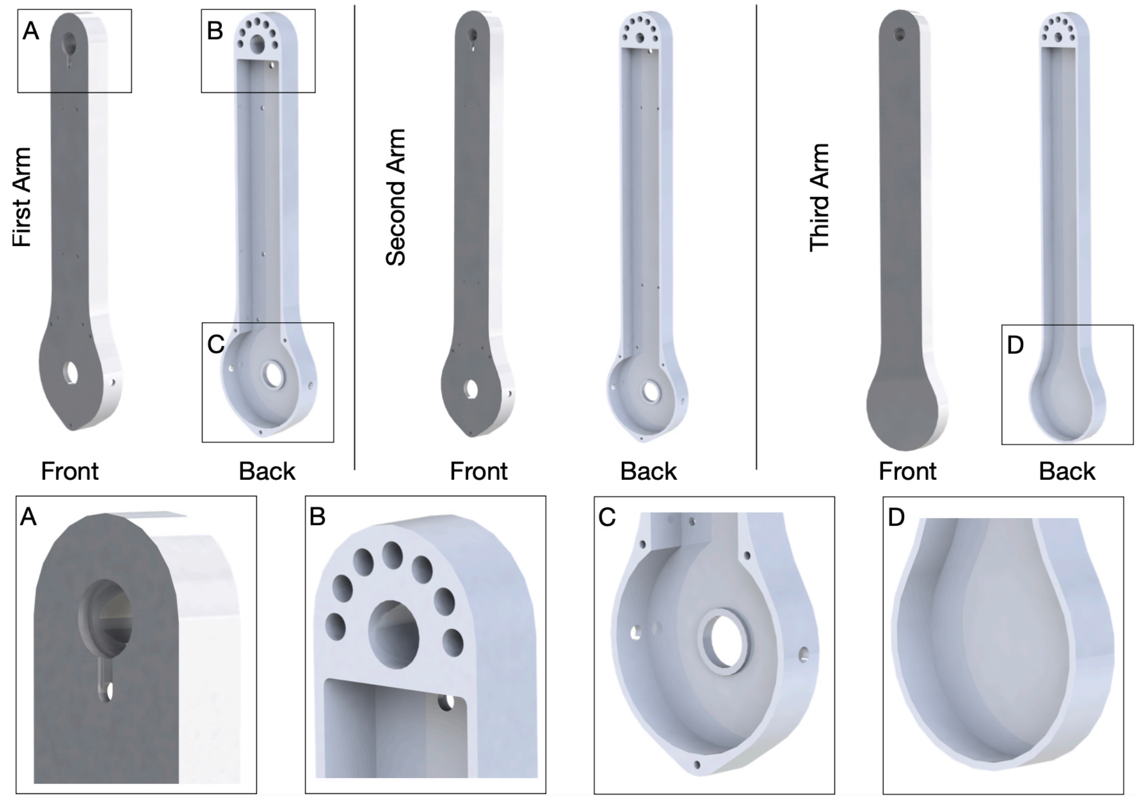

Figure 2: Design of the pendulum arm. (A) design features necessary to allow proper wiring of the slip-ring, and the hole where the pendulum shaft is installed. (B) back side of the pendulum where the shaft is installed, and a sequence of drilled holes that reduce the weight of pendulum arm. (C) place where the bearing and encoder are installed. (D) third pendulum arm without bearing hole.

Ceramic bearings are used to minimize the friction during the rotational movement. They are installed on the bearing plate shown in Fig. 1 and the pendulum arm shown in Fig. 2. The great advantage of the ceramic bearing is that it operates without lubrication, effectively decreasing the drag force caused by lubricants. Moreover, a bearing without sealing is used to further reduce the friction force, since the pendulum setup is used in a clean lab space. Thus, special care must be taken when assembling and dissembling the pendulum arm to ensure that no dust or chips enter the bearing during the process. Two bearings are used to fully support the rotational movement of the pendulum arm. Moreover, the bearings are aligned by connecting the bearing plate and pendulum arm body together. Together, they form a bearing housing where the shaft is installed. To make sure the pendulum shaft does not slide out during the rotational movement of the pendulum arm, external retaining rings are used to secure the pendulum shaft. To install the external retaining rings, grooves are machined onto the top of the second and third pendulum arm shafts, shown in Fig. 1.

B..2 Pendulum Arm Manufacturing Details

Several components of the pendulum arm are manufactured: 1) pendulum arm (Fig. 2), 2) pendulum shaft (Fig. 1), 3) bearing plate (Fig. 1), 4) protection case (Fig. 2 (E) and (F)), and 5) wire clipper (Fig. 2 (B)). In this section, we talk about the manufacturing details of those components.

The pendulum body is CNC milled from a multipurpose 6061 aluminum block with a dimension

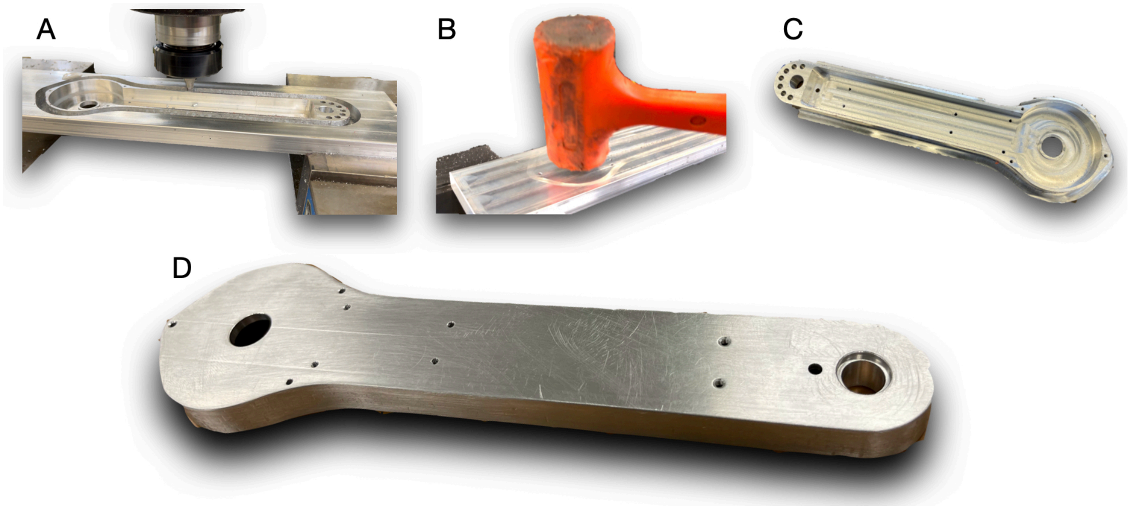

Figure 3: Overall steps needed to manufacture the pendulum arm: (A) the inside features of the pendulum arm is machined, and the outline of the pendulum arm is processed until only a thin layer of aluminium connects the pendulum arm and aluminium block. (B) and (C) the machined pendulum arm is knocked off using a robber hammer. (D) the machined pendulum arm is polished to get rid of any burr on the edge of the pendulum arm.

of 5/8" thick, 3" wide, 1 foot long. The aluminum block is first face milled on the front and back faces. Then the edge of the aluminum block is machined. This step allows to make the faces of the aluminum block parallel to each other (front and back, edge to edge). Next, the aluminum block is secured on the CNC machine, and the center of the shaft hole is used as the origin of the x, y, and z coordinates. The back side of the pendulum arm is first machined. While more material remains in the aluminum bar, the hole for the pendulum shaft is first drilled to a diameter close to the actual diameter needed. Next, the CNC mill is used to refine the shape and dimension of the hole until it is close to the desired dimensions. Finally, the reamer is used to refine the size of the hole for a smooth installation of the shaft. We choose to first machine the hole for pendulum shaft when there is more material left, since this will increase the rigidity of the aluminum bar during the drilling and milling process, resulting in a more precise hole. After manufacturing the hole for the shaft, the hole for the bearing installation is machined using similar steps, where the hole is first drilled, then milled, and finally refined using a reamer. Next, the threaded hole for connecting the bearing plate and the pendulum arm is drilled, as shown in Fig. 2 (C). After this step, the weight reducing hole shown in Fig. 2 (B) is machined. Next, the aluminum block is flipped and recenter using edge finder. Then, the hole shown in Fig. 2 (A) is milled to correct shape and depth. Next, a threaded hole is drilled to install the wire clipper and encoder, shown in Fig. 4 (B) of the paper and Fig. 2 (C). Then the pendulum arm is flipped again to the back side to machine the inner part. The inner side of the pendulum arm is milled by programming the CNC machine to achieve the desired shape, shown in Fig. 2 [1]. Once this step is finished, the outer shape of the pendulum arm is milled, shown in Fig. 3 (A). The TiAlN Coated, 2 flute, 1/4" mill diameter, 2-1/2" overall length end mill is used to mill the outer shape of the pendulum arm. At the final round of machining, a thin layer (0.5 mm) of aluminium is left to connect the pendulum arm body and the remaining of aluminum block, then a rubber hammer is used to knock out the pendulum arm, as shown in Fig. 3 (A) and (B). The

[1]The inner side corner of the manufactured pendulum arm will not be a straight corner. Instead, its shape is an arc whose radius is determined by the radius of the end mill used to machine the inner side.

resulting pendulum arm will have rough edges, shown in Fig. 3 (C). Thus, the pendulum arm is polished after the holes on the side of the pendulum arm shown in Fig. 2 (C) are drilled. The final pendulum arm is shown in Fig. 3 (D).

The similar process is used to machine the bearing plate by using a 0.16" thick, 6" x 6" multipurpose 6061 aluminum sheet. After the aluminum sheet is secured on the CNC machine, the face mill is used to get the correct height of the bearing plate. Next, the bearing hole is manufactured. Then the through holes shown in Fig. 1 are drilled. Finally, the outer shape of the bearing plate is milled until there is only a thin layer of aluminum connecting the bearing plate and aluminum sheet, and the bearing plate is knocked out using the same approach shown in Fig. 3 [2].

The material we used for the pendulum shaft is 1566 carbon steel, which balance high strength and good machinability. For the first pendulum arm, 12" long material with 1/2" diameter raw material is used, while 12" long 3/8" diameter raw material is used for the second and third shaft. To manufacture the shaft, a lathe is used. When machining the first and second arm shaft, a through hole is first drilled. Then, a shallow hole is drilled to create a step which allows proper installation of the slip-ring. This can be seen in Fig. 1. Next, the main diameter of the shaft is lathed. After the major shape of the shaft is obtained, a groove is lathed for the second and third arm to allow installing the external retaining ring. For the first arm, a thread is lathed to allow application of the axial force by screwing a nut. The groove on the bottom of the first and second shaft is milled after it is press fitted into the pendulum arm body. After the machining, all the parts are polished and cleaned to remove dust or metal chips. Finally, the protection case and wire clip is 3D printed using Polylactic Acid (PLA) material. The main functionality of the case is to protect the slip-ring inside the pendulum arm from accidental collision.

B..3 Assembly Details of Second and Third Arm

The assembly of the double pendulum is described in Sec. 3.1. For the assembly of the triple pendulum illustrated in Fig. 6 of the paper, the following steps are needed: 1) Follow all the assembly steps of the double pendulum. 2) Slide the shim (11) into the shaft of the third arm (22). Next, slide the third pendulum shaft (22) into the bearing of the second arm (12) and stop until the shim (11) contacts the inner ring of the bearing. 2) Slide the shim (16), encoder disk (17), and another shim (18) onto the third arm's shaft (22). Then, slide the assembled bearing plate onto the shaft (22). Finally, clip the external retaining ring (19) onto the shaft (22). This step is the same as mentioned before. 3) Slide the 3D printed shim (6) and encoder reader (7) into the desired place by aligning the holes. Then, install the screws (8) to secure its position. 4) Next, install the protection case by aligning the holes on the pendulum arm body, bearing plate, and protection case. Mount the screws (4) to fasten the assembly. 6) Finally, install the wire clipper (5) into the pendulum arm (15) by aligning the holes and tighten the screws (4). The above steps finish the assembly of the second and third pendulum arm, and results in the final assembly shown in Fig. 4 of the paper. The complete assembly process of the second and third arm are illustrated in Fig. 6 of the paper.

C. Pendulum Cart Details

Our multi-link pendulum uses a linear motor to provide actuation to the pendulum arm. The advantage of using the linear motor is that it does not have backslash issues, which frequently happen in the belt-drive type servo motor. This benefit allows accurate control of sensitive maneuvers, such as the swing-up of double and triple pendulums. As discussed in Sec. 3.2, to size

[2] Same as footnote 3, the straight corner in Fig. 1 will be an arc whose radius is determined by the radius of the end mill.

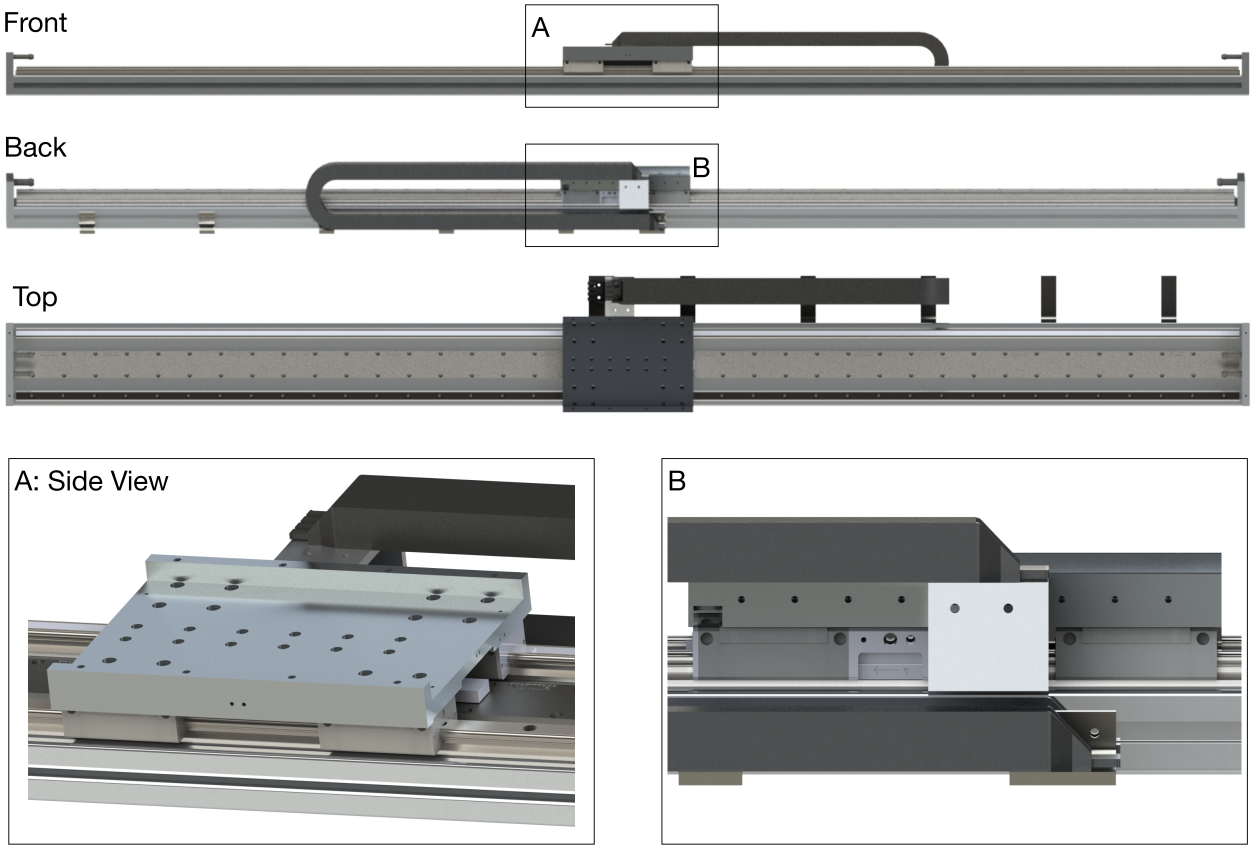

Figure 4: Overview of the linear motor that drives the pendulum cart, with the linear motor cable track and the stage with pre-drilled holes, which allow the installation of user designed parts. This linear motor stage is used to mount the aluminum plate that is used to install the bearing housing and the pendulum arm. (B) Limit switch plate installed on the back side of the linear motor stage.

the linear motor, first, the desired maximum speed and acceleration of the cart are defined using desired motion profile of the pendulum. We choose a HIWIN linear motor system LMX1K-SA12-1-2000-PGS1-V103+HS. For more details on choosing the linear motor size, we refer the readers to see Appendix A of the HIWIN linear motor system manual [2]. The overall look of the selected linear motor can be seen in Fig. 4. As Fig. 4 shows, the linear motor already comes with a cable management track and motor stage. Moreover, Fig. 4 (A) suggests the motor stage is not flat, which suggests the proper design of connection between the pendulum arm and linear motor stage is needed. Fig. 4 (B) indicates there are pre-drilled holes on the backside of the linear motor stage. Those pre-drilled holes can facilitate the installation of a switch plate. Finally, Fig. 4 also indicates that the both side of the linear motor rail has shock absorbers installed which prevents the direct collision of the motor stage and the end of linear motor rail.

The connection between the pendulum arm and the linear motor stage are shown in Fig. 7 (A) and (B) of the paper. A bearing housing is needed to provide support for the first pendulum arm shaft and secure it so that the pendulum arm can perform free swing. Moreover, an aluminum plate is machined so that the bearing housing can be connected with the linear motor stage. The details of the bearing housing can be seen in Fig. 5. As Fig. 5 shows, one thorough hole is drilled on the front and back side of the housing. This hole is used to install the bearings that mate with the first pendulum shaft. In order to maintain the relative distance of the bearings, a spacer needs to

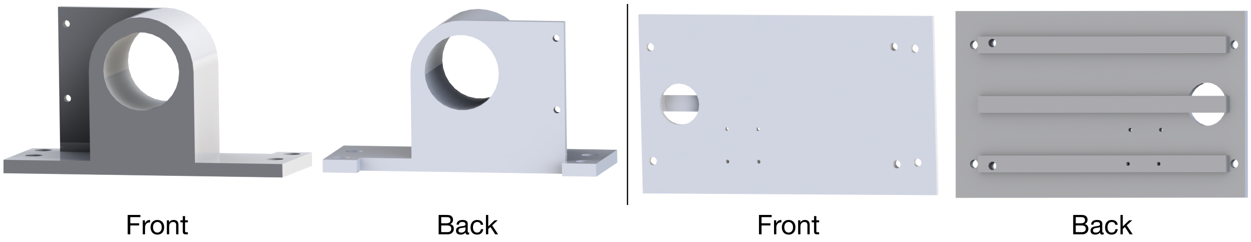

Figure 5: This figure illustrates the overall look of the bearing housing and cart plate. The main features on the bearing housing is the thorough hole that is used to install the bearing. To make the manufacturing process easier, a thorough hole is used and a spacer is later installed to maintain the distance of two bearings. As for the cart plate, it has simple shape and features. The four tiny threaded holes are used to install the cable management tool while the larger hole is milled to install the circular level indicator.

be installed. Same as the pendulum arm, the ceramic bearing is used to minimize the drag force. Four more thorough holes are been drilled on the bottom of the bearing house which is used to mount the bearing housing to the aluminum plate and linear motor stage. Two threaded holes are drilled on the side panel of the bearing house and are used to mount the encoder reader. To avoid collision of the encoder disk and bearing housing, the bottom of the bearing housing has been designed as a C shape. As for the aluminum plate, the design is straightforward as Fig. 5 and paper's Fig. 7 shows. It has four holes that is used to mate the aluminum plate with linear motor stage. The back side of the aluminum plate has multiple stripes that acts as a stiffener which is used to strengthen the plate. Four small threaded holes are drilled on the top of the aluminum plate to install 3D printed cable managing tool while one bigger hole is milled to install the circular level indicator. This circular level indicator is useful as it can help user to level the pendulum cart. The final piece we designed for the pendulum cart are the limit switch plate as it shown in paper's Fig. 7 (B). Fig. 7 (B) of the paper shows two limit switch plates installed on the back side of the linear motor stage, they are responsible to block the laser limit switch when the pendulum cart moves to the edge of the linear rail. The laser limit switch is installed on the linear motor by designing a 3D printed base as Fig. 12 (B) of the paper shows.

C..1 Pendulum Cart Manufacturing Details

The major parts that need to be manufactured in the pendulum cart are: 1) Bearing housing. 2) Pendulum cart plate. 3) Limit switch plate. 4) 3D printed cable management tool. 5) 3D printed limit switch base. This section will go through the manufacturing process of those components.

To manufacture the bearing housing, a 3" multi-purpose aluminum cube is machined. Fig. 6 shows the overall machining process.

1) To start with, the raw material is face milled on all fronts to create datum planes. Next, a shallow hole whose diameter is smaller than the diameter of the bearing is milled, and the center of the hole is set as the origin of the coordinate system. Then, the front of the bearing housing is milled to create the wanted shape. The milling continues until the end mill reaches the same height as the side panel used to mount the encoder reader. 2) Once the front shape of the bearing housing is machined, it is flipped over to manufacture the features on the backside. The aluminum cube is first milled until the end mill is at the same height as the total width of the bearing housing. Next, a through-hole is drilled whose diameter is smaller than the bearing to be installed. Then a reamer is used to refine the shape and diameter of the hole. The goal is to make the bearing hole and bearing

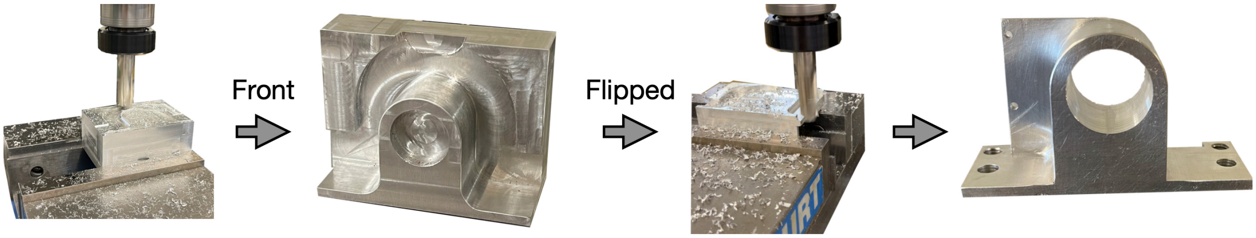

Figure 6: This figure illustrates the overall process of machining the bearing housing. To start with, the aluminum cube is face milled to create the datum face. Then the features on the front of the bearing housing is machined. Next, the aluminum cube is flipped over to machine the features on the backside of the aluminum cube and all the necessary holes needed are drill. Finally, the bearing housing is polished and sharp edges are smoothed.

have a transitional fit. Once this step is done, the milling is continued until the backside of the bearing housing is machined to desired shape. Then two threaded holes are drilled for installing the encoder reader. When drilling these two holes, the origin of the bearing hole is used as the center of the coordinated system since the relative distance between the bearing hole and the holes for installing the encoder reader is what matters. 3) Finally, the bearing housing is milled in the upright position to produce a straight corner. Once finished, it is polished using sandpaper, and the sharp edges are smoothed using a deburring wheel. It is noteworthy that this bearing housing can be 3D printed if it does not have too much load on it, for example, when only a single pendulum formulation is needed.

The manufacturing of the pendulum cart plate is easy to do. A multipurpose 6061 Aluminum with 7/16" thickness and 8" x 8" length and width is used to manufacture the pendulum cart plate. The following steps are needed to manufacture it. 1) To start with, the raw material is face milled to create a datum plane. Next, it is milled to have the same width and length as the designed cart plate. 2) Next, the front face of the cart palate is face milled, and all the holes needed are drilled. This process includes the hole used to install the circular level indicator. 3) Once the features on the front of the cart plate are manufactured, it is flipped over to mill the stiffener. Finally, the post-processing is done to polish the cart plate, and the deburring wheel is used to make the smooth edges. If the pendulum cart does not have a considerable load and does not require high-speed movement, it is also possible to 3D print the pendulum cart plate.

The left and right limit switch plates are manufactured using a multipurpose 6061 aluminum sheet with 0.016" thickness. The overall outline of the limit switch plate is first drawn on the aluminum sheet using a marker pen and ruler. Then, the holes are drilled, which are needed to install the plates to the linear motor stage. Then, a metal brake and scissors cut the aluminum sheet to the desired shape. Finally, the aluminum sheet is bent, polished, and deburred. After using the above steps, the switch plates can be produced easily. As for cable management tools and limit switch base, they are 3D printed with PLA filament.

D. System Frame Details

The linear motor selected in Sec. 3.2 is mounted on a support frame in order to minimize the unwanted vibration during the cart movement. This motivates us to design a system frame to mount the pendulum cart and provide support to it. This section introduces the design and manufacturing of the system frame we used. Moreover, we show how to install the linear motor to the system frame and provide a detailed bill of materials for reproduction.

D..1 System Frame Design Details

Fig. 7 shows the overall view of the system frame we designed. Our design is similar to the one shown in [3, 4]. To make sure the pendulum arm won't collide with the ground, four vertical aluminum extrusions are connected with the linear motor using corner concealed brackets. This lifts up the linear motor from the ground and creates space for the pendulum arm. To make sure the vertical aluminum extrusion is stable in the $x - z$ plane, the diagonal brace is used to connect the linear motor and vertical aluminum extrusion as shown in Fig. 7 (A). Moreover, four more aluminum extrusions are connected together using a machined connector plate to create a X shaped frame as shown in Fig. 7 (C). By connecting the X shaped frame and the four vertical aluminum extrusions, the movement of the frame is further constrained in the $x - z$ plane. Next, the frame's movement is constrained in the $y - z$ plane. This is achieved by connecting the vertical frames using an aluminum extrusion as shown in Fig. 7 (B) and (D). To provide more support, the vertical aluminum extrusion on the back side is further connected to another aluminum extrusion to form a triangle using T-slotted framing structural brackets, as shown in Fig. 7 (E) and (F). To provide a space to place the electrical box, the real-time system and the development system, two horizontal aluminum extrusions are used to connect the left and right vertical aluminum extrusion as shown in Fig. 7 (F). Finally, to allow easy movement of the experimental system from place to place, four wheels can be installed as an option which is shown in Fig. 7 (F). However, it is recommended that the wheels are only installed when it is necessary to move the system frames. When performing actual experiments, the wheels should be removed to avoid unwanted oscillation of the system frame.

D..2 System Frame Manufacturing Details

The manufacturing of the system frame is straightforward since most of the components can be directly purchased online. The main components that need to be manufactured are: 1) Aluminum extrusion, which has to be trimmed down to the correct length. 2) Aluminum extrusion connector plate, which needs to be machined. 3) 3D printed alignment tool, which is used to facilitate the assembly of the X shaped frame. In order to trim the aluminum extrusion to the correct length, the band saw is first used to trim the aluminum extrusion to a length that is slightly longer than the desired length. Next, the CNC mill is used to machine the cut surface of the aluminum extrusion to the correct length. Finally, the machined end is polished and unwanted burr is removed. To machine the connector plate shown in Fig. 7 (C), a 0.16" thick, 6" x 6" aluminum plate (89015K255) is machined. First, the through holes that is used to mate with the bolts and nuts are drilled. Then, a simple CNC program is written to mill the outer shape of the connector. As for the alignment tool that helps the assembly of the X frame, it is printed using PLA filament.

D..3 System Frame Assembly Details

To assemble the system frame, four major steps are needed: 1) The X shaped frames are assembled. 2) The X shaped frame is connected with the four vertical aluminum extrusions. 3) The assembled aluminum frame is connected with the aluminum extrusion in the $x - y$ plane and diagonal braces for single rails. 4) Finally, the linear motor is installed with the system frame and all the screws are fastened accordingly. The above mentioned steps are illustrated in Fig. 8.

As Fig. 8 (A) shows, to better assemble the X shaped frame, a 3D printed alignment tool $(52, 53)$ is used. The alignment tool is first assembled by inserting the 3D printed cylinder (52) into the 3D printed block (53). The 3D printed block (53) serves as a reference to make sure each aluminum

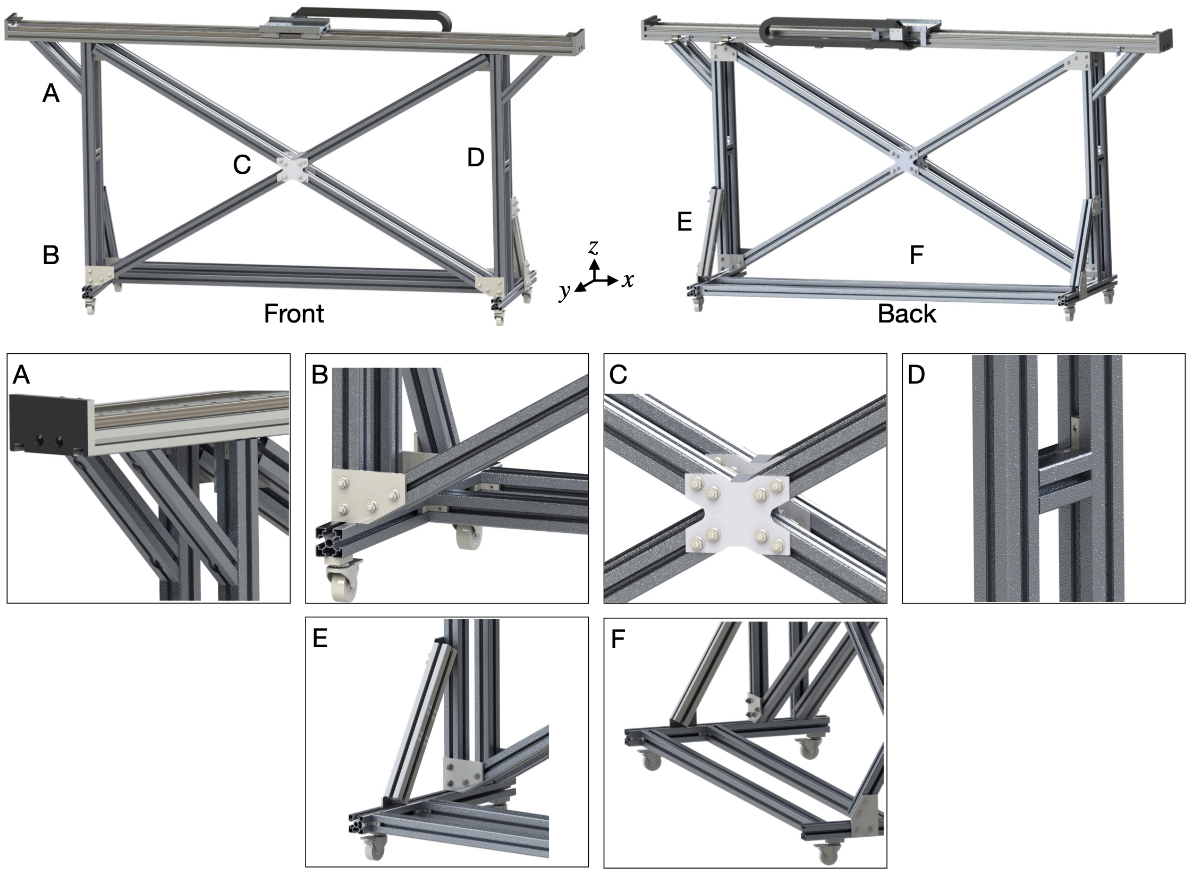

Figure 7: System frame. The linear motor is lifted up using four vertical aluminum extrusion. Different aluminum extrusions are installed to constrain the movement of system frame in the $x - z$ and $y - z$ plane. To allow easy moving of the experimental setup, four wheels can be installed to the system frame as shown in (E) and (F).

frame (50) has the correct angle to each other. This is achieved by inserting the aluminum frame (50) into the 3D printed cylinder (54), making sure it is perpendicular to the surface of 3D printed block (53). Once the correct angle between the aluminum frame (50) is achieved, the connector plate (51) is used to connect each frames using drop-in bolts and nuts (54). This finishes the step 1, assembly of the X shaped frame.

In step 2, 60 degree bracket (56) is first slid into the aluminum frame (50) as shown in Fig. 8 (B) step 1 and step 2. Then the vertical aluminum frame (55) is slid into the 60 degree bracket as shown in step 3 of Fig. 8 (B). This completes the assembly of the X shaped frame and vertical frame. To avoid the collision of the pendulum arm and 60 degree bracket (56), the top right and top left 60 degree bracket (56) is installed on the back side of front X shaped frame, as shown in Fig. 8 (B).

Fig. 8 (C) and (D) illustrates the details of step 3. First, the corner concealed bracket (60) is slid into the aluminum spacer (59). Then, this aluminum spacer is slid into the middle of front and back X shaped frame as shown in Fig. 8 (C) step 1. In step two, the wheel (62) of the system frame is assembled with the aluminum frame (61). In step 3, the 30 degree bracket (57) is first slid into the vertical aluminum frame of the back X shaped frame (55) and the bolts and nuts are slightly tightened to keep them in place. Then, step 4 is conducted in which the aluminum frame (58) is slid into the 30 degree plate (57). Next, in step 5 a 60 degree bracket (56) is slid into the aluminum

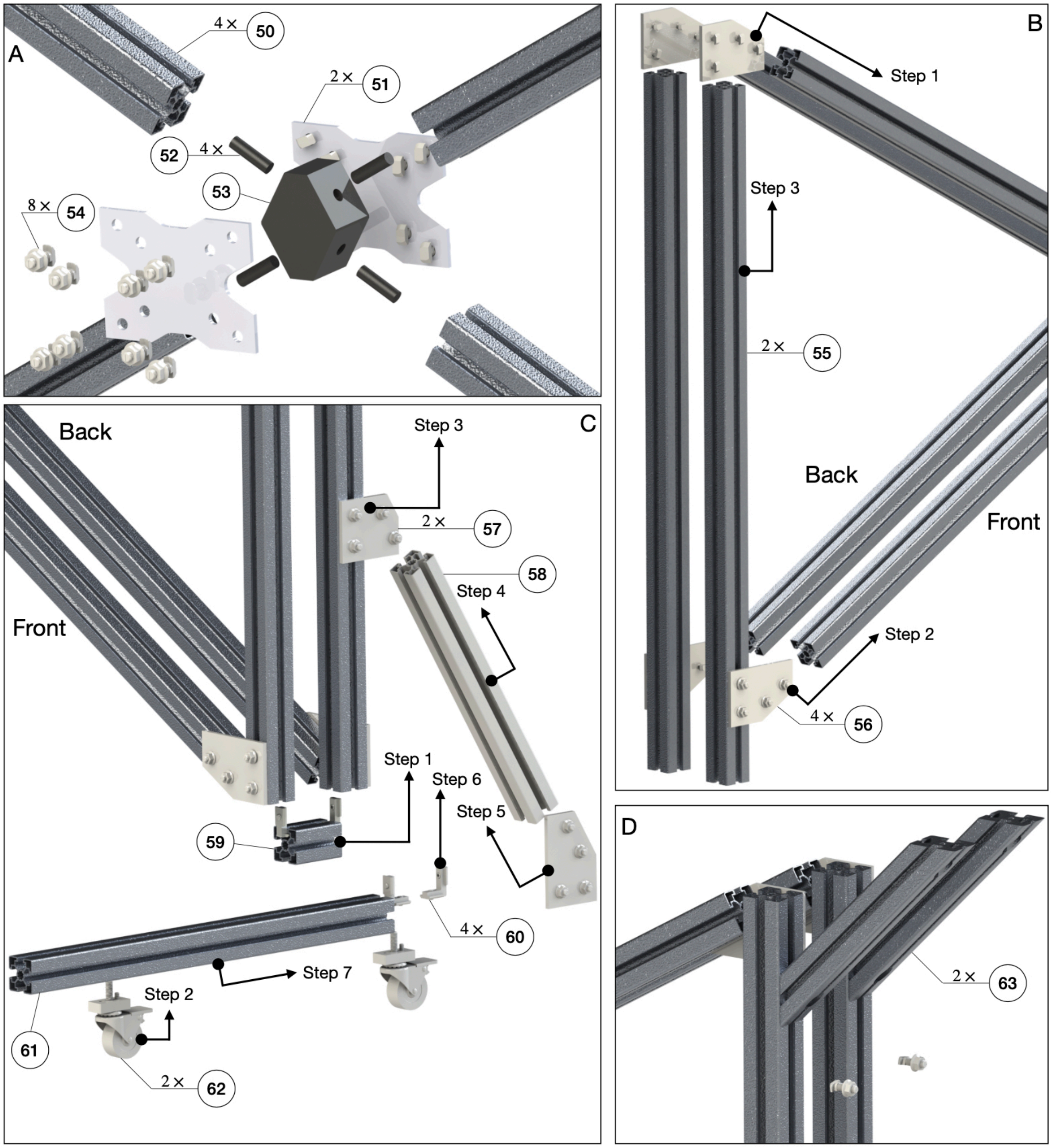

Figure 8: Assembly of the system frame. (A) Installation procedure of the X shaped frame. (B) Connection of the X shaped frame with the vertical aluminum extrusion (55). (C) Installation of the wheel to the system frame. (D) Installation of diagonal brace.

frame (58) and slightly tightened. In step 6, the corner concealed bracket (60) is installed with the vertical aluminum frame (55) and in step 7 the aluminum frame (61) is slid into the corner concealed bracket (60) and 60 degree plate (56). Next, the diagonal brace in installed into the vertical frame as shown in Fig. 8 (D), and the screws are slightly tightened to make sure the diagonal brace stays in place. The procedure to install the horizontal aluminum extrusion (64) to the aluminum extrusion with wheel (61) can be seen in Fig. 9 (A). As Fig. 9 (A) shows, the corner concealed bracket (60) is first installed into the aluminum extrusion (64) then extrusion (64) is slid into the extrusion (61). This further stabilizes the system frame and extrusion (64) can be used to place the development

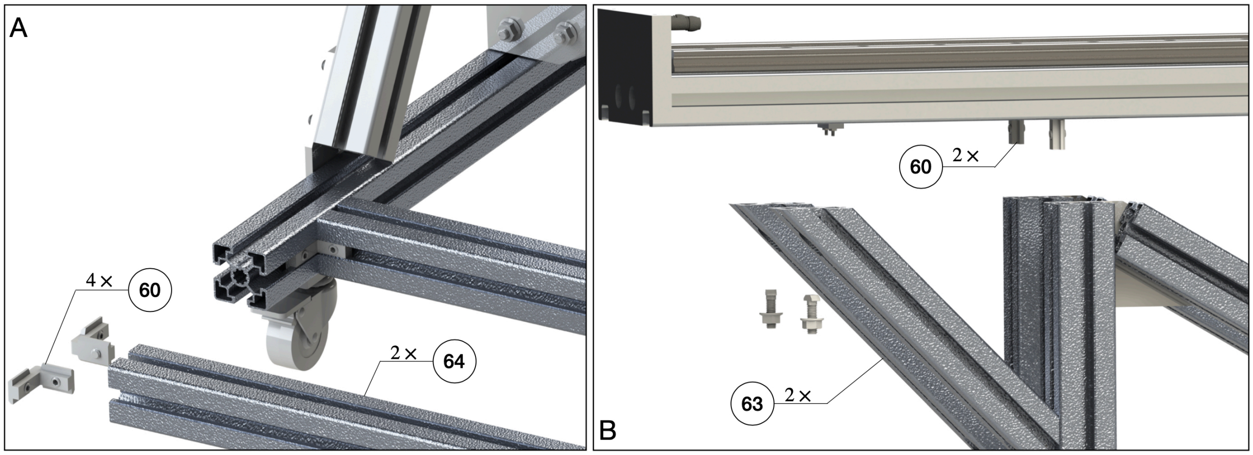

Figure 9: Assembly process of the system frame (A) and linear motor (B).

computer and electrical box, as shown in Fig. 2 of the paper. The above steps finish the assembly of the system frame. After the system frame is assembled, the linear motor (30) can be installed onto the system frame shown in Fig. 9 (B). To do so, the corner concealed bracket (60) is installed onto the aluminum base of the linear motor. Then the corner concealed bracket (60) is slid into the vertical aluminum frame (55). Next, the diagonal brace is connected with the linear motor. Finally, all the screws and nuts are tightened. Since the linear motor and aluminum frame can be heavy, all the installation process mentioned above should be finished by at least two persons.

E. Electrical System Details

E..1 Pendulum Arm Wiring Details

Fig.10 and Table. 1 show the detailed wiring specification of the pendulum arm. Fig.10 shows the first encoder is directly connected to the first differential driver while the second and third encoders are connected with the differential driver using slip-rings. Moreover, the output of the differential driver connects to the US Digital CA-C10-SH-NC 10 feet cables, which then connect to the target computer terminal board as Table. 1 suggests. It is important to point out that the US Digital CA-C10-SH-NC 10 feet cables are shielded cables. Thus, the shield also connects to the ground of the IO-392 ground pin, which helps reduce the effect of EMI noise.

E..2 Components Choice of Linear Motor's Electrical System and Wiring Details

This section will detail the functionalities and specifications of the electrical parts used to power the linear motor. A complete wiring diagram of the entire system is also provided.

The first component introduced here is the emergency stop button module. During the multi-link pendulum experiments, the linear motor may perform undesired motion due to coding error or controller failure. It is also possible that unwanted electrical shortage happens due to the improper use of the system. In those undesired situations, the user must press the emergency stop button to cut all the power supplied to the linear motor, avoiding damage to the setup and protecting the operator around the equipment. It is also necessary to have an On/Off button that controls the system's power supply. The POWERTEC 71354 magnetic switch (75) is selected as our emergency stop button to meet those requirements. This emergency stop button can be used in a single-phase

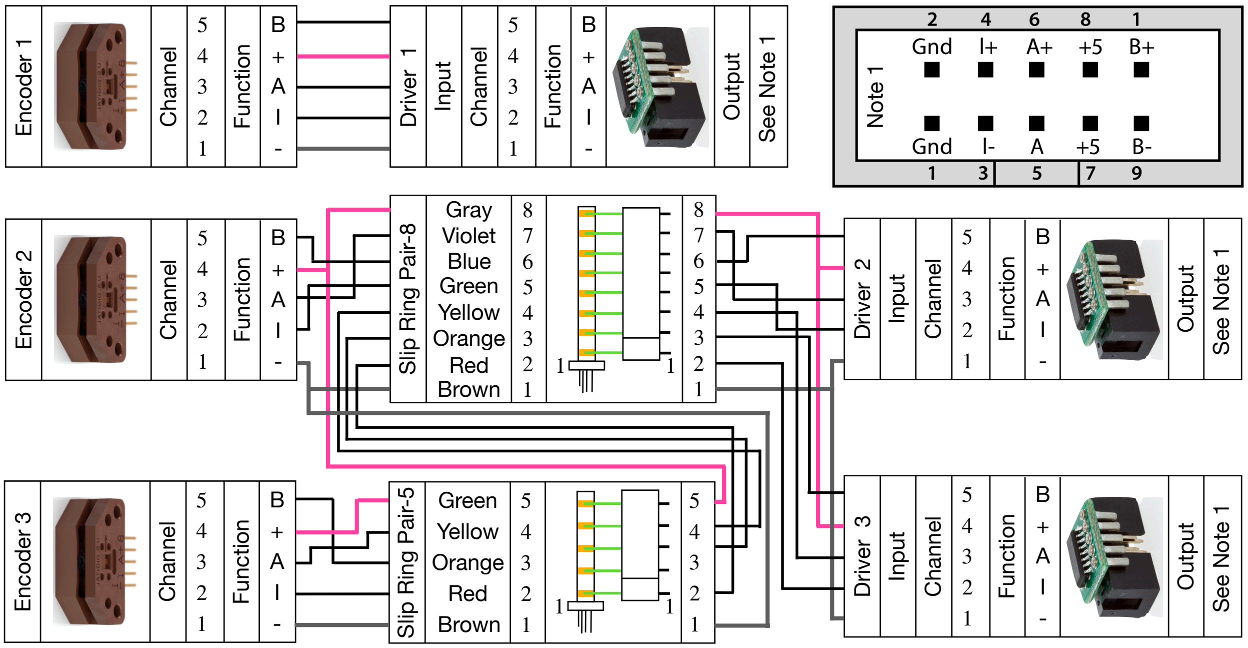

Figure 10: This figure illustrates the overall electrical wiring of the pendulum arm encoder. The first encoder is directly connected to the first differential driver. To connected the second and third encoder to the differential driver, two slip-rings (slip-ring and brush block) are used. The second and third differential driver shares the 5V and ground channel of the first slip-ring. Moreover, the first slip-ring's 5V and ground channel is connected to the second slip-ring, which then conducts electricity to the third encoder. The third encoder uses the both the first and second slip-ring to connect with the differential driver. The output of the differential driver is connected to the US Digital CA-C10-SH-NC 10 feet cables, and those cables are then connected to the IO-392 module as Table. 1 shows.

120V power line, matching how we supply the linear motor power. By checking the specification of the linear motor, we find out the peak current of the linear motor is around $12.7A_{rms}$, which is lower than the rated current of the On/Off switch ($16A_{rms}$) and emergency switch ($18A_{rms}$) installed inside the emergency stop button module. Thus, this emergency stop module is selected to control the power supply of the linear motor. The emergency stop button module should be installed somewhere that is easy to reach for the operator. This location may change based on the operator's preferred standing position. In our application, we decided to install the emergency button at the side of the system frame. Please check its user manual for details on the position holes of the module.

The next vital component is the circuit breaker (MCCB, 76). The circuit breaker has the following functionalities: 1) Circuit breaker can cut off the power supply to the linear motor when there's a shortage in the electrical system. 2) It can stop the power supply to the linear motor when it is overloaded, which avoids damage to the linear motor. 3) It can simply serve as an On/Off switch that controls the power supply to the linear motor. The first and third functions of the circuit breaker are easy to understand, and we will illustrate the second functionality more. The overloaded situation might happen when the control algorithm requires the linear motor to perform unrealistic acceleration or de-acceleration. For example, during the feedforward swing-up control of the double or triple pendulum, a feedback force is usually needed to compensate for noise and

Table 1: This table shows the pin out of IO-392 module for encoder reading and its connections with the differential driver. D1 to D3 represents the differential driver 1 to 3 shown in Fig. 10. "-x" represents the corresponding "x"-th pin of the specific electrical component. The ground of the encoder sensor is connected to "0 V" instead of "ground" as suggested by Speedgoat.

Pin	Code Module Channel	Functionality	Direction	Transceiver	Connects To
1a		Ground			
2a		Ground			
3a	1	QAD-A	IN	RS422/485(+)	D1-6
4a				RS422/485(-)	D1-5
5a	1	QAD-B	IN	RS422/485(+)	D1-10
6a				RS422/485(-)	D1-9
7a	1	QAD-C/Index	IN	RS422/485(+)	D1-4
8a				RS422/485(-)	D1-3
9a	2	QAD-A	IN	RS422/485(+)	D2-6
10a				RS422/485(-)	D2-5
11a	2	QAD-B	IN	RS422/485(+)	D2-10
12a				RS422/485(-)	D2-9
13a	2	QAD-C/Index	IN	RS422/485(+)	D2-4
14a				RS422/485(-)	D2-3
15a		0 V			D1-2, D2-2, D3-2, Differential Cable Shield
16a		5 V			D1-7, D2-7, D3-7
17a		Ground			
1b		0 V			
2b		5 V			
3b	3	QAD-A	IN	RS422/485(+)	D3-6
4b				RS422/485(-)	D3-5
5b	3	QAD-B	IN	RS422/485(+)	D3-10
6b				RS422/485(-)	D3-9
7b	3	QAD-C/Index	IN	RS422/485(+)	D3-4
8b				RS422/485(-)	D3-3
9b	4	QAD-A	IN	RS422/485(+)	HIWIN D1-CN2-16
10b				RS422/485(-)	HIWIN D1-CN2-17
11b	4	QAD-B	IN	RS422/485(+)	HIWIN D1-CN2-18
12b				RS422/485(-)	HIWIN D1-CN2-19
13b	4	QAD-C/Index	IN	RS422/485(+)	HIWIN D1-CN2-20
14b				RS422/485(-)	HIWIN D1-CN2-21
15b	1	Interrupt Input	IN	RS422/485(+)	
16b				RS422/485(-)	
17b		Ground			

model uncertainty. It has been shown [5, 6] that sometimes the LQR gain calculated to stabilize the feedforward trajectory is near singular. This indicates a considerable control input needs to be applied to the system to compensate for a slight deviation. Suppose the feedback control force is not thresholded. In that case, the controller may require the linear motor to apply unrealistic force to the pendulum arm, causing a massive amount of currents supplied to the linear motor resulting in overload. Another case where overload might happen, although unlikely in our case, is that the linear motor gets stuck mechanically. In this situation, the motor drive will try to apply more current to the linear motor to make it move, eventually causing over current. Thus, having a circuit breaker is crucial in controlling the linear motor for safety reasons. The Eaton miniature

circuit breaker FAZ-B3-2-NA (76) is used in our application. According to the user's manual, its rated current is $3A$, with the maximum allowed AC voltage being $277V$. The tripping current is 3 to 5 times the rated current, with stripping time around 0.005 to 2 seconds. The resulting tripping current ranges from 9 to $15A$ and covers the peak current of the linear motor, which is $12.7A$. Thus, using this circuit breaker can help us avoid linear motor damage when the current supplied to the linear motor is larger than the allowed peak current. Moreover, the selected circuit breaker uses a trip-free design and can not be defeated by manually holding the handles at the "On" position. Another plus is that the user can easily install it on the DIN rail. For more specifications on the circuit breaker, please check its user manual.

The magnetic contactor (77) is needed to start and turn off the linear motor. The magnetic contactor is a middle path between the power source and the linear motor. It helps avoid the direct connection of the power source to the linear motor, which helps prevent the sudden change of state of the linear motor. Combined with a circuit breaker, they can be thought of as a motor starter. The WEG Electric CWC016-00-22V18 (77) is used as a magnetic contactor in our design, allowing easy installation on the DIN rail. This contactor has two normally open and two normally closed contacts rated $16A$ current input under $60Hz$ $120VAC$. This specification fits our needs since the power output in the US is $120VAC$ at $60Hz$, and the maximum current needed by the linear motor is below the rated current of the contactor. By checking the user manual of this contactor, a "locking" mechanism of the magnetic contactor is achieved by using a momentary switch. The LCLICTOP AC 660V Red and Green Momentary Switch (86) is used to accomplish this. It can operate up to 660 VAC, and the current rating is 10 A. Fig. 11 shows the overall wiring of the magnetic contactor and momentary switch.

It is recommended to install a line reactor on the motor drive's input side to protect it from voltage spikes, surges, and transients. The LR-11P0-1PH (79) is used to achieve this. This line reactor has a rated current of $16A$ at $120VAC$. Thus, it satisfies our usage scenario in a single-phase case.

The linear motor drive (80) controls the movement of the linear motor by providing a high-frequency signal with sharp pulse edges. Those sharp edges of the signal can introduce unwanted ground current to the entire electrical system. The ground current can even cause interference between the different electrical components. This kind of interference is not wanted, especially when an accurate reading of the pendulum arm rotational angle is needed. The interference between the linear motor and encoder can cause additional unwanted pulse width signals to appear in the encoder's A, B, and Index channels, which might result in the wrong sensor reading. This is why the differential driver is used to transfer the single-ended encoder signal into the differential encoder signal to reduce the effect of electrical noise. Except for electrical interference, EMI can also cause electrical overstress (EOS) to sensitive components [7].

A multi-stage AC/DC EMI filter SCHAFFNER FN-2090-10-06 (78) is used according to the D1 drive's user manual to reduce the ground current in the electrical system and protect all other appliances that share the same ground, such as the host and target computers and monitors. The HIWIN MF-40-S edge filter (81) is used to eliminate the noise effect further. The edge filter helps reduce the EMI noise on the output end of the motor drive. Two D1-EMC1 EMI cores (87) can also suppress the EMI noise. One EMI core is installed on the linear motor's power input cable near the motor drive end, while the other is installed on the motor cable near the linear motor's stage end. Besides using the approaches mentioned above to reduce the EMI interference, another easy way to reduce EMI interference is proper cable management. For example, make the power cable of the linear motor stay far away from the sensor cable.

A regenerative resistor is usually needed to avoid the regenerative energy of the linear motor becoming too large and causing overvoltage to the motor drive. The motion profile of the linear

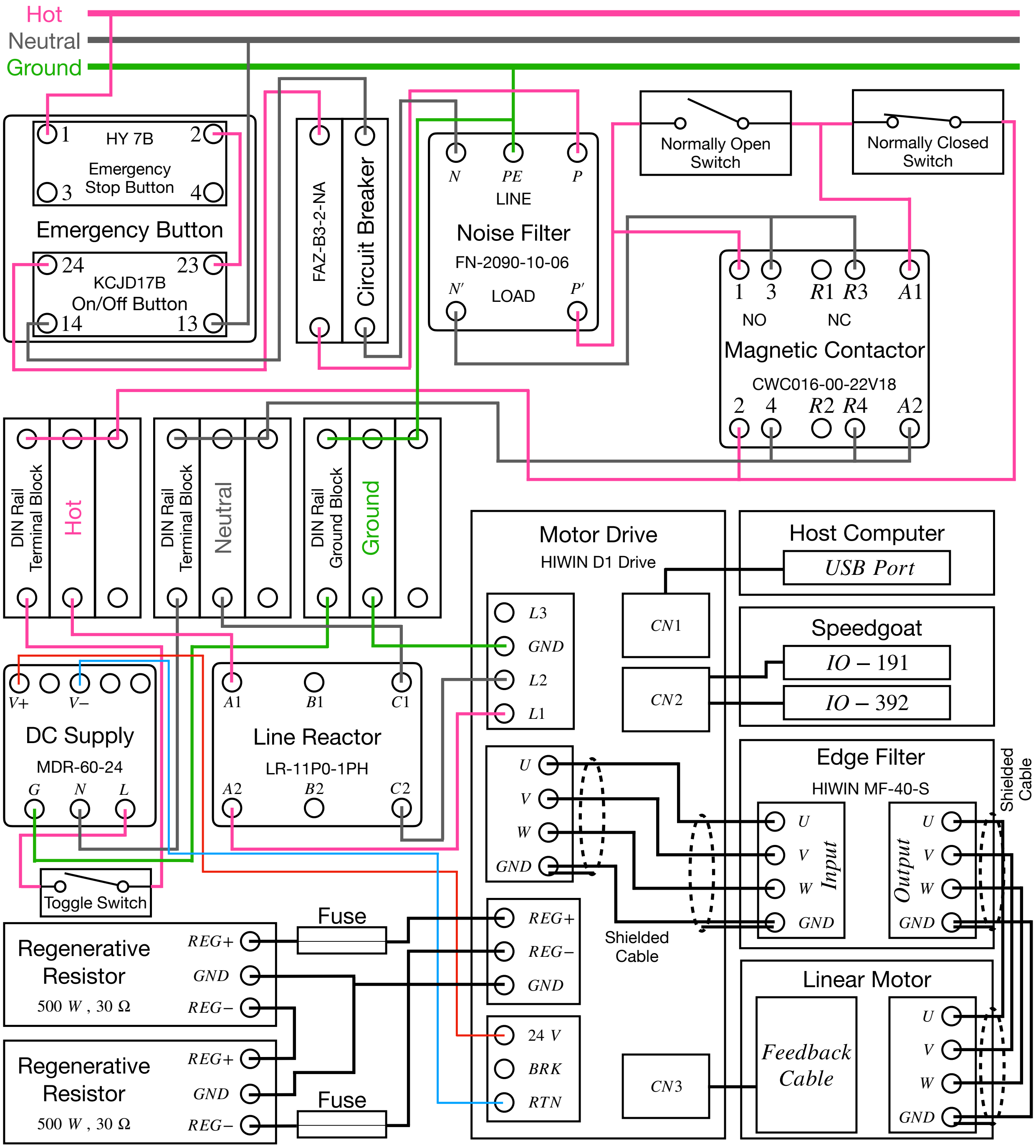

Figure 11: This figure illustrates the overall electrical components needed to provide power supply to the linear motor. An electrical box is used to put all the components together.

motor and the weight of moving mass need to be known to pick a regenerative resistor. Then the designer can use the calculation shown in the HIWIN D1 drive's user manual (see section 8.2 of the user manual [8]) to guide the selection of the regenerative resistor. The moving mass of the linear motor is around $5kg$, and the weight of the pendulum arm and cart plate is around $0.5kg$ in our design, resulting in a total moving mass of $M_t = 5.5kg$. Then, assume the linear motor is

Table 2: This table shows the pin out of IO-191 module and its detailed connection with HIWIN D1 drive's control signal channel (CN2).

Pin	Functionality	Direction	Connects To	Pin	Functionality	Direction	Connects To
1a	Analog Input 01	In		1b	Digital I/O 01	Out	HIWIN D1-CN2-3
2a	Analog Input 02	In		2b	Digital I/O 02	Out	HIWIN D1-CN2-4
3a	Analog Input 03	In		3b	Digital I/O 03	Out	HIWIN D1-CN2-5
4a	Analog Input 04	In		4b	Digital I/O 04	Out	HIWIN D1-CN2-6
5a	Analog Input 05	In		5b	Digital I/O 05	Out	HIWIN D1-CN2-7
6a	Analog Input 06	In		6b	Digital I/O 06	Out	HIWIN D1-CN2-12
7a	Analog Input 07	In		7b	Digital I/O 07	Out	
8a	Analog Input 08	In		8b	Digital I/O 08	Out	
9a	Analog Output 01	Out	HIWIN D1-CN2-24	9b	Digital I/O 09	In	HIWIN D1-CN2-13
10a	Analog Output 02	Out		10b	Digital I/O 10	In	HIWIN D1-CN2-14
11a	Analog Output 03	Out		11b	Digital I/O 11	In	HIWIN D1-CN2-15
12a	Analog Output 04	Out		12b	Digital I/O 12	In	
13a	Analog Ground	In	HIWIN D1-CN2-25	13b	Digital I/O 13	In	NC
14a	Analog Ground	In		14b	Digital I/O 14	In	
15a	0 V	NONE		15b	Digital I/O 15	In	
16a	5 V	Out		16b	Digital I/O 16	In	
17a	Analog Ground	In		17b	Digital Ground	In	HIWIN D1-CN2-1<br>HIWIN D1-CN2-2

Table 3: This table shows the pinout of the limit switch cable and its connection. The HIWIN D1 drive's CN2 channel is used to provide DC current to the limit switch. The limit switch's signal is then sent to the motor drive for the out of range protection.

D-Sub 9-Pin (Female)	Color	Function	Connects To
1	Brown	V+	HIWIN D1-CN2-22
2	Green	L-1	NC
3	Yellow	Out-1	HIWIN D1-CN2-11
4	Violet	L2	NC
5	Gray	Out-2	HIWIN D1-CN2-10
9	Blue	V-	HIWIN D1-CN2-23

moving at $V_1 = 4m/s$ and needs to deaccelerate to $V_2 = 0m/s$ with $a = 40m/s^2$. Using the above information and following the calculation shown in the user manual, we find there's no need to use a regenerative resistor since the energy return to the motor drive is smaller than the D1 drive amplifier's absorption capacity. However, we still decided to pick two 500W 30Ω regenerative resistors (82) and connect them in serial in case the returned energy is larger than the energy storage capacity.

The proper operation of the linear drive requires the supply of $24V$ DC voltage. Thus, the MDR-60-24 AC to DC industrial DIN rail power supply (83) is used to output $24V$ DC at $2.5A$. A toggle switch (85) is used to control the On/Off state of the DC power supply to the linear motor drive. The Hammond CSKO242410 enclosure (84) is used to install electrical components. A Hammond CSFC2424 flush cover (88) is cut down to be used as an electrical panel that mounts some of the components mentioned above. Two DIN rails (89) are installed inside the electrical enclosure to facilitate the installation of different electrical components. This completes the selection process of all the electrical components related to the linear motor. Fig. 11 shows their wiring details.

As Fig. 11 shows, the emergency stop button is first connected to the single-phase power supply. Two 14 AWG power cord (90) is used as the emergency stop button's power input/output cable. Next, the output of the emergency stop button is connected with the circuit breaker, which serves

as over-current protection. Then, the noise filter is connected to the circuit breaker. The connection wire used for the hot and neutral line is 12 AWG wire (91), while the connection wire for the ground is 10 AWG wire (92). The output of the noise filter connects to the magnetic contactor. The magnetic contactor is wired to self-lock when the normally open momentary switch is pressed. The output of the magnetic contactor is wired to the DIN rail terminal block (93, 94) to facilitate cable management better. Then, the DC supply and line reactor input is connected to the DIN rail terminal block. The output of the DC supply is $24V$ DC, and it is used to provide power to the motor drive. A toggle switch is used to make the supply of DC voltage controllable. Then, the output of the DC supply is connected to the Wago 721-103/026-000 connector (95). The output of the line reactor is connected to the AC power port of the motor drive using a single-phase connection formulation. The connector used is Wago 721-204/026-000 (96). The U, V, W, and GND channel is connected to the corresponding channel on the edge filter using a shielded cable to reduce the EMI noise. The connector used on the motor drive is Wago 721-104/026-000 (97). The connector used in the edge filter's input port is Phoenix Contact 1718517 (98). The output of the edge filter then connects to the linear motor's power cable, which is a shielded cable as well. The output connector of the edge filter is Phoenix Contact 1718504 (99). Two EMI cores (87) are installed to reduce the EMI noise further. One is near the motor connection end on the motor drive and the other near the motor stage end of the linear motor. Finally, two regenerative resistors are connected to the regenerative resistor port of the motor drive in serial. The connector used for the regenerative resistor port of the motor drive is Wago 723-603 (100). The reader can choose to install two fuses as well for over-current protection. This completes the power supply to the linear motor. A D1-DNT07A USB232 to RJ11 adapter cable (101) is used to set up the motor drive parameters. The linear motor's feedback cable is connected to the CN3 of the motor drive to let the motor drive read the encoder and hall sensor signal from the linear motor. The encoder signal of the linear motor, along with other I/O signals of the motor drive, is connected to the Speedgoat's terminal block. This is achieved by making a custom cable using hook-up wires. This custom cable consists of 24 AWG hook up wires (102), cable shielding sleeve (103), cable sleeve (104), 3M 10126-3000 connector (105) and its cover 3M 10326-52F0-008 (106). Table. 1 and Table. 2 show the connections between the CN2 of the motor drive and the Speedgoat real-time system. Section 3 of the motor drive's user manual contains the connection details of the motor drive and its pinout of control signals (CN2). The interested reader should reference the user manual if future system modifications are needed. The final piece to mention is the limit switches we used in the linear motor for out-of-range protection. A HIWIN 3-meter limit switch extension cable is used (107) to connect the limit switch and motor drive. Table. 3 shows the pinout of the limit switch cable and its corresponding connection to the motor drive. Zip tie and wire clips are used to make proper cable management by securing the position of all the cables/wires we used.

F. Software Setup Details

F..1 Software Setup of Motor Drive Details

The HIWIN D1 drive's corresponding setup software is Lightning, and the reader can find a detailed introduction in section 4 and 5 of the HIWIN D1 drive's user manual [8]. The version of the software we are using is V-0.1999, while the latest version is V-0.20 at the time of writing this paper. First, we need to download the Lightning software to the host computer to set it up. Next, the CN1 port of the D1 drive is connected to the USB port of the host computer using a communication cable (101). Once finished, the D1 drive is powered up, and the Lightning software

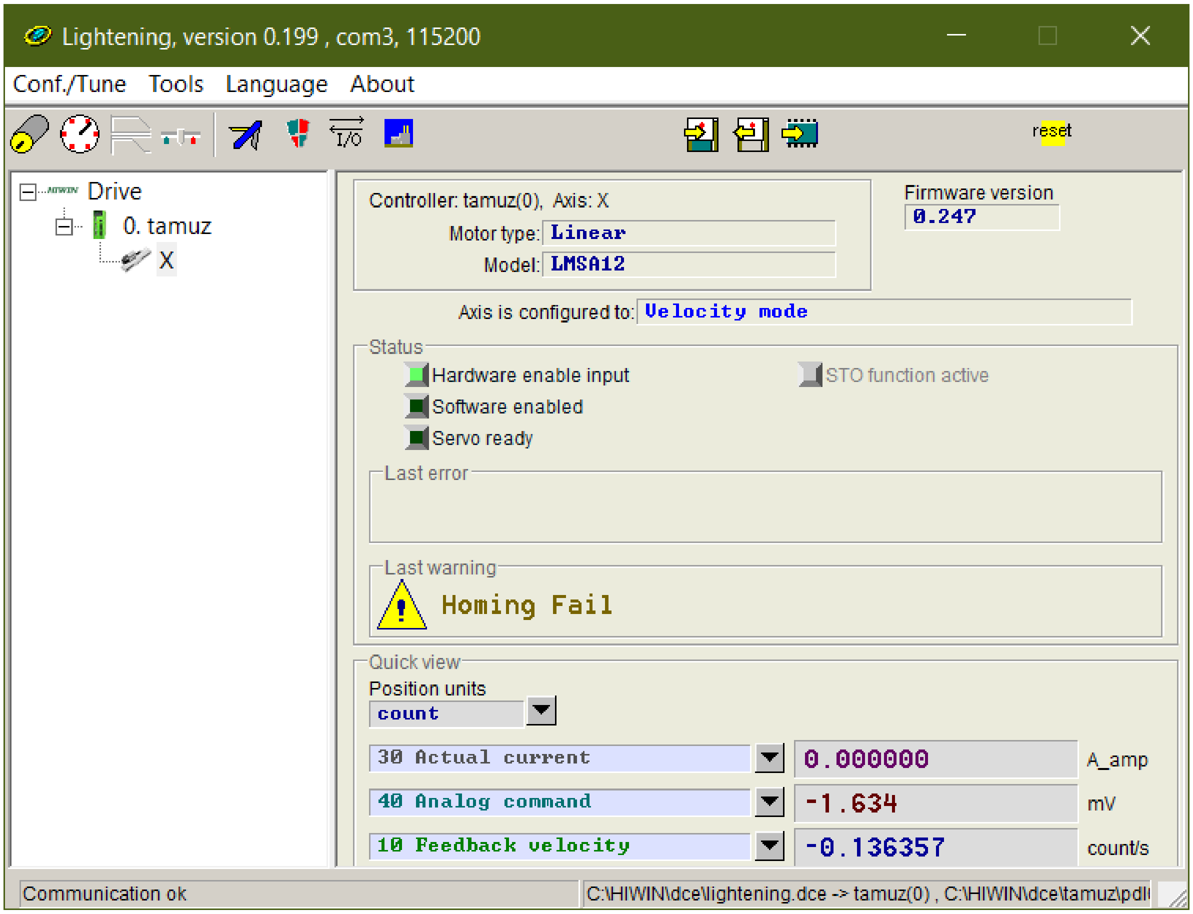

Figure 12: This figure illustrates the overall look of the Lighting software.

is executed. Then, in the software's menu, select "Tools->Communication setup," then configure the "BPS" drop-down list to 115200 and select the USB port that is used to connect the communication cable in the drop-down list. Finally, hit the "Apply" button, and the host computer and motor drive connection are complete. The above steps are needed to set up the necessary parameters of the motor drive. Fig. 12 shows the overall look of the Lighting software after connecting to the host computer.

To select the correct linear motor type, motor sensor, and operating mode, we need to select "Conf./Tune->Configuration Center," which will open a new window that allows the configuration of the motor parameters and sensors. We need to first click on the "Motor" tab to select the corresponding linear motor we are using. The LMSA12 linear motor is selected in the menu box on the left. This will bring out the pre-stored motor parameters. If the linear motor from a different brand is used, the user must enter all those parameters manually. In the "Moving mass" blank, one can enter the mass of the linear motor stage. In our case, it is $5kg$. Fig. 13 illustrates this process. After selecting the correct linear motor, we need to click on the encoder tab to set up the encoder reading options, as Fig. 14 shows.

In Fig. 14, the "Digital $1\mu m$.enc" is selected. The "Encoder output" panel in Fig. 14 allows us to set up the encoder resolution output of the motor drive. Users can scale the encoder output of the motor drive to allow the successful reading of the encoder signal if another custom real-time system is used that can not read an encoder with fine resolution. This completes the setup of

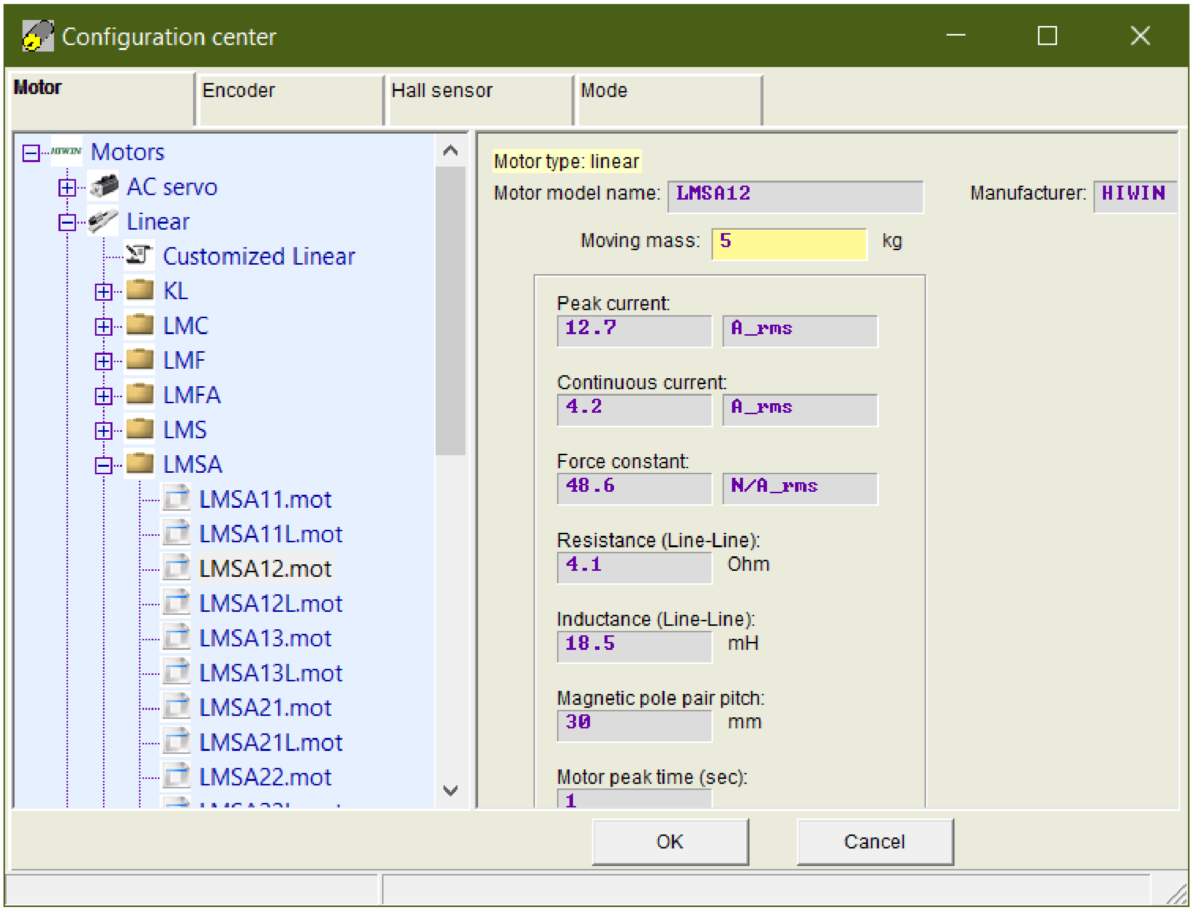

Figure 13: This figure illustrates the configuration of the motor parameters.

the linear motor encoder. Fig. 15 shows the configuration of the hall sensor. This is achieved by clicking the "Hall Sensor" panel, and select the type of the Hall sensor as "Digital Hall sensor," and clicking on the "Enable hall phase check" option. The final parameter that needs to be configured in "Configuration Center" is the operating mode of the linear motor.

There are multiple operating modes of the linear motor. The first one is the position mode. The position mode takes the user desired position as a reference and controls the linear motor to stay at the reference position. The second mode is velocity mode. In velocity mode, the velocity of the linear motor is controlled. The user usually provides an analog signal to the motor drive, and scaling is used to transfer this analog signal to the desired velocity of the linear motor. Next, the motor drive will control the linear motor to achieve the desired speed. The third mode is force/torque mode. In the force/torque mode, the user again provides an analog voltage signal to the motor drive. Then, this voltage signal is scaled to the corresponding force/torque generated by the motor. The force/torque mode directly controls the acceleration of the motor. The final mode available is the stand-alone mode which means the linear motor can only be controlled using the Lightning software instead of the user-provided signal. In this paper, we follow the work of [5, 6] and use the velocity mode as our control method of the pendulum cart. As Sec. I. shows, this results in a nicer equation of motion for the pendulum on the cart system.

The HIWIN D1 drive supports two operating modes to be selected. The first one is set to stand-alone mode as our default operating mode. This helps avoid the unwanted movement of

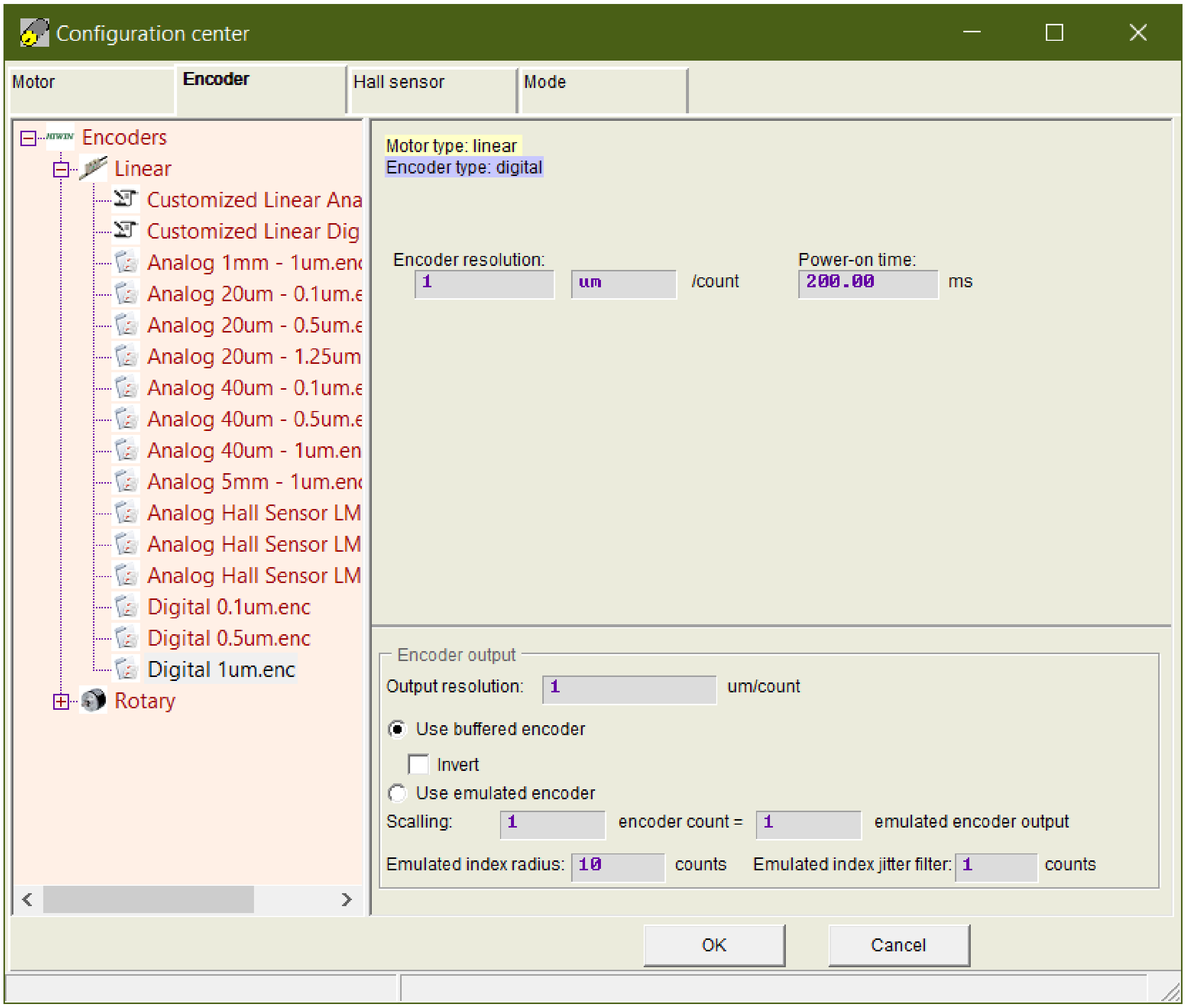

Figure 14: This figure illustrates the configuration of the linear motor's encoder.

the linear motor when the analog signal is accidentally drifting. The secondary mode is chosen to be velocity mode, which allows us to control the velocity of the linear motor given analog input. Fig. 16 shows the configuration of the linear motor's operating mode. During the pendulum experiments, we usually desired a speed range of $\pm 3m/s$ for the linear motor. Given the output range of the analog signal is $\pm 10V$, the scaling we used is $0.3m/s = 1V$. Moreover, we set a dead band of $10mV$. This is necessary since the analog signal is always going to be noisy. Thus, it is desired to put a dead band and filter out low magnitude noise to prevent the unwanted motion of the linear motor. The dead band we used filters out any velocity command lower than $3mm/s$.

The dead band should not be too large, or it will affect the performance of the pendulum stabilization task, especially during the stabilization of the multi-link pendulum, where the fine movement of the pendulum cart is needed. The maximum value of the dead band that the user can select is determined by the parameters of the pendulum arm, sampling rate, scaling factor, etc. No analytical analysis is given here for adequately selecting the dead band of the analog signal. Still, a general rule to keep in mind is that the dead band should be as small as possible while eliminating most of the noise in the analog signal. The above process summarizes the configuration of the linear motor using Lightning software.

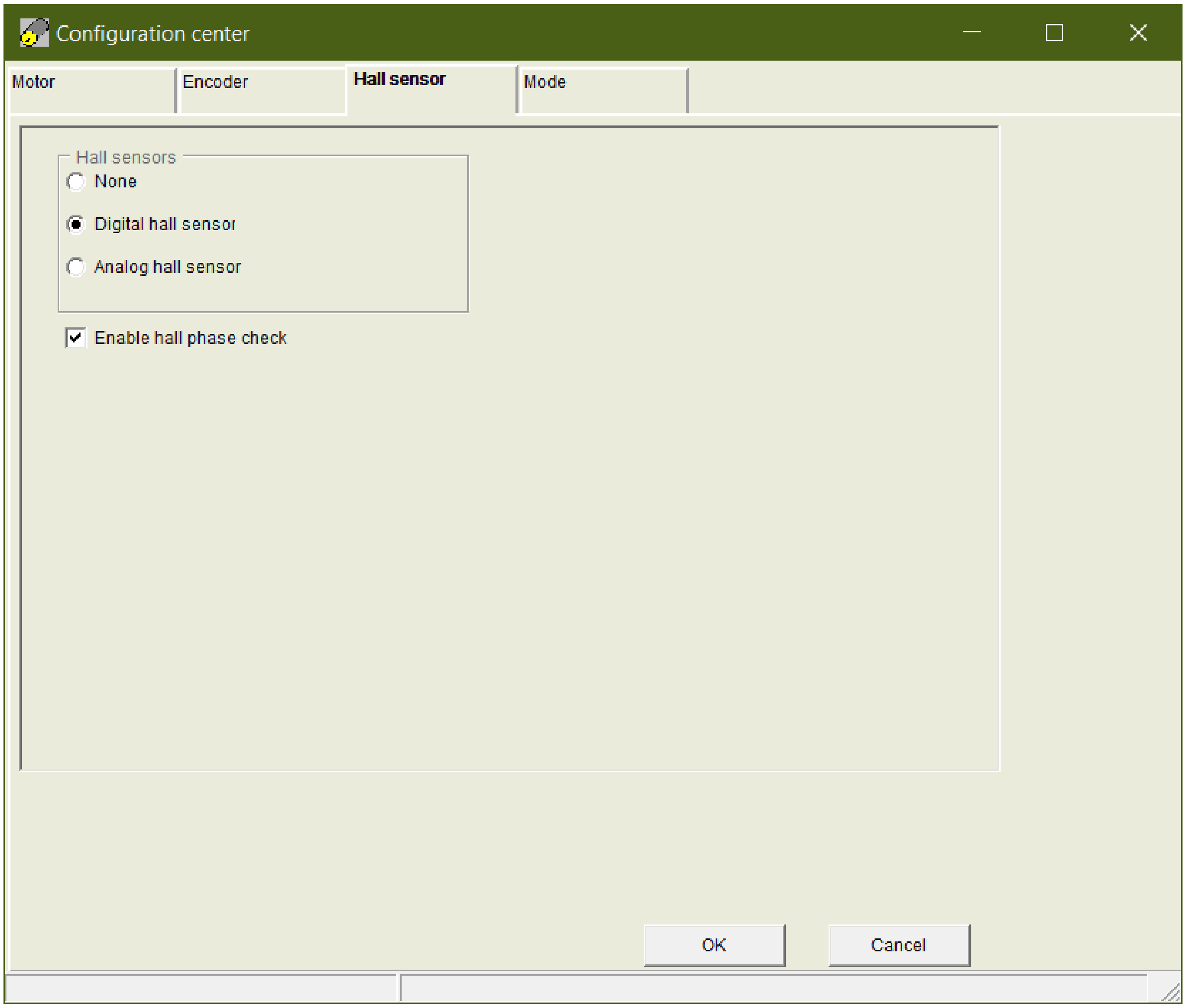

Figure 15: This figure illustrates the configuration of the linear motor's hall sensor.

A digital input signal is needed to switch between the primary and secondary operation mode, with its high and low states corresponding to different operating modes. To set up the I/O functionality of the motor drive, the user needs to click the "Conf./Tune->I/O Center" to open the I/O setting window. Fig. 17 shows the overall look of the configured I/O ports. In the "Inputs" tab, the I1 port of the CN2 channel (HIWIN D1-CN2-3) is configured as the hardware enable port. When the input of this channel is high, the linear motor will be hardware-enabled, and the user can achieve control of the linear motor. The I2 port of the drive (HIWIN D1-CN2-4) is configured to decide the operating mode of the linear motor. When a high signal is detected, the drive will enter the secondary mode, which is velocity mode in our case. The I3 port (HIWIN D1-CN2-5) is configured to control the homing function of the linear motor. The homing process will first drive the linear motor to the left and right limit switches. Then it will use the location information of the limit switch to find the center location between them and drive the linear motor to it. The homing function can only be activated when the motor drive is in stand-alone mode with high input received in the I3 port. Next, the I9 and I10 port (HIWIN D1-CN2-10 and 11) are configured as the limit switch signal input channel. Table. 3 shows the detailed connection between the limit switch and CN2 channel. The above configuration helps the motor drive determine whether the

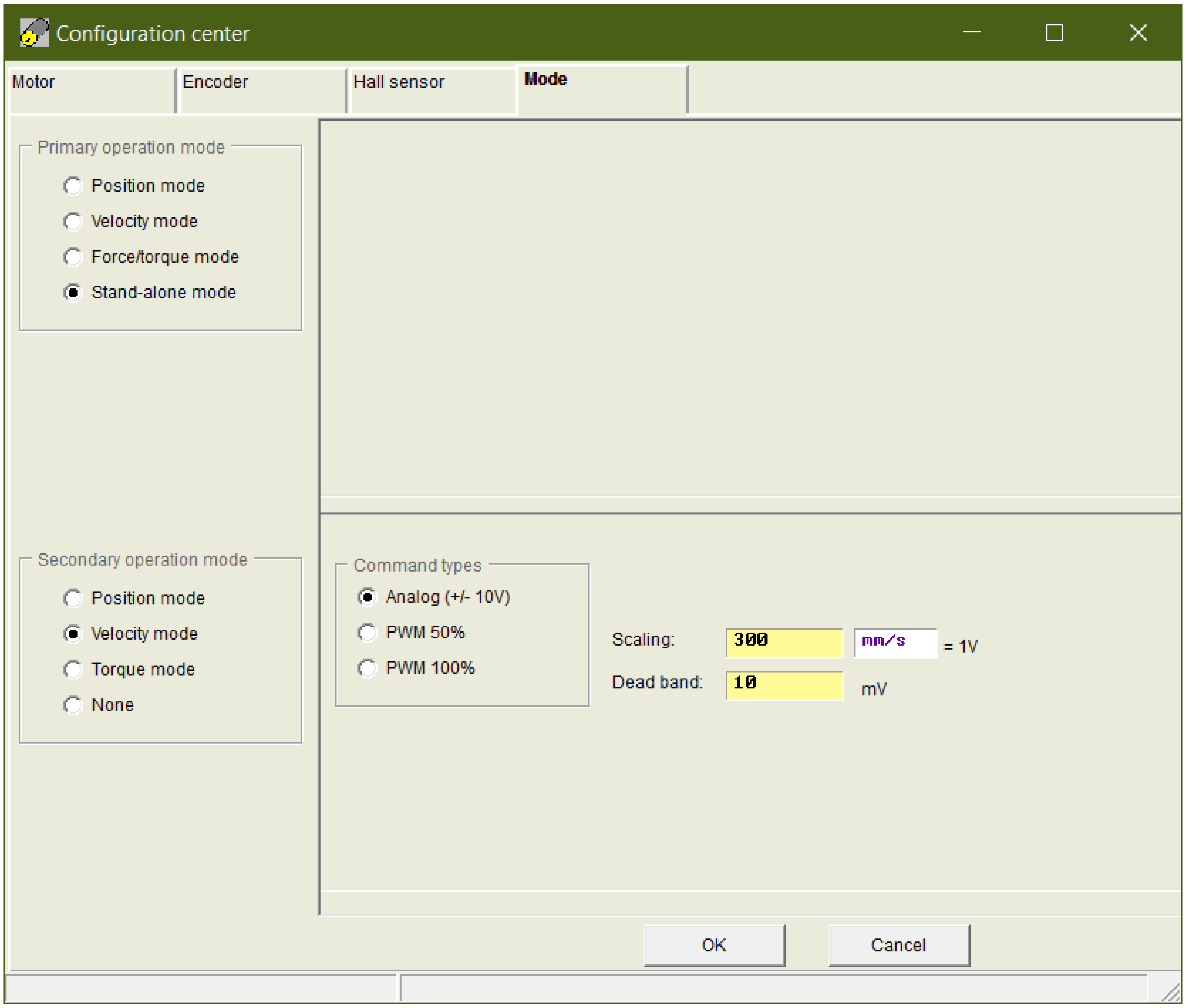

Figure 16: This figure illustrates the selection of the linear motor's operating mode.

linear motor reaches the limit switch. If so, the linear motor's movement beyond the limit switch is restricted. However, it will not deactivate the linear motor and stop its power supply. When an opposite-moving signal is supplied to the linear motor, it can still move in the opposite direction. This behavior is not desired, though. Once the linear motor hits the limit switches, a program should cut off all linear motor's power supply. The limit switch signal is supplied to the Speedgoat system to achieve this functionality. The output ports of the CN2 channel, HIWIN D1-CN2-13 and 14, are configured to be the limit switch output signal. Then, as Table. 2 shows, the output of the CN2 channel is connected to the Speedgoat system. Next, the limit switch signal is read in the Simulink model, and once it is high, meaning the linear motor hits the limit switch, the hardware enable is deactivated. This will cut off all the power supply to the linear motor. Moreover, it will also stop the Simulink model from executing, thus stopping the experiment. The above process completes the I/O configuration of the linear motor drive.

Due to the EMI noise in the electrical system, the analog signal that controls the linear motor will contain some level of DC bias. In velocity mode, this DC bias constantly moves the pendulum cart to one side of the linear rail and thus should be avoided. By selecting "Conf./Tune->Performance Center" to enter the performance center, the "Advanced Gain" button can be pressed, which will

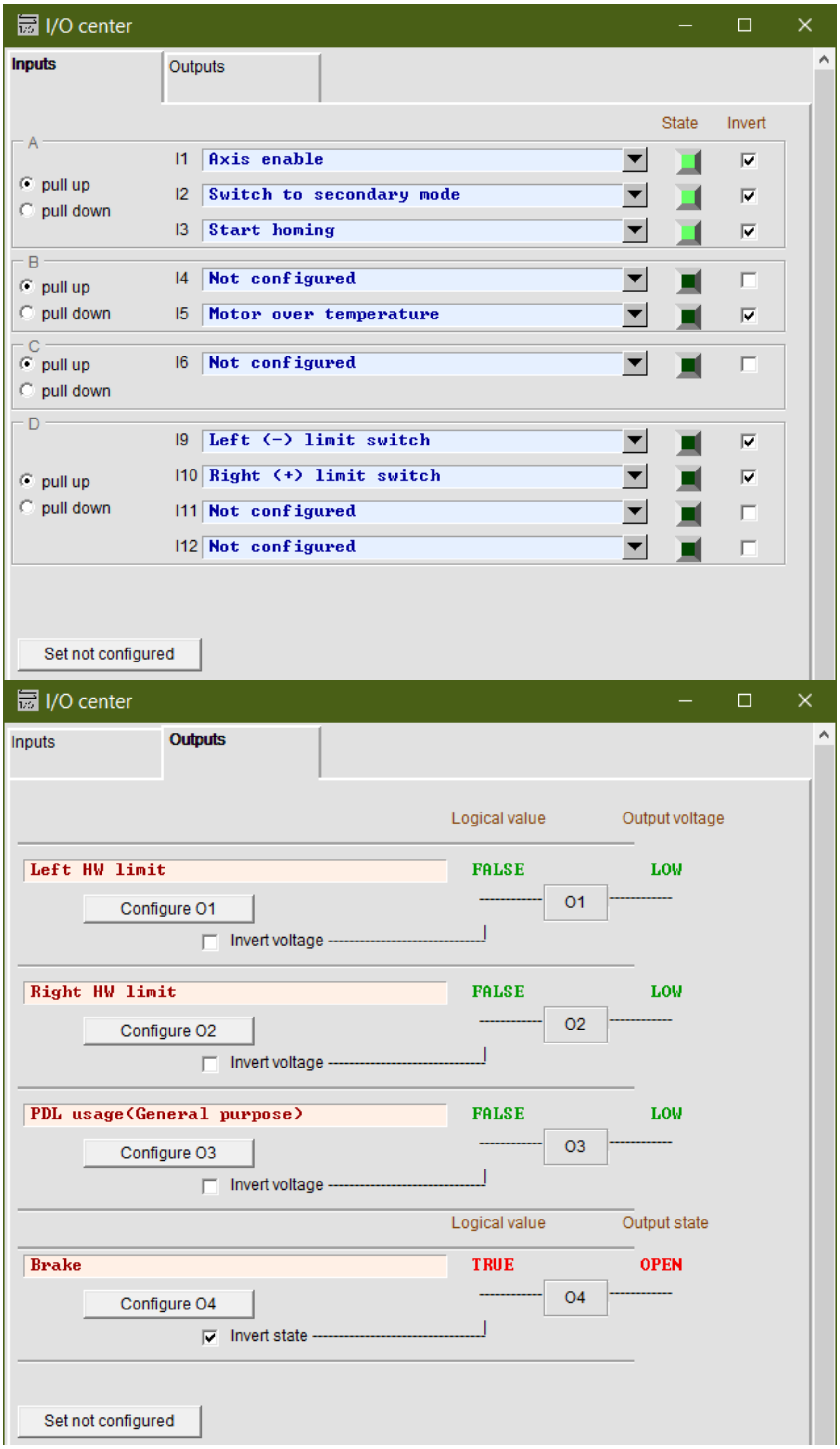

Figure 17: This figure illustrates the configuration of the HIWIN D1 drive's I/O functionalities.

open up a window with multiple tabs. Selecting the "Analog Input" tab will result in Fig. 18, where the users can press the "Set offset" button to minimize the effect of the analog signal's bias.

The safety mechanism is the final configuration that needs to be made in the Lightning software. To do so, we need to select "Conf./Tune->Protection Center" which will result in Fig. 19, where

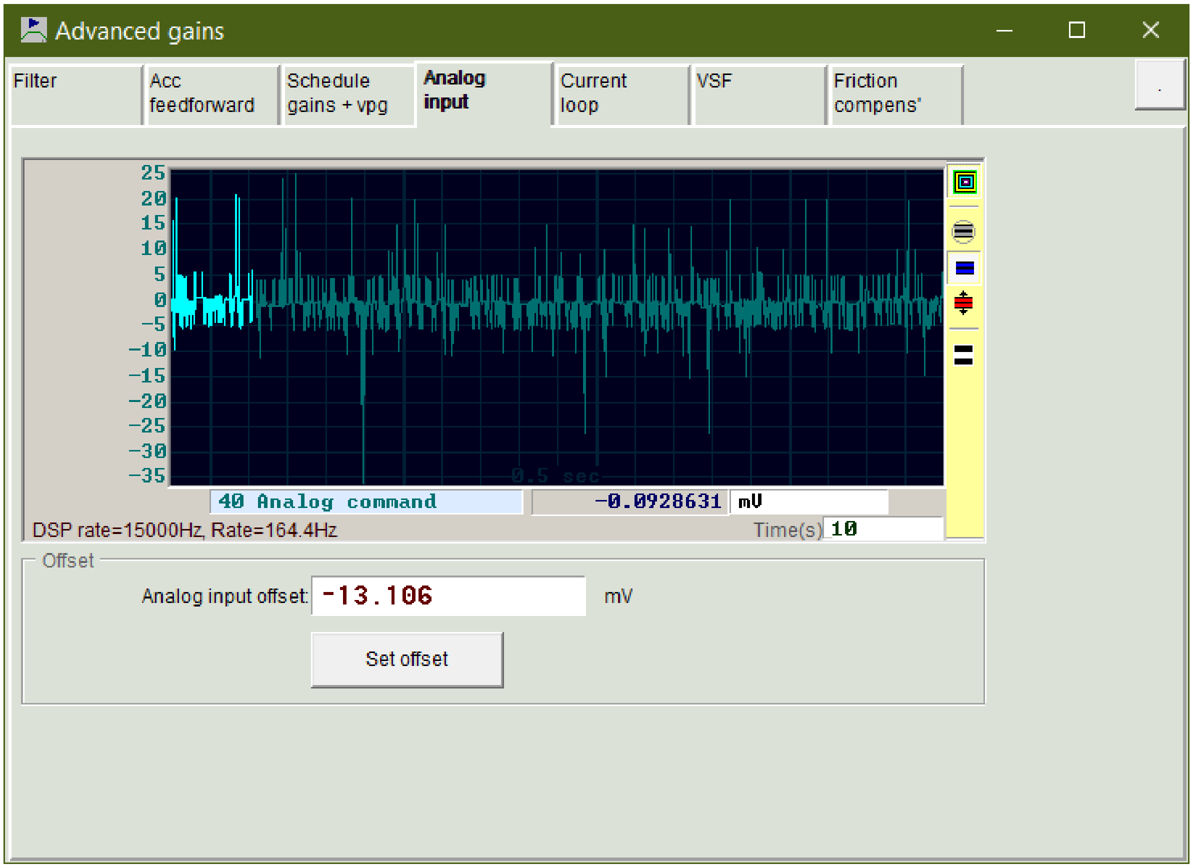

Figure 18: This figure illustrates the bias correction of the analog signal.

one can set up the software position, velocity, and acceleration limit. Set up a narrow range for the limits and gradually increase it if the desired position, speed, or acceleration is out of range. This safety measure prevents unwanted high-speed movement of the linear motor during the development phase of the controller.

The configurations of Lightning software in this section allow quick setup of the linear motor. For more details on using the Lightning software and its other functionalities, please refer to sections 4 and 5 of HIWIN D1 drive's user manual [8].

F..2 Software Set Up of the Simulink Model Details

The Simulink Real-Time is used to develop the controller used in the real-time pendulum experiments. The Simulink Real-Time and Speedgoat must be appropriately configured to read the sensors and output control signals. The first step of the configuration physically connects the Speeadgoat machine with the host computer using an Ethernet cable. This connection allows the user-programmed Simulink controller to be downloaded to the Speedgoat device and run in real-time. Moreover, this connection also enables the Speedgoat to send back the sensor signal at the run time and display it on the host computer using the "Data Inspector" function of the Simulink. Matlab and Speedgoat already have numerous tutorials on setting up the connection between the Speedgoat and the host computer. Thus we will skip them in this paper. The interested user should reference the documentation of the Speedgoat for configuration details.

Two block sets must be set up and used in the Simulink file to configure the I/O module IO-191

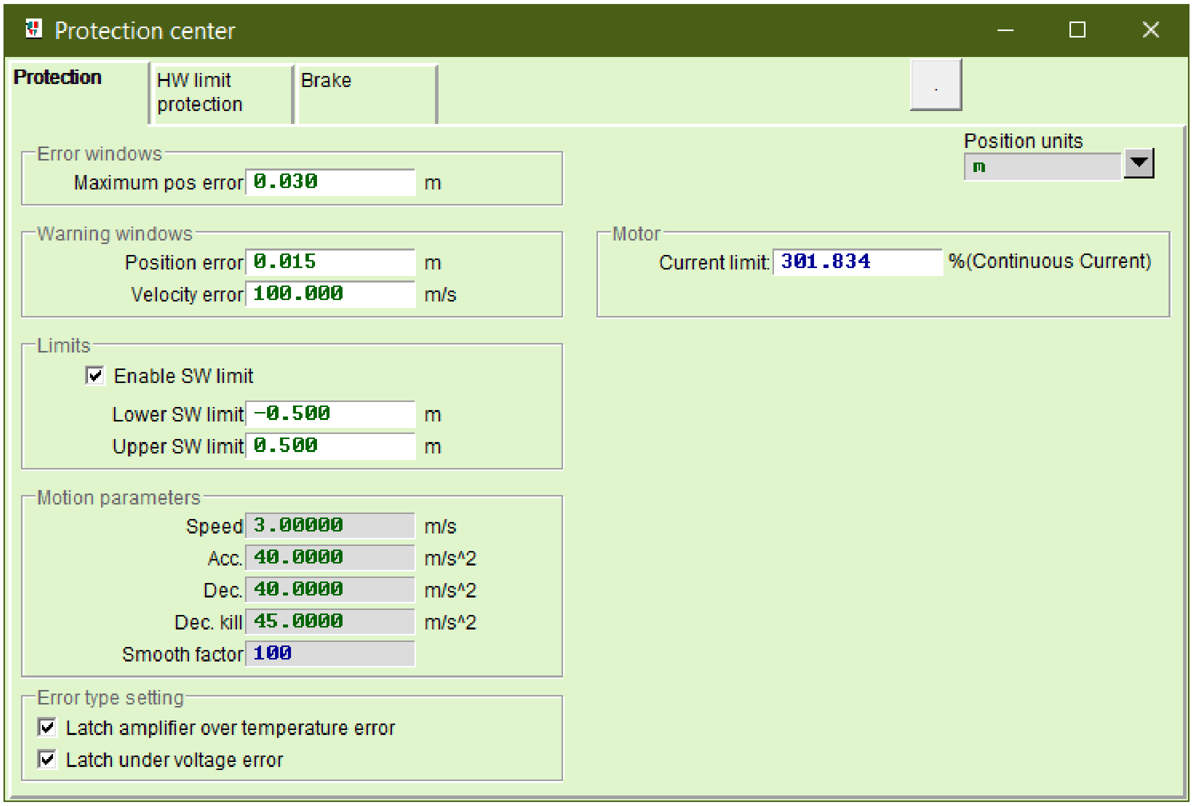

Figure 19: This figure illustrates the software safety configuration by using Lightning software.

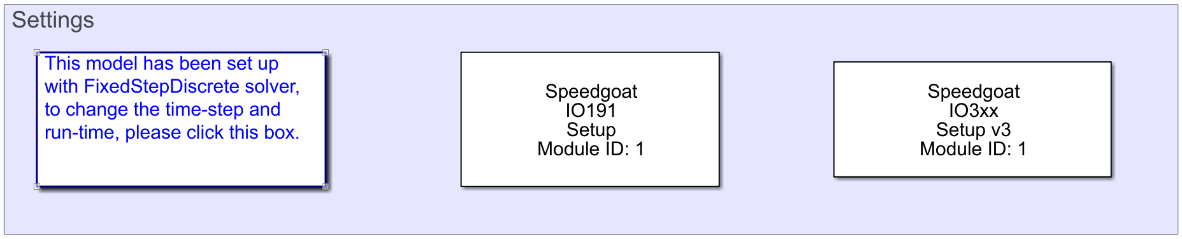

Figure 20: This figure illustrates the two block sets used in the Simulink model that configures the Speedgoat IO-191 and Speedgoat IO-392 modules.

and FPGA module IO-392 for I/O functionalities and encoder reading. They are the "Speedgoat IO-191 Setup" block and "Speedgoat IO-392 Setup" block shown in Fig. 20. Speedgoat provides those block sets when purchasing the Speedgoat machine through an active maintenance agreement. Due to the user agreement, the authors can not share those two block set installers. The interested reader should contact Speedgoat for purchase and installation guidance. To set up the I/O module, double click on the "Speedgoat IO-191 Setup" block and configure the "Analog Outputs" and "Digital I/Os" as Fig. 21 shows. In the "Analog Outputs" tab, one of the channels is used as analog output with a range of $\pm 10V$. This analog output connects to the motor drive's analog input for the linear motor's speed control in velocity mode. In the digital I/O tab, the first eight channels are used as the digital output ports, while the rest are configured as digital input ports. Three of the digital outputs are used to control the "Hardware Enable," "Switch to Secondary Mode," and "Start Homing" functions of the motor drive, as Fig. 17 shows. Two of the digital inputs are used to read the signals of limit

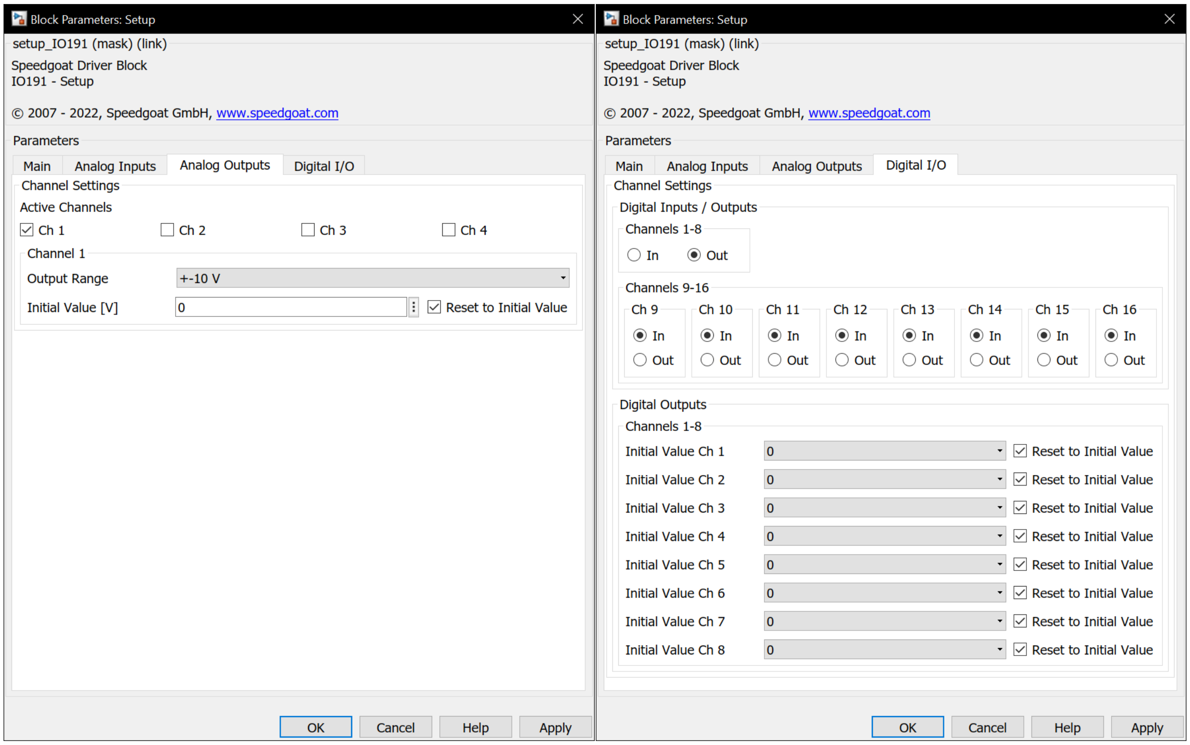

Figure 21: This figure illustrates the configuration of the IO-191 module's Simulink block. One of the analog signal outputs is selected to control the velocity of the pendulum cart in velocity mode. Meanwhile, eight ports of the digital I/O block are chosen as input ports, and the rest are set to output ports.

switches. Table. 2 shows the detailed connection. The above process completes the IO-191 module configuration for real-time digital I/O functionalities.

To set up the IO-392 block set, double click on the "Speedgoat IO3XX Setup v3" block and select the "IO-392-50k" for "IO3xx I/O Module" drop-down list. Moreover, the configuration file (Bitstream) "speedgoat_IO392_50k_CI_02385" is used as Fig. 22 shows. This bitstream file needs to be in the same folder as the Simulink file. Moreover, this file can be thought of as the driver of the FPGA module. It is purchased separately and is provided by the Speedgoat according to the user's need. The interested reader should contact Speedgoat for a quote.

Fig. 23 shows the start and homing functionalities of the linear motor. A toggle switch is used in the simulation file to hardware start the linear motor. This toggle switch controls the "Digital I/O 01" output of the IO-191 module. Thus, when the toggle is switched on, meaning the production of pin 1b is high, the D1 drive will receive an "activate" signal on the third pin of the CN2, and thus hardware activates the linear motor as configured in Fig. 17. When hardware-enabled, the default operating mode the linear motor is in is the stand-alone mode. Users can switch on the "Velocity Mode" toggle to enter the velocity mode. This action will send out a high signal on the 2b pin of the Speedgoat machine, which is connected to the fourth pin of the D1 drive's CN2 channel, which will then set the motor drive to the velocity mode as Fig. 17 configures. When turned on the first toggle and turned off the second toggle, the D1 drive will be in stand-alone mode. This action sets the "Digital I/O 03" to high so that the motor drive receives the "Start Homing" command and positions the linear motor to the middle point of the left and right limit switches. In Fig. 23, the "IO-191 Digital Input" block receives the signals of limit switches. In real-time execution, those

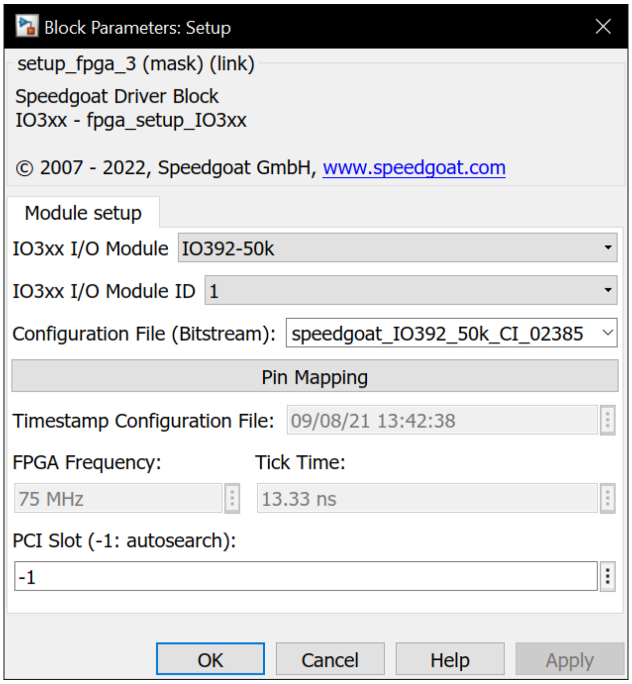

Figure 22: This figure illustrates the configuration of the IO-392 module's Simulink block. The bitstream file provided by Speedgoat is needed to set up the IO-392 block to read encoder data properly.

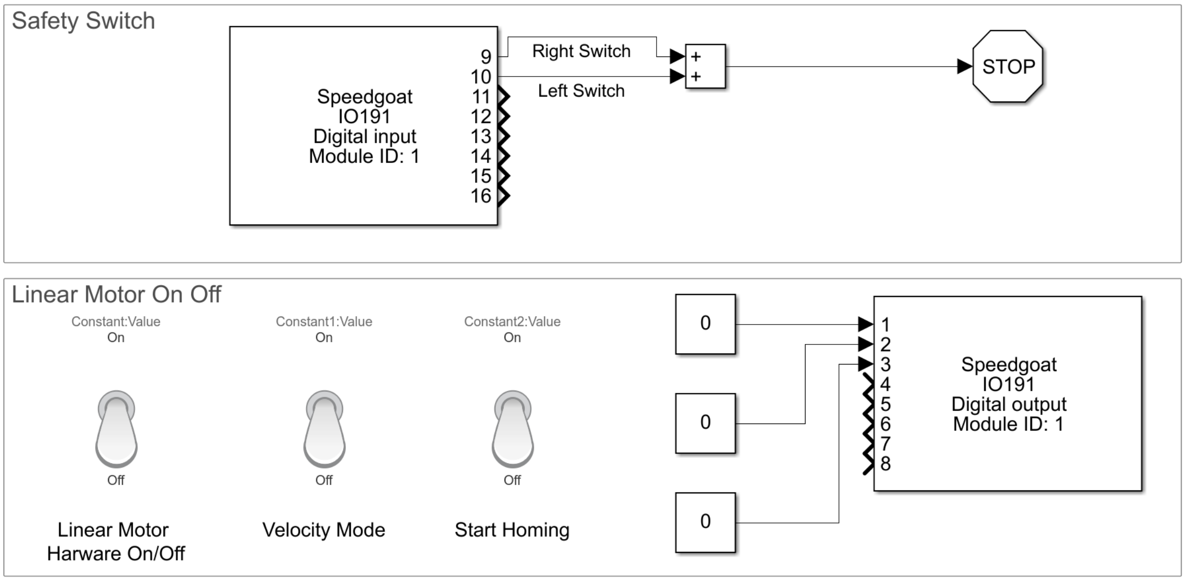

Figure 23: This figure shows enabling and disabling of the linear motor in Simulink. It also indicates how to switch the operating mode of the linear motor. By turning on the "Start Homing" toggle, the linear motor can start homing functionality in the stand-alone mode. The left and right limit switch signal is read and added up to stop the experiments if the limit switch is triggered. If one of the signals is turned to high, it will activate the "Stop Simulation" button of the Simulink, and the entire experiment is terminated.

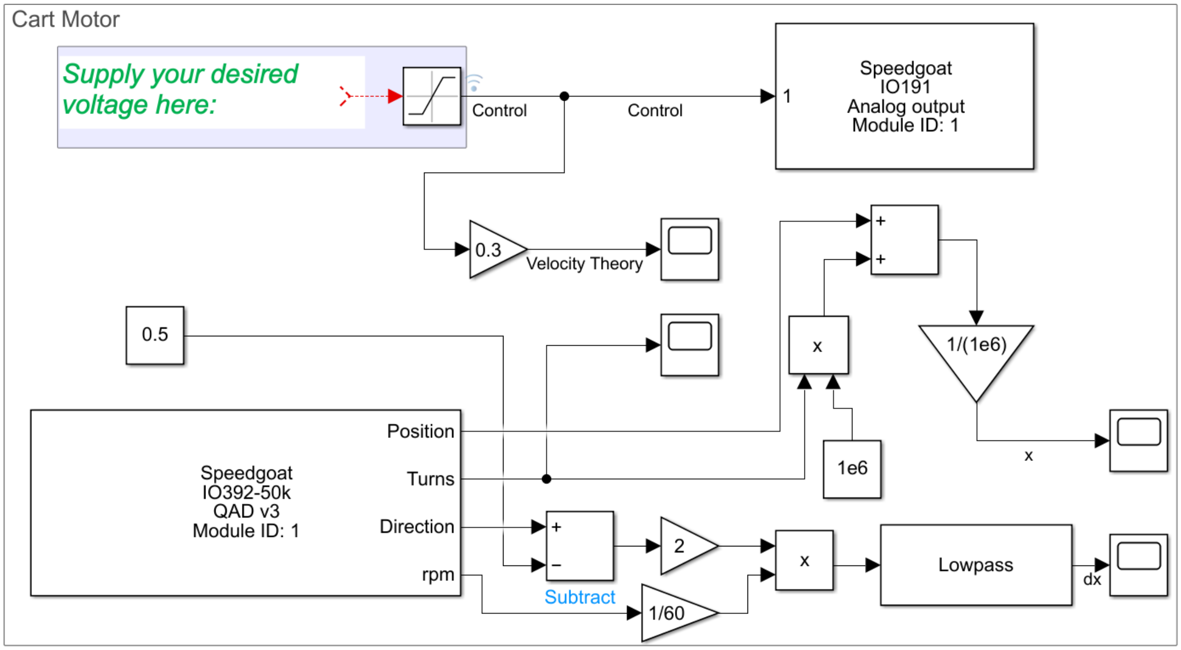

Figure 24: This figure illustrates the configuration of the pendulum cart in the Simulink model. To control the linear motor's velocity, the user should input desired analog voltage to the "Analog output" block. The output of the analog signal will be received by the D1 drive to control the linear motor to the user-defined speed. The "QAD V3" block is used to read the position and velocity of the linear motor. Moreover, some post-processing is needed to read the correct value of the linear motor's speed and velocity.

signals are constantly checked. When the limit switch is not triggered, the reading is low and vice versa. Thus, by summing them up together and connecting the summation to the "Stop Simulation" button, the simulation can be terminated when any limit switches get activated. The termination of the run file will set the output of all the ports to the low state (default), setting the linear motor to hardware disabled mode. This completes the setting up of the digital I/O functions in the Simulink file.

The desired analog voltage output of the IO-191 module needs to be supplied as Fig. 24 shows to control the linear motor's motion. A saturation block limits the voltage input range in $\pm 10V$ to avoid overflow issues. The desired voltage output is calculated using

$$Volts = \frac{v}{K_a}, \tag{1}$$

where $Volts$ is the desired analog voltage output, v is the desired velocity of the linear motor, and K_a is the scaling factor between the two. In our set up, we used $K_a = 0.3$ as Fig. 16 shows. The analog output of the IO-191 module connects the analog input of the D1 drive so that the motor drive can receive the velocity command. The analog ground of the IO-191 and motor drive is connected as well. Table. 2 shows the detailed wiring, thus completing the controlling of the linear motor velocity.

Fig. 24 also shows the reading of the encoder signal. The "IO-392-50K QAD V3" block is used to read the encoder signal. This block is purchased along with the IO-392 module. To set up this block, one needs to double click on it and then set the correct channel vector number of the encoders one wants to read in the "Module setup" tab. As Table. 1 shows, the linear motor's encoder is wired

to the fourth encoder channel. Thus, "[4]" should be entered in the "Channel vector" blank of the encoder block. Next, the reading mode of the encoder is configured in the "Quadrature decoder" tab by setting the "Operating mode" as "Quadrature decoder" and "Sampling" as "$4\times$". The "Latch mode" is set to "Disabled" while "Interpolate inner position" is turned on. The correct "Steps per revolution vector" needs to be configured, which defines the counting range of the encoder counter. In the case of the linear motor's encoder, its resolution is $1\mu m$, which means there are 10^6 counts per meter. Thus, this value is entered as 10^6 (or 4×10^6). The default reading behavior of the encoder block wraps around to zero if the encoder reading value is larger than the value entered in the "Steps per revolution vector" blank. Some post-processing needs to be done to avoid this and achieve continuous reading that does not warp around. This is achieved by first selecting the "Show position," "Show turns output," "Show speed output," and "Show direction flag output" in the "Input and output port configuration" tab of the block. Fig. 24 shows the resulted output ports of the encoder reader block. The "Turns" output will change accordingly whenever the encoder disk or linear motor performs a complete revolution. If the steps increase and a full revolution is made, then the "Turns" increase by one and vice versa. Thus, the total number of steps reflecting the absolute position of the linear motor or encoder disk can be calculate using

$$Absolute\ Steps = Steps + Turns \times (Steps\ per\ Revolution), \tag{2}$$

where the "Steps" variable is the output of the "Position" port of the encoder reader block, "Turns" variable is the output of the "Turns" port. To transfer the $Absolute\ Steps$ into the "$AbsolutePosition$" of the linear motor, the "$Steps\ per\ Revolution$" is used to divide the $Absolute\ Steps$, which will result the absolute position value of the linear motor (in m). This can be described as

$$Absolute\ Position = \frac{Absolute\ Steps}{Steps\ per\ Revolution}, \tag{3}$$

which completes the task of position reading of the linear motor. Sometimes, the velocity of the linear motor is also wanted, and it can be calculated using the "rpm" and "Direction" output port. The first thing to do is transfer the "rpm" into rounds per second. Dividing the output of the "rpm" port by 60 achieves this task. However, the output of the "rpm" port is the absolute value of the velocity. Thus, to include directional information, the "Direction" port's output is transformed into 1 or -1, where 1 represents positive velocity while -1 represents the negative velocity. Then, it is multiplied with rounds per second to get the directional speed of the linear motor. The user can add a "Lowpass" filter block to denoise the encoder's velocity measurement, which completes the reading of the linear motor encoder.

Fig. 25 shows the user can configure a similar setup to read the angular position of the pendulum arm, and we use double pendulum encoder reading as an example to illustrate this. First, as we did in the linear motor encoder reading configuration, the "Channel vector" value of two blocks is defined. As Table. 1 indicates, the first arm's encoder channel vector should be "[1]," the second arm's channel vector should be "[2]", and when using the triple pendulum arms, the third encoder's channel vector should be "[3]". Next, the "Operating mode" is selected as "Quadrature decoder," and "Sampling" is selected as "$4\times$". Similarly, the "Latch mode" is set to "Disabled" while "Interpolate inner position" is turned on. The "Steps per revolution vector" is set to 4×10^4. To read the absolute rotational angle of the pendulum arm in radius, the following equation is used

$$Absolute\ Rotational\ Angle = \frac{Absolute\ Steps}{Steps\ per\ Revolution} \times 2\pi. \tag{4}$$

Moreover, the user can calculate the angular speed of the pendulum arm in radius by multiplying the output of the "rpm" port with $1/60$ to transfer it to the rounds per second. Next, it can be

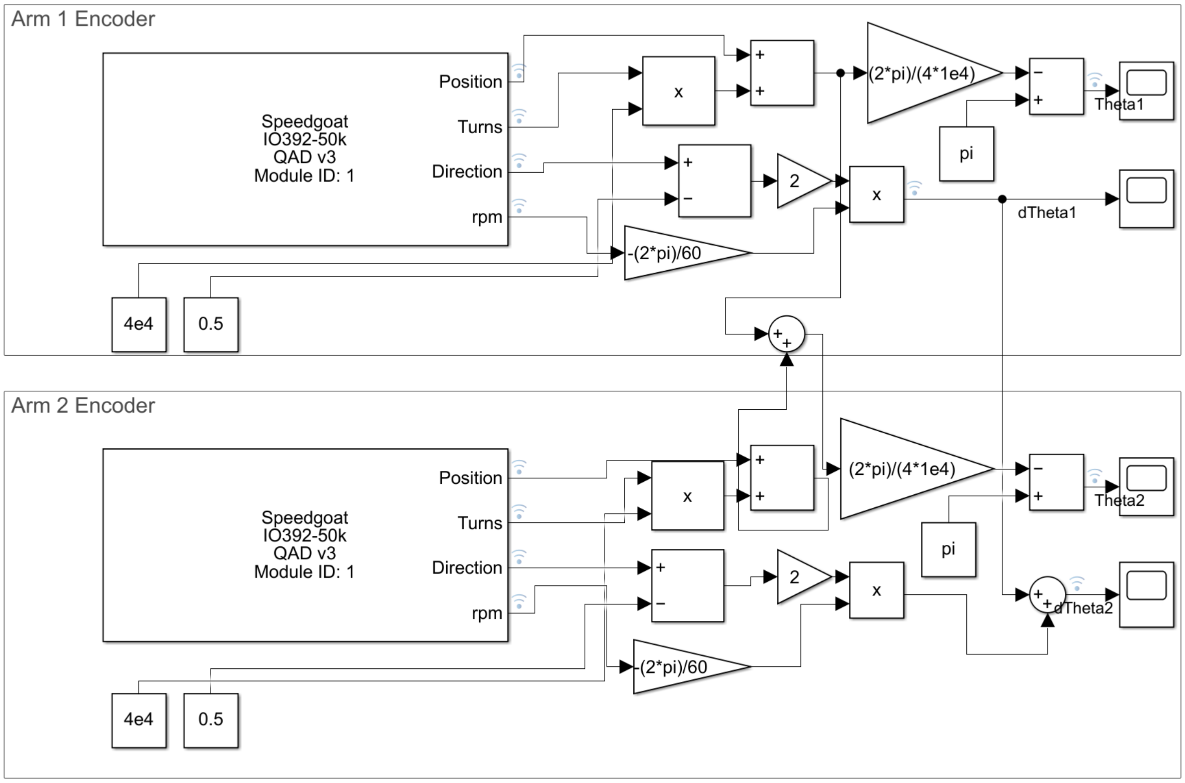

Figure 25: This figure illustrates how to read the angular position of the double pendulum shown in Fig. 1 of the paper. Conversion in Eq. (5) and Eq. (6) is needed to read the angular position of the second pendulum arm defined in Fig. 1 of the paper. A similar configuration of the encoder block is performed as we did in Fig. 24 with a different value for the "Steps per revolution vector" blank.

multiplied by $\pm 2\pi$ to transfer the velocity into rad/s. The choice of plus or minus sign determines whether the pendulum arm's clock-wise rotation increases or decreases the encoder reading. In our case, we define the clock-wise rotation of the pendulum arm increases the value of the encoder reading. Finally, it is vital to note that the reading of the second pendulum arm does not correspond to the θ_2 value shown in Fig. 1 of the paper. Define the encoder reading of the first, second, and third arm as α, β and γ. Then the relationship between the encoder reading and pendulum arm's angle θ_1, θ_2, and θ_3 can be written as

$$\theta_1 = \alpha, \tag{5a}$$

$$\theta_2 = \alpha + \beta, \tag{5b}$$

$$\theta_3 = \alpha + \beta + \gamma. \tag{5c}$$

Thus, the angular speed of the pendulum arm can be written as

$$\dot{\theta}_1 = \dot{\alpha}, \tag{6a}$$

$$\dot{\theta}_2 = \dot{\alpha} + \dot{\beta}, \tag{6b}$$

$$\dot{\theta}_3 = \dot{\alpha} + \dot{\beta} + \dot{\gamma}. \tag{6c}$$

We provide a template file that achieves all the encoder reading and linear motor control tasks[3]. The reader can directly modify this file to include their controller without performing the encoder

[3]Code availalbe at https://github.com/dynamicslab/MultiArm-Pendulum

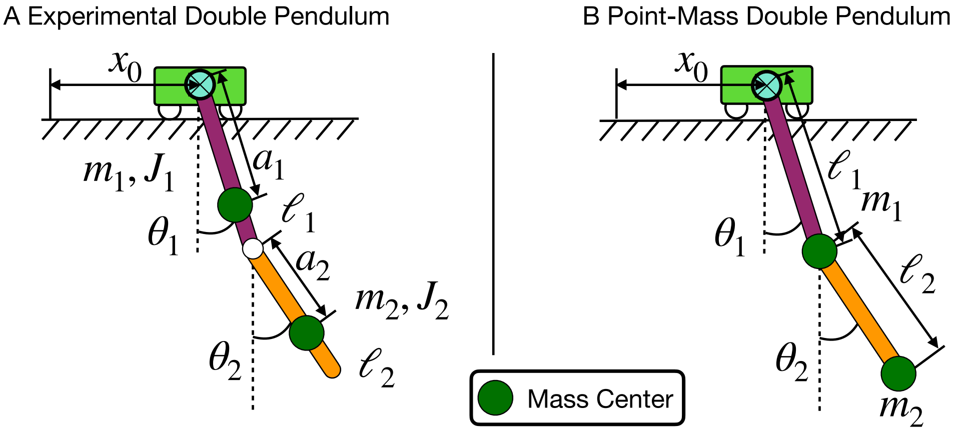

Figure 26: This figure illustrates the difference between the point-mass model of the double pendulum (left) and the more realistic experimental double pendulum (right) studied herein. The point-mass model ignores the mass of each arm of the pendulum, while the experimental model considers both the inertial (J_i) and mass (m_i) of the pendulum arms.

reading configuration. This conversion is also shown in Fig. 25, which completes the setup of the Simulink configuration.

G. Step by Step Operation Details

G..1 Pre-Experiment Preparations

Before starting the experiments, the following operations are needed:

1. Put on safety glasses.
2. Determine the overall sampling frequency of the setup, control input range, cart velocity range, and cart position range. These parameters can help determine whether the purposed experiments is physically realizable. If the desired cart velocity and acceleration range is higher than the limit introduced in Fig. 19, change the limits in Fig. 19 accordingly.
3. Develop the controller Simulink file, and make sure it compiles correctly. Rewire the connection of the IO-191 module if the custom I/O functionalities are needed.
4. Double check the wiring between the Speedgoat terminal block and pendulum arm encoder and linear motor drive.
5. Check the connection between the slip-ring and slip-ring brush. Make sure the slip-ring and slip-ring brush are contacting each other.
6. Connect the host computer and Speedgoat machine using an Ethernet cable. Turn on the Speedgoat machine and setup the Simulink Real-Time to establish communication between the host computer and Speedgoat machine. This step and those prior to it complete the connection between the pendulum arm and Speedgoat machine.
7. Check all the wires that connect the linear motor's electrical parts mentioned in Fig. 10 of the paper. Make sure all cables are connected properly according to the wiring diagram shown in Fig. 11.

8. Release the emergency stop button and connect the circuit breaker.
9. Plug in the power cord of the emergency stop button module. This power cord is used to supply power to the linear motor, motor drive, and all other components in the electrical parts of the linear motor.
10. Press the green button of the emergency stop button module to supply AC single phase power to the electrical box of the system. Wait a moment to observe if any shortage has occurred. If so, press the emergency stop button to cut off the power supply. If not, proceed to the next step.
11. Press the green momentary switch to self-lock the magnetic contactor. This will provide the power supply to the line reactor and DC power supplied module. Wait a moment to observe if any shortage has occurred. If so, press the emergency stop button to cut off the power supply. If not, proceed to the next step.
12. Set the toggle switch of the electrical box to on. This provides power to the motor drive, and in turn the motor drive will provide power to the linear motor. Wait a moment to observe if any shortage has occurred. If so, press the emergency stop button to cut off the power supply. If not, proceed to the next step.
13. Remove any and all obstacles from the motor rail.
14. Execute the Lightning software and connect the D1 motor drive to the host computer. Make sure all the parameters shown in the Lightning software are correct.
15. Open the Simulink model with developed controller. Compile it and download it to the Speedgoat machine.
16. Execute the compiled controller, start the desired experiments.

G..2 Peri-Experiment Operations and Precautions

One should adhere to the following operations and precautions below when performing experiments:

1. Never get close to the moving pendulum cart just in case any mistakes in the designed controller file make the linear motor perform undesired motion and injure the operator.
2. The operator should stand on the front or back side of the experimental setup. Never stand on the side of the linear motor as one may be struck by a pendulum arm.
3. The operator should closely monitor the behavior of the experimental setup. If for any reasons there is an electical shortage, smoke, heat, or unwanted noise, immediately press the emergency stop button to cut off the power supply to the linear motor to avoid further damage.
4. The operator should closely monitor the surroundings of the experimental setup. If any person, animal, or unwanted object enters the experimental area the experiment should be stopped immediately to avoid injury and/or prevent the damage of the experimental setup.

5. The operator should continually check the data being collected from the experiment. If an unwanted measurement drift is apparent in the plotted data, immediate stop the experiment by pressing the stop simulation button of the Simulink file.

G..3 Post-Experiment Operations

After an experiment, the following operation should be performed:

1. Check all wiring related to the electrical component of the pendulum setup. Reconnect any wires that may have become disconnected during the experiment.
2. Check for damage to the system hardware.
3. Go back to the pre-experiment operations to perform another experiment or follow the proceeding steps to turn off the system.
4. Close the Lightning software to cut off the communication between the D1 drive and host computer.
5. Switch the toggle switch to off to cut off the power to the motor drive.
6. Press the red momentary switch to cut off the power to the DC power supply and line reactor.
7. Press the off button of the emergency stop button module. This will cut off all power supply to the linear motor. Then unplug the power cord.
8. Save the experimental files and data to a secured location. After saving close Matlab and Simulink.
9. Turn off the Speedgoat machine. This will cut off the power supply to the pendulum arm encoder.
10. Turn off the host computer.

With the above post-experiment operations, all of the power supplied to the experimental system is now cut off.

H. Safety Mechanism and Notes Details

The following electrical safety mechanisms should further be implemented:

1. Use the circuit breaker protect the motor from over current failure.
2. Install the limit switch on the linear motor to prevent the out of range motion of the linear motor.
3. Use the regenerative resistors to absorb extra kinetic energy during the breaking of linear motor.
4. Use the noise filter to reduce the ground current protecting the appliances that share the same ground.

5. The noise filter, shielded cable, edge filter, and EMI cores are used to reduce the side effect of EMI noise. This in turn provides a cleaner signal and prevents the possible damage caused by faulty measurements signal.
6. Obtain better control of the electrical system using the emergency stop button, momentary switches, while allowing for rapid disconnection from the power supply, thus improving safety in the case of any electrical shortage.
7. Use an electrical box to prevent potential mechanical damage to the electrical components.
8. Properly ground electrical components to avoid a shock to the operator.
9. Use the linear motor's temperature sensor to prevent overheating and over current failure.

The Lighting and Simulink software should be modified as follows to guarantee user safety:

1. Use the Lightning software to internally restrict the range of linear motor's position, velocity, and acceleration.
2. Use the Lightning software to stop the linear motor when the limit switch is triggered.
3. Program the Simulink model to limit the analog output, which restricts the velocity range of the linear motor.
4. Use the Simulink model to stop the experiment when the limit switch is triggered.

In addition to the above safety procedures, one should also use the following points to ensure the safety of the operator and surrounding personnel.

1. Always check the wire connections for damage or disconnections before starting the experimentto avoid an electrical shortage and fire.
2. Clear the linear motor rail of obstacles before supplying power to the motor.
3. Do not touch the linear motor rail by hand. This could potentially rust the motor rail.
4. Apply general purpose grease every month to protect the motor rails from rusting.
5. Never touch the linear motor while it is in operation.
6. Never perform any wiring of electrical components with the power supply on.
7. Keep a safe distance from the system while it is operating. We suggest the operator and all surrounding personnel should be at least one meter from the experimental setup while performing experiments.
8. Do not stand to the side of the pendulum arms as this increases the risk of being hit by a pendulum arm.
9. Do not expose the electrical components to moisture or liquid as they are not waterproof. Furthermore, do not operator the equipment if hands are wet.
10. Wear safety glasses for all construction and operation steps for the experiment.

Table 4: Comparison of the estimated parameters and CAD model estimated parameters of the single, double, and triple pendulum. The length l_1, l_2 and l_3 are not estimated for the single, double, and triple pendulum respectively, since they are not present in the corresponding EOM. It is interesting to note that the estimated values of the local gravity constant g are different for all three pendulums. This is caused by the fact the g is an optimization parameter during the parameter estimation and is allowed to vary to best fit the data. Units: mass kg, length m, inertia kgm^2, and local gravity constant m/s^2.

Pendulum Type	Single		Double		Triple	
Source / Vars	CAD	Estimated Parameters	CAD	Estimated Parameters	CAD	Estimated Parameters
m_1	0.1183	0.1476	0.1199	0.0938	0.11	0.2582
m_2			0.1183	0.1376	0.12	0.2794
m_3					0.1	0.1186
l_1	0.17272		0.17272	0.1727	0.1727	0.1728
l_2			0.2286		0.2286	0.2287
l_3					0.2413	
a_1	0.08638	0.1478	0.08755	0.1086	0.08755	0.16
a_2			0.1256	0.1168	0.127	0.2029
a_3					0.12	0.1837
J_1	0.001578	1.0912×10^{-4}	0.001627	10^{-4}	0.001578	1×10^{-4}
J_2			0.002908	10^{-4}	0.0029	3.1416×10^{-4}
J_3					0.002157	1.6874×10^{-4}
ε_1		2.2394×10^{-4}		10^{-5}		0.0015
ε_2				10^{-5}		2.5958×10^{-4}
ε_3						1×10^{-5}
g	9.8083	9.81001	9.8083	9.808	9.8083	9.8083

I. Parameter Estimation Details

This section gives the details of parameters estimation process shown in Table. 4. In this Appendix., we will first use the EOM of the double pendulum on the cart as an example to explain why it is desired to use the acceleration of the pendulum cart as our control input. Then, we will show more details on the parameter estimation process we used to identify the parameters in Table. 4. Since the model derivation task of single, double and triple pendulum are standard text book example, we refer the reader to check [5, 9] for more details on deriving the EOM of pendulum arm on the cart.

I..1 Equations of Motion

This section shows why it is desired to use the acceleration of the pendulum cart as control input and we illustrate this using double pendulum as an example. Let x_0 be the position of pendulum cart and v_{x_0} its velocity, and the rotational angle of pendulum arm as θ_1 and θ_2 respectively. Following the modeling process shown in [5], the second derivative of the double pendulum's

rotational angle can be written as $\ddot{\theta}_1$ and $\ddot{\theta}_2$, giving

$$\ddot{\theta}_1 = \frac{A_{10}\sin(\theta_1) + A_{11}\sin(\theta_1-\theta_2) + A_{12}\sin(\theta_1-2\theta_2) + A_{22}\sin(2\theta_1-2\theta_2) + \varepsilon_1 F_{11} + \varepsilon_2 F_{12} + C_1 \dot{v}_{x_0}}{D(\theta_1-\theta_2)}$$
$$\ddot{\theta}_2 = \frac{B_{01}\sin(\theta_2) + B_{11}\sin(\theta_1-\theta_2) + B_{21}\sin(2\theta_1-\theta_2) + B_{22}\sin(2\theta_1-2\theta_2) + \varepsilon_1 F_{21} + \varepsilon_2 F_{22} + C_2 \dot{v}_{x_0}}{D(\theta_1-\theta_2)} \tag{7}$$

where

$$\begin{aligned}
A_{10} &= -l_1 g a_2^2 m_2^2 - 2a_1 g m_1 a_2^2 m_2 - 2J_2 l_1 g m_2 - 2J_2 a_1 g m_1\\
A_{11} &= -2l_1 a_2^3 \dot{\theta}_2^2 m_2^2 - 2J_2 l_1 a_2 \dot{\theta}_2^2 m_2\\
A_{12} &= -l_1 a_2^2 g m_2^2\\
A_{22} &= -l_1^2 a_2^2 \dot{\theta}_1^2 m_2^2\\
B_{01} &= -a_2 m_2 (g m_2 l_1^2 - g m_1 l_1 a_1 + 2 g m_1 a_1^2 + 2J_1 g)\\
B_{11} &= a_2 m_2 (2m_2 l_1^3 \dot{\theta}_1^2 + 2m_1 l_1 a_1^2 \dot{\theta}_1^2 + 2J_1 l_1 \dot{\theta}_1^2)\\
B_{21} &= a_2 m_2 (g m_2 l_1^2 + a_1 g m_1 l_1)\\
B_{22} &= 2m_2 (g m_2 l_1^2 + a_1 g m_1 l_1)\\
F_{11} &= -2\dot{\theta}_1 m_2 a_2^2 - 2J_2 \dot{\theta}_1\\
F_{12} &= 2J_2\dot{\theta}_2 - 2J_2\dot{\theta}_1 - 2a_2^2\dot{\theta}_1 m_2 + 2a_2^2\dot{\theta}_2 m_2\\
&\quad - 2l_1 a_2 \dot{\theta}_1 m_2 \cos(\theta_1-\theta_2) + 2l_1 a_2 \dot{\theta}_2 m_2 \cos(\theta_1-\theta_2)\\
F_{21} &= 2l_1 a_2 \dot{\theta}_1 m_2 \cos(\theta_1-\theta_2)\\
F_{22} &= 2J_1\dot{\theta}_1 - 2J_1\dot{\theta}_2 + 2l_1^2\dot{\theta}_1 m_2 - 2l_1^2\dot{\theta}_2 m_2 + 2a_1^2\dot{\theta}_1 m_1 - 2a_1^2\dot{\theta}_2 m_1\\
&\quad + 2l_1 a_2 \dot{\theta}_1 m_2 \cos(\theta_1-\theta_2) - 2l_1 a_2 \dot{\theta}_2 m_2 \cos(\theta_1-\theta_2)\\
C_1 &= l_1 a_2^2 m_2^2 \cos(\theta_1-2\theta_2) - 2J_2 l_1 m_2 \cos(\theta_1) - l_1 a_2^2 m_2^2 \cos(\theta_1)\\
&\quad - 2J_2 a_1 m_1 \cos(\theta_1) - 2a_1 a_2^2 m_1 m_2 \cos(\theta_1)\\
C_2 &= 2l_1^2 a_2 m_2^2 \cos(\theta_1-\theta_2)\cos(\theta_1) - 2J_1 a_2 m_2 \cos(\theta_2) - 2l_1^2 a_2 m_2^2 \cos(\theta_2)\\
&\quad - 2a_1^2 a_2 m_1 m_2 \cos(\theta_2) + 2l_1 a_1 a_2 m_1 m_2 \cos(\theta_1-\theta_2)\cos(\theta_1)\\
D(\theta_1-\theta_2) &= -l_1^2 a_2^2 m_2^2 cos(\theta_1-\theta_2)^2 + l_1^2 a_2^2 m_2^2 + J_2 l_1^2 m_2 + m_1 a_1^2 a_2^2 m_2\\
&\quad + J_2 m_1 a_1^2 + J_1 a_2^2 m_2 + J_1 J_2
\end{aligned} \quad . \tag{8}$$

Here we use the convention that A_{ij}, B_{ij} are the coefficients of the energy preserved Hamiltonian system for the $\ddot{\theta}_1$ and $\ddot{\theta}_2$ equations, respectively, while the subscripts denote the coefficients on the θ_1 and θ_2 terms inside the sine function they are multiplied against. The F_{ij} are the $(\theta_1, \theta_2, \dot{\theta}_1, \dot{\theta}_2)$-dependent friction terms in $\ddot{\theta}_i$ equation with friction coefficient ε_j. Finally, the C_i terms correspond to the influence of the cart velocity, $\dot{v}_{x_0}$, for the $\ddot{\theta}_i$ equation and $D(\theta_1-\theta_2)$ represents the denominator function. By writing the equation of the of the experimental double pendulum in the form of Eq. (7), one can see the acceleration of the pendulum cart $\dot{v}_{x_0}$ can be nicely used as control input of the system. By **assuming** perfect velocity control can be provided by the motor drive, the mass of the pendulum cart M can be avoided in the equation of motion of the pendulum cart.

In experiments with the physical double pendulum on a cart the velocity of the pendulum cart is controlled by the servo drive by applying an analog voltage input as we mentioned in Sec. 5.1. This voltage input is calculated by first taking the integral of the acceleration of the pendulum cart

$\dot{v}_{x_0}$. The integration will result the desired velocity of the pendulum cart v_{x_0}. Then the desired velocity is scaled to the corresponding voltage using the scaling factor shown in Fig. 16. Thus, in the experiments, the acceleration of the double pendulum is chosen as the control input of the system. To account for this control input, we write out the complete first order ODE of the pendulum on the cart system using acceleration of the cart as control input, denoted u. The final result is the ODE

$$
\begin{aligned}
\dot{\theta}_1 &= \omega_1, \\
\dot{\theta}_2 &= \omega_2, \\
\dot{\omega}_1 &= \frac{A_{10}\sin(\theta_1) + A_{11}\sin(\theta_1-\theta_2) + A_{12}\sin(\theta_1-2\theta_2) + A_{22}\sin(2\theta_1-2\theta_2) + \varepsilon_1 F_{11} + \varepsilon_2 F_{12} + C_1 u}{D(\theta_1-\theta_2)}, \\
\dot{\omega}_2 &= \frac{B_{01}\sin(\theta_2) + B_{11}\sin(\theta_1-\theta_2) + B_{21}\sin(2\theta_1-\theta_2) + B_{22}\sin(2\theta_1-2\theta_2) + \varepsilon_1 F_{21} + \varepsilon_2 F_{22} + C_2 u}{D(\theta_1-\theta_2)}, \\
\dot{x}_0 &= v_{x_0}, \\
\dot{v}_{x_0} &= u.
\end{aligned}
\tag{9}
$$

System 9 therefore represents the full equations of motion for the double pendulum on a cart that we investigate for the parameter estimation task. The equations of motions shown in Eq. (9) made various assumptions. For example, it did not consider the the air drag of the pendulum arm, it did not consider the effect of rolling bearing balls during the rotational movement as shown in [10], it did not consider the time-lag between the control signal and movement of the linear motor, it did not consider the oscillation of the pendulum, it did not consider the fact that linear motor might not be moving in a horizontal line, etc. However, it does a pretty good job on capturing most of the system dynamics and is the most widely used equation of motion for the experimental double pendulum on the cart system. If the user consider more factors, a more accurate and complicated model of the double pendulum on the cart can be derived. The same procedures shown above can also be used to derive the equation of motion of the single and triple pendulum on the cart. A good reference on deriving the equation of motion of the triple pendulum can be seen in [9].

I..2 Parameter Estimation

Having now derived the equations of motion for the experimental double pendulum on the cart, we now seek to identify the values of the numerous parameters to arise in it. Our goal is to align the parameters of the model (9) with those of our constructed double pendulum on a cart. Of course, the parameters of the physical model are uncertain and we expect a difference between the actual and theoretical parameter values due to imperfect manufacturing, installation tolerance, etc. In this subsection, we will present the method we have used for parameter estimation of the double pendulum. The same approach is also used to estimate the parameters of the double and triple pendulum.

To estimate the parameters of the double pendulum we fix the position of the cart. The result will be that $u = 0$ in (9), thus reducing the ODE to one in the four variables $(\theta_1, \theta_2, \dot{\theta}_1, \dot{\theta}_2)$, given by

$$
\begin{aligned}
\dot{\theta}_1 &= \omega_1, \\
\dot{\theta}_2 &= \omega_2, \\
\dot{\omega}_1 &= \frac{A_{10}\sin(\theta_1) + A_{11}\sin(\theta_1-\theta_2) + A_{12}\sin(\theta_1-2\theta_2) + A_{22}\sin(2\theta_1-2\theta_2) + \varepsilon_1 F_{11} + \varepsilon_2 F_{12}}{D(\theta_1-\theta_2)}, \\
\dot{\omega}_2 &= \frac{B_{01}\sin(\theta_2) + B_{11}\sin(\theta_1-\theta_2) + B_{21}\sin(2\theta_1-\theta_2) + B_{22}\sin(2\theta_1-2\theta_2) + \varepsilon_1 F_{21} + \varepsilon_2 F_{22}}{D(\theta_1-\theta_2)}
\end{aligned}
\tag{10}
$$

Next, the experimental data of the pendulum arms is collected by swinging them from an arbitrary initial condition. The relative angles of pendulum arms with respect to its joint is measured using the encoders, with 10000 CPR accuracy in quadrature mode, which results in 40000 PPR (pulse per revolution). Suppose that we have collected $n \geq 1$ measurements of the values of $(\theta_1, \theta_2, \dot{\theta}_1, \dot{\theta}_2)$, each over $L \geq 1$ time values. We arrange each measure into the data matrices $\mathbf{Y}_i \in \mathbb{R}^{4\times L}$ for each $i = 1, \dots, n$ and denote $\hat{\mathbf{Y}}_i(p) \in \mathbf{R}^{4\times L}$ the data generated by simulating (10) with the same initial conditions for each $i = 1, \dots, n$ and a given choice of the parameter values

$$p = [m_1,\ m_2,\ a_1,\ a_2,\ \ell_1,\ J_1,\ J_2,\ \varepsilon_1,\ \varepsilon_2,\ g]. \tag{11}$$

Then, the goal of our parameter estimation is to solve the optimization problem

$$\min_p \sum_{i=1}^{n} \|\mathbf{Y}_i - \hat{\mathbf{Y}}_i(p)\|_2. \tag{12}$$

Of course, a (global) minimum of the above error identifies a choice of parameters for which the trajectories of the simulated double pendulum using (10) most closely aligns with that of the physical model.

To perform our parameter estimation we have used $n = 26$ different measurements, all sampled with sampling rate of $1kHz$. Each $\mathbf{Y}_i$ consists of $L = 2667$ consecutive data points. We solve the optimization problem (12) using MATLAB's optimization toolbox. Specifically, we employ the *particleswarm* algorithm to achieve the global minimum. To better assist the convergence to the global optimum, we initialize with upper and lower bound constraints on the parameter values. These bounding values and their units are given component-wise by

$$\begin{aligned} p_l &= [0.09\text{kg},\ 0.09\text{kg},\ 0.09\text{m},\ 0.10\text{m},\ 0.172719\text{m},\ 10^{-4}\text{kgm}^2,\ 10^{-4}\text{kgm}^2,\ 10^{-5},\ 10^{-5},\ 9.808\text{m/s}^2] \\ p_u &= [0.14\text{kg},\ 0.14\text{kg},\ 0.16\text{m},\ 0.22\text{m},\ 0.172721\text{m},\ 0.002\text{kgm}^2,\ 0.002\text{kgm}^2,\ 10^{-3},\ 10^{-3},\ 9.809\text{m/s}^2]. \end{aligned} \tag{13}$$

As is apparent from (13), we begin the optimization process by being more certain of some parameter values than the other from the aid of simple measurement devices such as electric scales. In contrast, there is not a good way to directly measure the the inertial and friction coefficients of the pendulum arms, and so we have provided a larger variance in our upper and lower bounds. However, the CAD models of the pendulum arms can be used to have a rough guess of the inertial parameters. The result of the parameter estimation process using the minimization scheme (12) is

$$\begin{aligned} p_* = [&0.0938\text{kg},\ 0.1376\text{kg},\ 0.1086\text{m},\ 0.1168\text{m},\ 0.1727\text{m}, \\ &10^{-4}\text{kgm}^2,\ 10^{-4}\text{kgm}^2,\ 10^{-5},\ 10^{-5},\ 9.808\text{m/s}^2]. \end{aligned} \tag{14}$$

It should be noted that the parameters we identified in Eq. (14) is **NOT** the real physical parameters of the experimental double pendulum. Rather, it should be think of as a set of optimization variables that optimize the minimization scheme (12) while at the same time is numerically **close to** the actual experimental parameters. This is due to the fact that model we used does not capture all the dynamics of the pendulum arm and the data we collected is not perfectly clean[4]. Thus, there's rooms for the optimization algorithms to fine tune the optimization variables to compensate for unconsidered factors during the modeling. This is why the global optimization algorithm

[4]The estimated angular velocity of the pendulum arm deviates from the true velocity. This makes the forward simulation of Eq. 10 accumulates error due to the chaotic behavior of the double pendulum. This deviation of estimated velocity of the pendulum arm makes the estimated parameters deviates from the true physical value as well.

is used to find out a set of parameters that best compensate those unmodeled factors. Another important thing to note is, the identified model parameters provides a good simulated forward prediction performance if the dynamics of the double pendulum under prediction is similar to those used in parameters estimation. In other words, using data set that covers different parts of the double pendulum's phase space will result various optimal solution for Eq. (14). This happens quite frequently when the control input is applied to the pendulum on the cart system, where often times an extra step of parameter tuning is needed to compensate the unmodeled behavior of the pendulum as shown in [5, 9, 11]. Another interesting thing we noticed is that the parameters estimated using a higher energy level of double pendulum's motion requires a longer time to converge to the optimal solution. Moreover, it might not provide a good forward prediction performance for the low energy motions. We suspect the increase of the training time is due to the fact that the higher energy level motion is more sensitive to the change of parameters. As for the worse prediction performance on the low energy data, we believe it is caused by too much tuning been made in the high energy level data set to compensate for the unmodeled factors, since the unmodeled factors plays more role in the high energy data set. This is why we included $n = 26$ different measurements, including low, medium, and high energy level motion of the pendulum to get a balance performance on prediction performance. The parameter estimation task of the single and triple pendulum is similar to the one for double pendulum and is omitted here. Instead, the estimated parameter is directly given in Table. 4.

J. Bill of Materials

J..1 Bill of Materials for Building Pendulum Arm

All the materials needed to build the pendulum arm shown in Fig. 4 of the paper can be seen in Table. 5. The price of some of the parts are not shown and the readers need to request a quote from the company to get its price. Other parts such as screws are sold in pack, for those parts the price for one pack is listed and is indicated by "*" symbol. Besides the materials mentioned in Table. 5, we also recommend ordering 1008-1010 carbon steel ring shim set (1/4" ID, McMaster 3088A928) to accommodate for the manufacturing error if needed.

Table 5: Materials needed to build the pendulum arm shown in Fig. 4 of the paper.

Designator	Component	Number	Total cost-currency	Source of materials	Material type
Arm1Shaft (1)	McMaster 1346K17	1	9.61	McMaster	1566 Carbon Steel
SlipRing 8 Channel (2)	Moog AC2690-8	1	NA	Moog	Gold and Others
Arm1 (3)	McMaster 8975K224	1	27.4	McMaster	Aluminum
91251A077 (4)	McMaster 91251A077	16	8.74*	McMaster	Black-Oxide Alloy Steel
WireClip (5)	3D Printed	4	NA	NA	PLA
EncoderShim (6)	3D Printed	2	NA	NA	PLA
Encoder (7)	US Digital EM2-2-10000-I	2	87.02	US Digital	Others
91251A081 (8)	McMaster 91251A081	4	9.01*	McMaster	Black-Oxide Alloy Steel

Table 5 continued from previous page

Designator	Component	Number	Total cost-currency	Source of materials	Material type
BrushShim (9)	3D Printed	1	NA	NA	PLA
BrushBlock (10)	Moog AC259-5	1	NA	Moog	Gold and Others
3088A449 (11)	McMaster 3088A449	2	7.13*	McMaster	Steel
R188 [5] (12)	R188 Hybrid Ceramic Bearing	4	105.60	Ortech	Ceramic, Steel, and Others
Arm2Shaft (13)	McMaster 1346K11	1	8.62	McMaster	1566 Carbon Steel
SlipRing 5 Channel (14)	Moog AC2690-5	1	NA	Moog	Gold and Others
Arm2 (15)	McMaster 8975K224	1	27.4	McMaster	Aluminum
97022A213 (16)	McMaster 97022A213	2	5.34*	McMaster	316 Stainless Steel
EncoderDisk (17)	US Digital HUBDISK-2 -10000-250-IE	2	74.16	US Digital	Others
98126A447 (18)	McMaster 98126A447	2	6.82*	McMaster	18-8 Stainless Steel
97633A130 (19)	McMaster 97633A130	2	9.77*	McMaster	1060-1090 Spring Steel
BearingPlate (20)	McMaster 89015K255	2	31.7	McMaster	Aluminum
Arm1Cover (21)	3D Printed	1	NA	NA	PLA
Arm3Shaft (23)	McMaster 1346K11	1	8.62	McMaster	1566 Carbon Steel
Arm3 (23)	McMaster 8975K224	1	27.4	McMaster	Aluminum
Arm2Cover (24)	3D Printed	1	NA	NA	PLA

J..2 Bill of Materials for Building Pendulum Cart

All the materials needed to build the above mentioned pendulum cart can be seen in Table. 6. Some of the components do have a public price information, thus we refer the reader to contact the corresponding manufacture and ask for a quote. As for some bolts and nuts, they are sold in packs. Thus, the price of one pack is listed and we indicate this using a "*" symbol. Besides the materials mentioned in Table. 6, we also recommend ordering 1074-1095 Spring Steel Ring Shim (McMaster 98055A117: 0.1mm thick, 10mm ID. McMaster 98055A121: 0.5mm Thick, 10mm ID) to serve as replacement of 3D printed shim (35) if the printer precision is not high.

Table 6: Materials needed to build the pendulum cart in Fig. 7 of the paper.

Designator	Component	Number	Total cost-currency	Source of materials	Material type
91251A077 (4)	McMaster 91251A077	6	8.74	McMaster	Black-Oxide Alloy Steel
Encoder (7)	US Digital EM2-2-10000-I	1	43.51	US Digital	Others

Table 6 continued from previous page

Designator	Component	Number	Total cost-currency	Source of materials	Material type
DriverClip (25)	3D Printed	1	NA	NA	PLA
CableDriver (26)	US Digital PC4-H10	3	51.09	US Digital	Others
96144A111 (27)	McMaster 96144A111	6	21.24	McMaster	Black-Oxide Alloy Steel
3329A310 (28)	McMaster 3329A310	1	28.1	McMaster	Chrome-Plated Brass and Others
CartPlate (29)	McMaster 9246K486	1	26.69	McMaster	Aluminum
LinearMotor (30)	HIWIN LMX1K -SA12-1-2000 -PGS1-V103+HS	1	NA	HIWIN	Aluminum and Others
61900 [6] (31)	61900 Hybrid Ceramic Bearing	2	81.9	Ortech	Ceramic, Steel, and Others
Spacer (32)	3D Printed	1	NA	NA	PLA
9140T273 (33)	McMaster 9140T273	1	39.23	McMaster	Aluminum
98055A122 (34)	McMaster 98055A122	1	10.69*	McMaster	1074-1095 Spring Steel
Shim (35)	3D Printed	1	NA	NA	PLA
EncoderDisk2 (36)	US Digital HUBDISK-2 -10000-394-IE	1	37.06	US Digital	Others
90326A105 (37)	McMaster 90326A105	1	6.35	McMaster	Steel
BlockBase (38)	3D Printed	1	NA	NA	NA
9125A471 (39)	McMaster 91251A471	2	8.35*	McMaster	Black-Oxide Alloy Steel
BrushBlock-8 (40)	Moog AC259-8	1	NA	Moog	Gold and Others
LimitSwitch Plate1 (41)	McMaster 89015K113	1	4.35*	McMaster	Aluminum
90751A110 (42)	McMaster 90751A110	4	8.86*	McMaster	18-8 Stainless Steel
LimitSwitch Plate2 (43)	McMaster 89015K113	1	4.35*	McMaster	Aluminum
6000N312 (44)	McMaster 6000N312	2	3.96	McMaster	Stainless Steel
LimitSwitch Base (45)	3D Printed	1	NA	NA	PLA
90592A085 (46)	McMaster 90592A085	4	1.44*	McMaster	Steel
91290A318 (47)	McMaster 91290A318	2	14.37*	McMaster	Steel
91290A121 (48)	McMaster 91290A121	2	14.33*	McMaster	Alloy Steel

[6] 61900 Hybrid Ceramic Bearings (10 X 22 X 6). Bearing Material: Chorme Steel 52100. Ball Material: Silicon Nitride. Cage/Retainer: PTFE. Enclosure: Open. Rating(ABEC): 7. Bearing Internal Clearance: Tight. Radial Play C2. Lube: Dry.

Table 6 continued from previous page

Designator	Component	Number	Total cost-currency	Source of materials	Material type
LimitSwitch (49)	Omron EE-SX671 (PNP)	2	64.06	Omron	Ohters

J..3 Bill of Materials for System Frame

The materials needed to build the system frame can be summarized in Table. 7. It is worth noting that component 56, 57, 62 and 63 already comes with fastener, so there's no need to purchase additional fasteners. Moreover, the component 50, 55, 58 and 61 can be directly purchased with the desired length by ordering McMaster 5537T907. This can ease the manufacturing process but will increase the overall price.

Table 7: Materials needed to build the system frame in Fig. 7.

Designator	Component	Number	Total cost-currency	Source of materials	Material type
Extrusion1-4 (50)	McMaster 5537T103-4	8	261.52	McMaster	6560 Aluminum
X-Connector (51)	McMaster 89015K255	4	63.4	McMaster	6061 Aluminum
AlignTool1 (52)	3D Printed	8	NA	NA	PLA
AlignTool2 (53)	3D Printed	8	NA	NA	PLA
Drop-In Nut (54)	McMaster 5537T271	16	34.56	McMaster	Zinc-Plated Steel
Extrusion2-4 (55)	McMaster 5537T103-4	4	130.76	McMaster	6560 Aluminum
47065T998 (56)	McMaster 47065T998	10	166.1	McMaster	Anodized Aluminum
47065T984 (57)	McMaster 47065T948	2	32.48	McMaster	Anodized Aluminum
Extrusion3 (58)	McMaster 5537T103-2	2	35.84	McMaster	6560 Aluminum
Extrusion4 (59)	Leftover material when producing (58)	2	NA	McMaster	6560 Aluminum
5537T323 (60)	McMaster 5537T323	20	101	McMaster	Zinc
Extrusion5 (61)	McMaster 5537T103-2	2	35.84	McMaster	6560 Aluminum
Wheel (62)	McMaster 4706T83	4	83.52	McMaster	Zinc-Plated Steel and Rubber
5537T37 (63)	McMaster 5537T37	4	105.76	McMaster	Anodized Aluminum
Extrusion6 (64)	McMaster 5537T103-6	2	92.54	McMaster	6560 Aluminum

J..4 Bill of Materials for Real-Time System

This section summarizes the materials needed to build the real-time system shown in Fig. 8 of the paper. All materials needed is shown in Table. 8. Since monitor (69) does not affect the entire

system's performance, we will not include the detailed information on which monitor we used. As long as the monitor the readers choose to use has a display port, it can connect with the target system. The host computer we used is quite old, so we decided not to put its price. The readers can use any computer they like as a host computer if it supports running Matlab and has an Ethernet port. As for the price of Speedgoat product, please send a quote to Speedgoat for more details.

Table 8: Materials needed to build the real-time system.

**Designator**	**Component**	**Number**	**Total cost-currency**	**Source of materials**	**Material type**
Terminal Block (65)	Speedgoat Terminal Board 2×17 Pin	2	NA	Speedgoat	Others
Cable (66)	M12M to M12F 17 Pin 3 feet Cable	4	NA	Speedgoat	Others
IO-191 (67 − 1)	IO-191 -Baseline	1	NA	Speedgoat	Others
IO-392 (67 − 2)	IO-392 -Baseline	1	NA	Speedgoat	Others
TargetMachine (67 − 3)	Baseline Education Real-Time Target Machine	1	NA	Speedgoat	Others
HostComputer (68)	NA	1	NA	NA	Others
Monitor (69)	NA	2	NA	NA	Others
5537T698 (70)	McMaster 5537T698	2	2.14	McMaster	Silver Zinc-Plated Steel
8961K102 (71)	McMaster 8961K102	1	3.9	McMaster	Zinc-Plated Steel
IO-392 DAQ4RS422 (72)	IO-392 Configuration File	1	NA	Speedgoat	NA

J..5 Bill of Materials for the Electronic Parts

This section summarize necessary components needed in the electrical system of the multi-link pendulum on the cart system. All the components needed in the electrical parts are shown in Table. 9. Some of the components needed such as cables, DIN rail, DIN rail terminal blocks , wires, etc, are not shown in Fig. 10 of the paper and is included in Table. 9.

Table 9: Materials needed to build the electrical part of the system.

**Designator**	**Component**	**Number**	**Total cost-currency**	**Source of materials**	**Material type**
EmergencyStop Module (75)	Powertec 71354	1	19.99	Powertec	Others

Table 9 continued from previous page

Designator	Component	Number	Total cost-currency	Source of materials	Material type
Circuit Breaker (76)	Eaton FAZ-B3 -2-NA	1	48	Automation Direct	Others
Magnetic Contactor (77)	WEG CWC016 -00-22V18	1	23.5	Automation Direct	Others
Noise Filter (78)	Schaffner FN2090 -10-06	1	31.5	Automation Direct	Others
Line Reactor (78)	LR-11P0 -1PH	1	139	Automation Direct	Others
Motor Drive (80)	HIWIN D1 Drive	1	NA	HIWIN	Others
Edge Filter (81)	HIWIN MF-40-S	1	NA	HIWIN	Others
Regenerative Resistor (82)	Yohii 500W 30 Ω Resistor	2	2.14	Amazon	Others
DC Supply (83)	MEAN WELL MDR-60-24	1	24.84	MEAN WELL	Others
Enclosure (84)	Hammond CSKO242410	1	171	Automation Direct	Carbon Steel
Toggle Switch (85)	20A 12VDC / 120VAC Toggle Switch	1	7.99	Amazon	Others
Momentary Switch (86)	LC LICTOP AC 660V Push Button Switch	1	6.99	Amazon	Others
EMI Cores (87)	HIWIN D1-EMC1	2	NA	HIWIN	Others
Enclosure Panel (88)	Hammond CSFC2424	1	21	Automation Direct	Carbon Steel
DIN Rail (89)	DN-R35S1-2	1	12.5	Automation Direct	Plated Steel
14AWG Cable (90)	Amazon 14 AWG Cable	1	NA	Amazon	Copper
12AWG Cable (91)	Amazon 12 AWG Cable	1	NA	Amazon	Copper
10AWG Cable (92)	Amazon 10 AWGCable	1	NA	Amazon	Copper
DIN Rail Terminal Block (93)	Amazon DIN Rail Block	10	NA	Amazon	Others

Table 9 continued from previous page

Designator	Component	Number	Total cost-currency	Source of materials	Material type
DIN Rail Ground Terminal Block (94)	Amazon DIN Rail Terminal Block	10	NA	Amazon	Others
DC Connector (95)	Wago 721-103/ 026-000	1	4.03	Newark	Others
AC Connector (96)	Wago 721-204/ 026-000	1	6.91	Digikey	Others
MotorPower Connector (97)	Wago 721-104/ 026-000	1	5.26	Digikey	Others
EdgeFilter Input Connector (98)	Phoenix Contact 1718517	1	11.3	Mouser Electronics Phoenix Contact 1718517	Others
EdgeFilter Output Connector (99)	Phoenix Contact 1718504	1	11.3	Mouser Electronics Phoenix Contact 1718504	Others
Regen Connector (100)	Wago 723-603	1	3.85	Galco	Others
Communication Cable (101)	D1-DNT07A USB232 to RJ11 Adapter Cable	1	22	Amazon	Others
HookUp Wire (102)	24 AWG Hook Up Wire	1	NA	Amazon	Copper
Shielding Wire (103)	Electriduct 1/2" Tinned Copper Metal Braid Sleeving Flexible EMI RFI Shielding Wire Mesh (0.32" Diameter) - 10 Feet	1	21.88	Amazon	Tinned Copper Metal
Cable Sleeve (104)	Keco 100ft – 1/2 inch PET Expandable Braided Cable Sleeve	1	16.99	Amazon	Others
CN2 Connector (105)	3M 10126-3000	1	11.62	Digikey	Others

Table 9 continued from previous page

Designator	Component	Number	Total cost-currency	Source of materials	Material type
CN2 Connector Cover (106)	3M 10326-52F0-008	1	10.62	Digikey	Others
Limit Switch Cable (107)	HIWIN D-Sub 9-Pin Female Limit Switch Extension Cable	1	NA	HIWIN	Others

References

[1] Kadierdan Kaheman, Eurika Kaiser, Benjamin Strom, J Nathan Kutz, and Steven L Brunton. Learning discrepancy models from experimental data. *arXiv preprint arXiv:1909.08574*, 2019.

[2] HIWIN. *Linear Motor System Technical Information*. HIWIN MIKROSYSTEM CORP, Taichung 40852, Taiwan, 2017.

[3] Roman Čečil, Vlastimil Šetka, and Miloš Schlegel. Radio module for fast real-time control of inverse triple pendulum. In *2016 3rd International Symposium on Wireless Systems within the Conferences on Intelligent Data Acquisition and Advanced Computing Systems (IDAACS-SWS)*, pages 80–84. IEEE, 2016.

[4] Vlastimil Šetka, Roman Čečil, and Miloš Schlegel. Triple inverted pendulum system implementation using a new ARM/FPGA control platform. In *2017 18th International Carpathian Control Conference (ICCC)*, pages 321–326. IEEE, 2017.

[5] Knut Graichen, Michael Treuer, and Michael Zeitz. Swing-up of the double pendulum on a cart by feedforward and feedback control with experimental validation. *Automatica*, 43(1):63–71, 2007.

[6] Tobias Glück, Andreas Eder, and Andreas Kugi. Swing-up control of a triple pendulum on a cart with experimental validation. *Automatica*, 49(3):801 – 808, 2013.

[7] Vladimir Kraz. Mitigating EMI issues in servo motors and variable frequency drives. *2016 EMC Test and Design Guide*, pages 9–16, 2016.

[8] HIWIN. *mega-fabs D1 Drive User Guide*. HIWIN MIKROSYSTEM CORP, Taichung 40852, Taiwan, 2011.

[9] Tobias Glück, Andreas Eder, and Andreas Kugi. Swing-up control of a triple pendulum on a cart with experimental validation. *Automatica*, 49(3):801–808, 2013.

[10] Audun D Myers, Joshua R Tempelman, David Petrushenko, and Firas A Khasawneh. Low-cost double pendulum for high-quality data collection with open-source video tracking and analysis. *HardwareX*, 8:e00138, 2020.

[11] Benjamin Jahn, Lars Watermann, and Johann Reger. On the design of stable periodic orbits of a triple pendulum on a cart with experimental validation. *Automatica*, 125:109403, 2021.
